# Design, Synthesis, and Biological Evaluation of Novel Hydroxamic Acid-Based Organoselenium Hybrids

**DOI:** 10.3390/ph16030367

**Published:** 2023-02-28

**Authors:** Jameelah S. Alotaibi, Yasair S. Al-Faiyz, Saad Shaaban

**Affiliations:** 1 Chemistry Department, College of Science, King Faisal University, Al-Ahsa 31982, Saudi Arabia; 2 Chemistry Department, Faculty of Science, Mansoura University, Mansoura 35516, Egypt

**Keywords:** anticancer, hydroxamic acid, antimicrobial, organoselenium, antioxidant

## Abstract

We report the design and synthesis of novel hydroxamic acid-tethered organoselenium (OSe) hybrids. Their antimicrobial and anticancer activities were assessed against different microbes (e.g., *Candida albicans* (*C. albicans*), *Escherichia coli* (*E. coli*), and *Staphylococcus aureus* (*S. aureus*)), as well as liver and breast carcinomas. OSe hybrid **8** showed promising anticancer activity, with IC_50_ = 7.57 ± 0.5 µM against HepG2 and IC_50_ = 9.86 ± 0.7 µM against MCF-7 cells. Additionally, OSe compounds **8** and **15** exhibited promising antimicrobial activities, particularly against *C. albicans* (IA% = 91.7 and 83.3) and *S. aureus* (IA% = 90.5 and 71.4). The minimum inhibitory concentration (MIC) assay confirmed the potential antimicrobial activity of OSe compound **8**. OSe compounds **8** and **16** displayed good antioxidant activities compared to vitamin C in the 2,2-diphenyl-1-picrylhydrazyl (DPPH) and the 2,2′-azino-bis (3-ethylbenzothiazoline-6-sulfonic acid (ABTS) assays. These results indicate that hydroxamic acid-based organoselenium hybrids have promising biological activities such as anticancer, antimicrobial, and antioxidant properties, especially compounds **8**, **13**, **15,** and **16,** which warrant further studies.

## 1. Introduction

Hydroxamic acids (HAs) are among the most important families of organic compounds with general structures R–CO–NHOH and R–CO–NR′OH, where R, R′ represent various organic residues [1,2].

**Figure 1:**
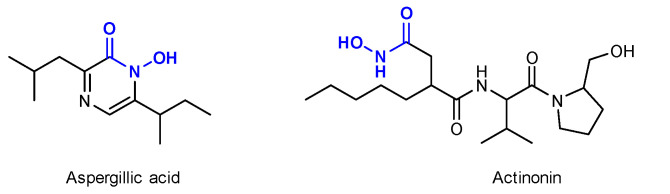
Natural occurring hydroxamic acids aspergillic acid and actinonin.

They are distributed throughout nature. Many naturally occurring hydroxamic acids have been isolated and examined for their biological activities. For example, Aspergillic acid and Actinonin have antibiotic activity (Figure 1) [3].

Hydroxamic acid moiety has gained significant attention from research groups worldwide. Therefore, many hydroxamic derivatives have been synthesized and examined for their biological activities and chemistry applications [4,5,6].

Furthermore, HAs play a distinct role as potent chelating agents, due to the bidentate group that form complexes with various metals, including transition metals [7,8,9].

**Figure 2:**
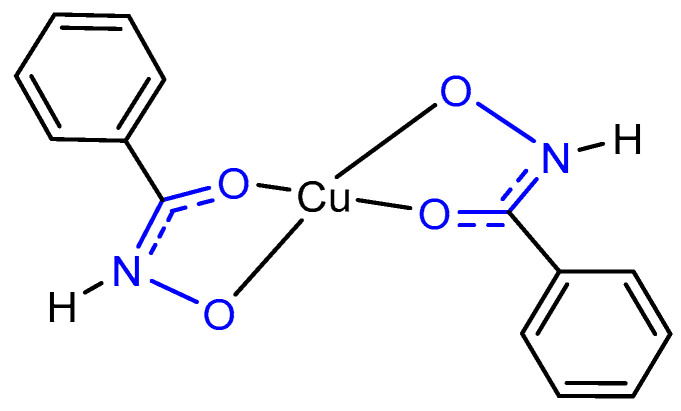
Hydroxamic acid ability to chelate metal ions such as the copper ions.

Thus, the chelating propriety of hydroxamic acids with metal ions gives them the ability to interact with several metal-containing enzymes, with vast arrays of biological activities. Their solid chelating ability has been employed in metalloenzyme (e.g., Figure 2) [10] inhibition [11] via a metal binding group with an active site (i.e., HDAC, MMP, urease, lipoxygenase, angiostatin) (Figure 2) [12,13]. Furthermore, this chelating ability has been employed in environmental remediation treatments and organic synthesis methods [14]. It is also well documented that they have broad biological and pharmacological effects, including antibacterial, antimicrobial, antifungal, antimalarial, antitumor, anticancer, antituberculosis, and antimalaria properties, and treatments for cardiovascular diseases and iron overload [15,16,17,18,19].

Many analogous hydroxamic acids have been reported. For example, sulfur-based hydroxamic acids have been investigated, and their applications have been documented [20,21,22,23,24]. Surprisingly, there is less focus on hydroxamic acid-based selenium (Se) compounds and their hydroxamic acid derivatives. In fact, and to our knowledge, the only examples related to hydroxamic acid-based Se in the literature are the modified HA (I) compounds to SeHA (II) and SeHA (III) [25,26]; these studies have developed and evaluated anticancer activity, and these compounds were found to be more potent than HA (I) [25,26].

Se is an essential microelement in the human body. It is a non-metal that belongs to group 16 of the periodic table and lies between sulfur and tellurium [27,28]. Se can be found in several dietary sources, such as seafood, organ meats, and Brazil nuts, or as supplements [26,29]. Se deficiency results in severe consequences, such as increasing the incidence of cancer and heart diseases. On the other hand, elevated Se concentrations may be lethal to the human body. Accordingly, maintaining the Se concentrations at their normal levels is critical in protecting the immune system and preventing cancer [30,31,32].

Therefore, scientists have paid much attention to the development of Se chemistry, owing to its diverse applications, which are not only limited to cancer therapy but also extend to electronics (e.g., optoelectronics, photocells, and solar cells) [31,32]. Moreover, OSe compounds have recently become a hot spot in biochemical processes compared to inorganic Se compounds, since they are less toxic and have higher bioavailability [30,31,32]. As a result, numerous studies have focused on synthesizing new OSe compounds with potential cancer chemopreventive, antioxidant, antitumor, and antiviral activities [26,27,28,29].

**Figure 3:**
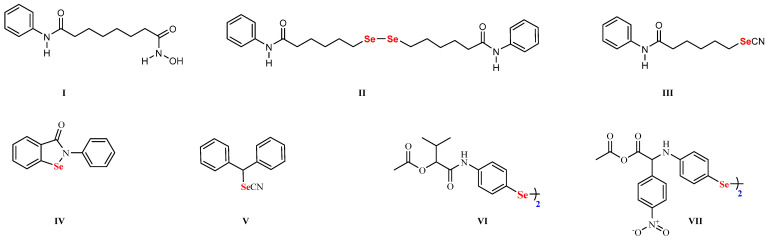
Diverse organoselenium compounds.

In this context, ebselen (IV) has shown anti-inflammatory, antioxidant, and glutathione peroxidase-like activities as a neuroprotective agent (Figure 3) [27,28]. Furthermore, selenocyanate V has demonstrated good chemoprotective and chemopreventive activities toward various cancers. Moreover, the diselenide compounds (VI) and (VII) synthesized within our laboratory have displayed great cytotoxic potential against liver cancer cells (Figure 3) [29].

Therefore, combining OSe compounds with HAs is expected to enhance their overall biological activity. Within the context of this paper, we aimed to synthesize novel hydroxamic acid-based Se compounds (Has–Se) and evaluate them for biological activities such as antimicrobial, anticancer, and antioxidant properties.

## 2. Results and Discussions

### 2.1. Synthesis

**Scheme 1:**
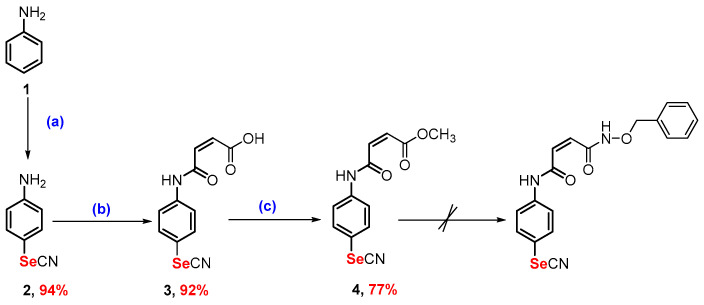
(a) Aniline (18.68 mmol, 1.69 mL), SeO_2_ (27 mmol, 3 g), malononitrile (12.11 mmol, 800 mg), DMSO (8 mL); (b) 4-selenocyanatoaniline (**2**) (2.5 mmol, 500 mg), maleic anhydride (3.19 mmol; 313 mg); toluene (10 mL); (c) 4-Oxo-4-((4-selenocyanatophenyl)amino)but-2-enoic acid (**3**) (1 mmol; 295 mg), methanol (10 mL), H_2_SO_4_ (400 μL).

The combination of Se and HAs affords unique pharmacophores with possible applications in different fields. Hydroxamic acids are generally obtained from the condensation of carboxylic acids and hydroxylamine. Therefore, Se-containing carboxylic acid **3** was used as a key starting material. We synthesized 4-Oxo-4-((4-selenocyanatophenyl)amino)but-2-enoic acid (**3**) at 92% from the reaction of corresponding 4-selenocyanatoaniline (**2**) and maleic anhydride (Scheme 1). An esterification reaction activated the Se-containing carboxylic acid 3. Within this context, we obtained a 77% yield of methyl-4-oxo-4-((4-selenocyanatophenyl)amino)but-2-enoate (**4**) by **3′s** reaction with methanol and H_2_SO_4_ (catalytic amounts). Unfortunately, the reaction of **4** with *o*-benzylhydroxylamine failed, and we could not isolate the desired products (Scheme 1).

**Scheme 2:**
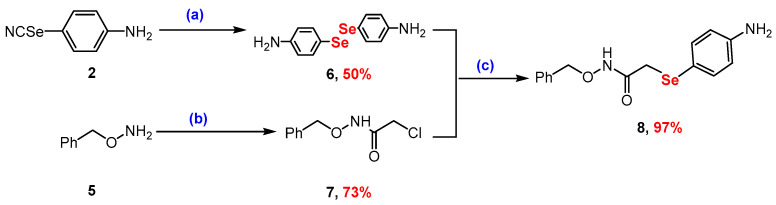
Synthesis of hydroxamic acid **8**. Reagents and conditions: (a) 4-selenocyanatoaniline **2** (2.5 mmol, 500 mg), NaOH (2.5 mmol, 100 mg), EtOH (5 mL); (b) *o*-benzyl hydroxylamine. HCl (9.39 mmol, 1.5 g), DCM (35 mL), Et_3_N (11.27 mmol, 1.75 mL); chloroacetyl chloride (10.34 mmol, 0.822 mL); (c) 4-*N*-(benzyloxy)-2-chloroacetamide (2 mmol, 398 mg), 4,4′-diselanediyldianiline (1 mmol, 342 mg) NaBH_4_ (6.60 mmol, 250 mg).

The reaction of *o*-benzylhydroxylamine hydrochloride’s with chloroacetyl chloride created a 73% yield for 4-*N*-(benzyloxy)-2-chloroacetamide (**7**). We prepared 2-((4-aminophenyl)selanyl)-*N*-(benzyloxy)acetamide (**8**) reacting **7** with sodium benzeneselenolate, formed in situ by reduced4,4′-diselanediyldianiline with NaBH_4_ (Scheme 2).

The structure of compound **8** was proved by the IR, which showed absorption bands at 3452 and 3343 cm^−1^ for the NH_2_ and NH groups and 1648 cm^−1^ for the C=O group. The ^1^H NMR for compound **8** showed two singlet signals at 4.71 ppm and 3.21 ppm for the two methylene groups, i.e., CH_2_Se and CH_2_O. The mass spectrum exhibited molecular ion peaks at 336.20 (M^+^, 10.01) and the base peak at *m*/*z* 91 for the benzyl residue.

**Scheme 3:**
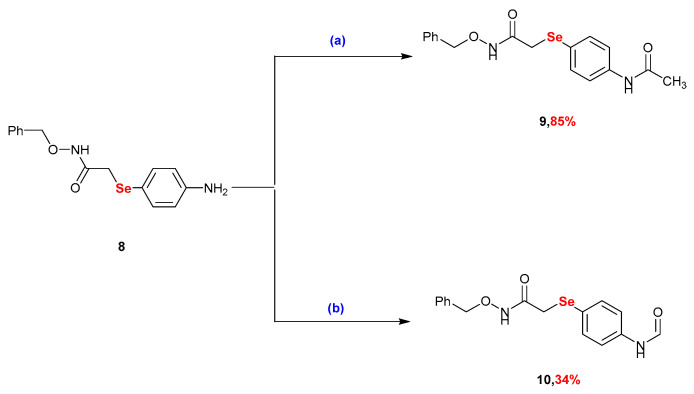
Synthesis of compounds **9** and **10**. Reagents and conditions: (a) 2-((4-aminophenyl)selanyl)-*N*-(benzyloxy)acetamide (**8**) (0.62 mmol, 208 mg), acetic anhydride (0.084 mmol, 8 mL), NaOAc (6.1 mmol, 500 mg); 60–65 °C for 2 h; (b) 2-((4-aminophenyl)selanyl)-*N*-(benzyloxy)acetamide (**8**) (1 mmol, 335.27 mg), THF (2.5 mL), acetic formic anhydride (0.68 mmol, 60 µL).

Once the selenium-based HA **8** was prepared, our focus changed to delivering a range of diverse structures via further derivatization. Within this context, the reaction of **8** with acetic anhydride and acetic formic mixed anhydride created the yields of 85% acetanilide **9** and 34% formamide (Scheme 3).

The structure of compound **9** was proved by the IR, which showed absorption bands at 3297 and 3258 cm^−1^ for the two NH groups and 1697 and 1665 cm^−1^ for the two C=O groups. The ^1^H NMR for compound **9** showed three singlet signals at 4.92, 3.82, and 2.02 ppm for the CH_2_O, CH_2_Se, and CH_3_ groups, respectively. The mass spectrum exhibited molecular ion peaks at 378.20 (M^+^, 0.04) and the base peak at *m*/*z* 91 for the benzyl residue (see the Supporting Materials). Compound **10** was proved by the IR, which showed absorption bands at 3298 and 3193 cm^−1^ for the two NH groups and 1706 and 1682 cm^−1^ for the two C=O groups. The ^1^H NMR for compound **10** manifested five singlet signals at 11.18, 10.30, 8.31, 4.74, and 3.40 ppm for the NHO, CHO, NH, CH_2_O, and the CH_2_Se groups, respectively. The mass spectrum exhibited molecular ion peaks at 364.25 (M^+^, 1.43) and the base peak at *m*/*z* 91 for the benzyl residue (see Supporting Materials).

**Scheme 4:**
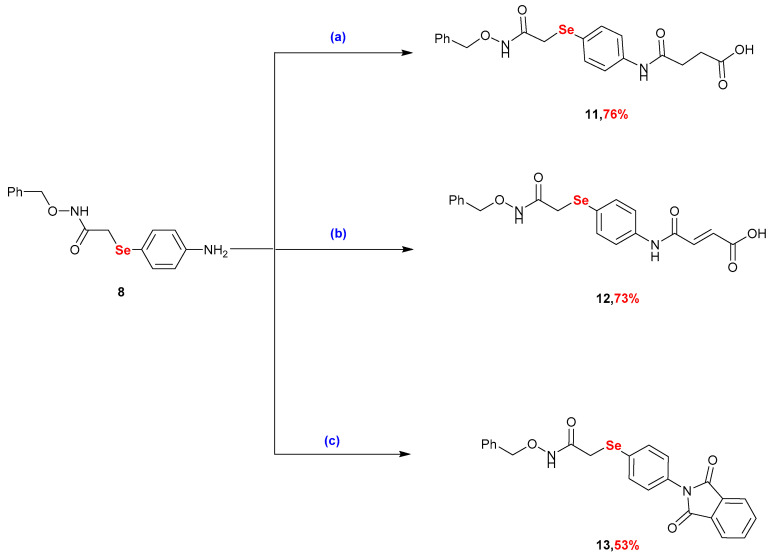
Synthesis of compounds **11**, **12,** and **13**. Reagents and conditions: (a) succinic anhydride (1.64 mmol), 2-((4-aminophenyl)selanyl)-*N*-(benzyloxy)acetamide (**8**) (1.49 mmol, 500 mg), and toluene (20 mL); (b) maleic anhydride (1.64 mmol), 2-((4-aminophenyl)selanyl)-*N*-(benzyloxy)acetamide (**8**) (1.49 mmol, 500 mg), and toluene (20 mL); (c) 2-((4-aminophenyl)selanyl)-*N*-(benzyloxy)acetamide (1 mmol, 335.27 mg), phthalic anhydride (1 mmol, 148 mg), sodium acetate anhydrous (1 g), acetic acid (10 mL), reflux, 6 h.

The reaction of **8** with succinic and maleic anhydrides created the corresponding *N*-succinalinic and *N*-mealnilinic acids **11** and **12,** giving 76 and 73% yields, respectively (Scheme 4). By contrast, the reaction of **8** with phthalic anhydride produced a 53% yield of phthaloyl derivative **13** (Scheme 4).

The structure of compound **11** was proved by the ^1^H NMR, which showed five singlet signals at 12.18, 11.16, 10.07, 4.73, 3.37 ppm for the COOH, NH, NH, CH_2_O, and CH_2_Segroups, respectively (see Supporting Materials). Compound **12** showed characteristic two doublets of doublet signals for the two olefinic carbons at 6.47 and 6.35 ppm. In the ^13^CNMR, the three carbonyl groups signals appeared at 167.39, 166.84, and 163.76 ppm (see the Supporting Materials). The phthaloyl derivative **13** manifested two singlet signals at 4.88 and 3.51 ppm for the two methylene groups, CH_2_O and CH_2_Se, respectively, whereas they appeared at 77.31 and 26.70 ppm in the ^13^CNMR.

**Scheme 5:**
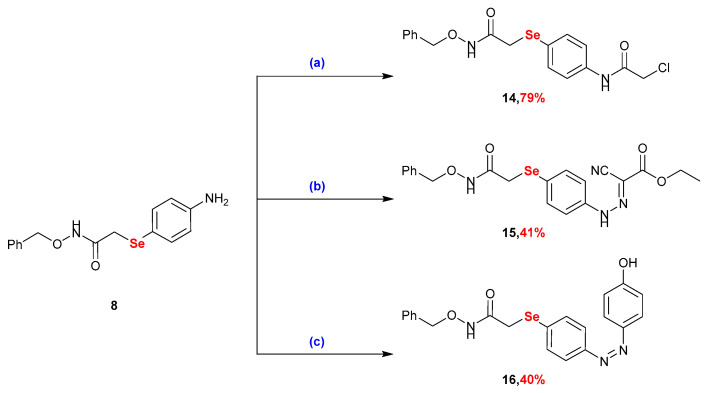
Synthesis of compounds **14**, **15**, and **16**. Reagents and conditions: (a) 2-((4-aminophenyl)selanyl)-*N*-(benzyloxy)acetamide (**8**) (2 mmol, 670.54 mg ), potassium carbonate (36.17 mmol, 5 g), acetone (30 mL), chloroacetyl chloride (5 mmol, 397.67 µL); (b) 2-((4-aminophenyl)selanyl)-*N*-(benzyloxy)acetamide (**8**) (1 mmol, 335.27 mg), H_2_SO_4_ (1.5 mL), sodium nitrite (1.2 mmol, 82.79 mg), ethyl cyanoacetate (1.2 mmol, 135.74 mg), and sodium acetate (1 g); (c) 2-((4-aminophenyl)selanyl)-*N*-(benzyloxy)acetamide (**8**) (1 mmol, 335.27 mg), HCl (1.5 mL), sodium nitrite (2.2 mmol, 164.5 mg), phenol (1.2 mmol, 112.93 mg) and sodium hydroxide 10%, 5 mL.

The reaction of **8** with chloroacetyl chloride created chloroacetamide **14** with a 79% yield (Scheme 5). Diazotization of the selenium-based HA **8** and the succeeding coupling with ethyl cyanoacetate and phenol created the diazo-based derivatives **15** and **16,** with 41 and 40% yields, respectively (Scheme 5).

The structure of compound **14** was proved by the IR, which showed absorption bands at 3202 and 3176 cm^−1^ for the two NH groups and 1652 and 1606 cm^−1^ for the two C=O groups. The ^1^H NMR for compound **14** showed three singlet signals at 4.73, 4.27, and 2.52 ppm for the CH_2_O, CH_2_Se, and the CH_2_Cl, respectively. The mass spectrum exhibited molecular ion peaks at 412.20 (M^+^ + 1) and the base peak at *m*/*z* 91 for the benzyl residue (see Supporting Materials). The structure of compound **15** was confirmed by the IR, which showed absorption bands at 3218 and 3176 cm^−1^ for the two NH groups and 1685 and 1645 cm^−1^ for the two C=O groups. The ^1^H NMR for compound **15** showed three singlet signals at 4.75 and 3.39 ppm for the CH_2_O and CH_2_Se, respectively. The ethyl group showed a distinctive pattern in the ^1^HNMR as quartet and triplet at 4.29 and 1.31 ppm for the CH_2_ and the CH_3_ groups, respectively. The mass spectrum exhibited molecular ion peaks at 460.20 (M^+^ + 1) and the base peak at *m*/*z* 91 for the benzyl residue (see Supporting Materials).

### 2.2. Biology

#### 2.2.1. Evaluation of the Cytotoxicity of the OSe-Based HAs Compounds

Recently, OSe agents have attracted interest due to their potential chemoprotective and antitumor properties [30,31,32,33]. We have developed several OSe compounds with promising antioxidant, antimicrobial, and antitumor properties [34,35,36,37,38]. Therefore, we estimated the OSe agents’ antiproliferative activities against different cancer cells, i.e., MCF-7 and HepG2 cells and healthy lung fibroblast WI-38 cells, using the MTT assay. The drug doxorubicin was employed as the standard. The minimal inhibition dose causing death to 50% of cells was also estimated and is presented in Table 1. The corresponding therapeutic indices (TI) are described as the proportion of the WI38 cells IC_50_ to the IC_50_ of the cancer cell (e.g., MCF-7 and HepG2) (Table 1) [10,11,12,13].

Interestingly, OSe hybrids were more cytotoxic to HepG2 cells than MCF-7 cells. For example, OSe compound **8** exhibited promising anticancer activity with IC_50_ = 7.57 ± 0.5 µM against HepG2 and IC_50_ = 9.86 ± 0.7 µM against MCF-7 cells, respectively (Table 1). OSe compound **15** also demonstrated anticancer activity with IC_50_ = 15.83 ± 1.3 µM against HepG2 and IC_50_ = 21.58 ± 1.6 µM against MCF-7 cells, respectively (Table 1).

TI values were more evident in the case of HepG2 cells than in MCF-7 cells. For instance, OSe compounds **15** and **8** manifested TI values of 5.2 and 3.6 in the case of HepG2 cells and 3.8 and 2.8 in the case of MCF-7, respectively. Ultimately, these promising, selective antitumor properties are worthy of further studies that employ a more comprehensive panel of normal and cancer cells and in vivo experiments.

#### 2.2.2. Estimation of the Antimicrobial Properties of the OSe-Based HAs Compounds

The encouraging antitumor properties of the OSe compounds motivated us to evaluate their corresponding antimicrobial activities against the *C. albicans* fungal strain, *S. aureus* Gram-positive bacteria, and *E. coli* Gram-negative bacteria. Therefore, we applied the agar diffusion and minimum inhibitory concentration (MIC) methods using clotrimazole antifungal and ampicillin antibacterial drugs as standards. The diameters of the inhibition zones (ZID) (mm) and the percentage activity index (IA%) are presented in Table 2.

In general, the antimicrobial activities were more pronounced in the case of *S. aureus* Gram-positive bacteria and *C. albicans* fungus. For instance, OSe agents **8** and **15** exhibited promising antimicrobial activities against *C. albicans* (IA% = 91.7 and 83.3) and *S. aureus* (IA% = 90.5 and 71.4) (Table 2).

We evaluated the MIC of the most active OSe compound, **8** (Table 3) to further explore its potential activity. OSe compound **8** showed antimicrobial activities with MIC of 4 μM against *C. albicans*, MIC of 4 μM against *S. aureus*, and MIC of 8 against *E. coli* strains, respectively.

#### 2.2.3. Evaluation of the Antioxidant Properties of the OSe-Based HAs Compounds

The antioxidant activities of the OSe compounds were intensively investigated because these are usually the cause of their chemopreventive potency [34,39]. Their redox activities were evaluated via DPPH and ABTS assays, using ascorbic acid as the standard [40,41]. We spectrophotometrically measured the antioxidant efficiency of the OSe compounds by their ability to decolorise the DPPH**^.^** and ABTS**^.^** radicals at 734 and 517 nm, respectively (Figure 2).

**Figure 4:**
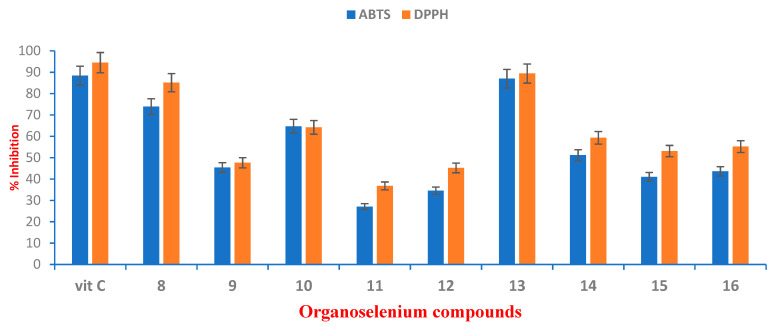
Estimation of the OSe compounds’ redox activities via DPPH and ABTS experiments. All tests are mean ± S.D. ABTS experiment: absorbance was followed at 734 nm for OSe compounds (50 µL, 1 mM in MeOH) and ABTS solution (60 µL). DPPH Absorbance was monitored at 517 nm for OSe compounds (200 µL, 1 mM in MeOH) and DPPH (400 µL) after 30 min.

As shown in Figure 2, products **8**, **10**, and **13** showed 73.9, 64.7, and 87.0% antioxidant activities, respectively, compared with 88.4% by vitamin C in the ABTS assay. Similarly, products **8**, **10**, and **13** exhibited 85.1, 64.2, and 89.4% antioxidant activities, respectively, whereas vitamin C showed 94.5% in the DPPH assay (Figure 4).

These findings encouraged us to estimate the activity of the most active OSe compound, **8,** via its respective minimum concentrations, which caused a 50% decrease in the absorbance of the ABTS and DPPH assays, respectively (Table 4). Interestingly, OSe compound **8** has antioxidant, antimicrobial, and anticancer activities, suggesting its potential biological activities.

## 3. Materials and Methods

### 3.1. Experimental

Melting points were calculated using Gallenkamp apparatus and were uncorrected. Elemental analyses were carried out at Cairo University. The IR spectra were measured using an Agilent Technologies Cary 630 FTIR instrument at King Faisal University. Mass spectra were recorded at Cairo University on a GC-MS-QP-100 EX Shimadzu instrument. The 1H and 13C NMR spectra were recorded on DMSO-*d*_6_ at King Faisal University or Mansoura University using a Varian Spectrophotometer at 400 MHz. Chemical shifts were recorded in parts per million (ppm), and TMS (0.00 ppm) was used as a reference. Coupling constants (J) are reported in hertz (Hz). Biological experiments were conducted at the Faculty of Pharmacy, Mansoura University. Compound numbers **2**, **3**, and **6**, **7** were synthesised according to previously documented procedures [5,42,43,44,45,46].

### 3.2. Chemistry

#### 3.2.1. Synthesis of Methyl (Z)-4-Oxo-4-((4-selenocyanatophenyl)amino)but-2-enoate (**4**)

We added 4-oxo-4-((4-Selenocyanatophenyl)amino)but-2-enoic acid (295.15 mg, 1 mmol) to MeOH (10 mL). We followed this step by adding concentrated H_2_SO_4_ (400 μL) and stirring the mixture at room temperature for 5 h and 30 min. Then, we poured the mixture onto ice and collected the resulting precipitate without purification.

This resulted in the production of greyish silver crystals, which yielded 237 mg (77%); mp: 152 °C; Rf = 0.266 [Pet. ether: EtOAc (4:4)]; I.R. (KBr) λ_max_. cm^−1^: 3100, 2230, 1727, 1660; ^1^H NMR (400 MHz, DMSO) δ 10.51(s, 1H, NH), 7.69 (d, 4H, Ar-H), δ 6.50 (d, *J* = 28.8 Hz, 2H, CH=CH), 3.68 (s, 3H, CH_3_);^13^C NMR (101 MHz, DMSO) δ 166.39, 162.91, 139.88, 134.81, 132.21, 128.96, 120.61, 117.09, 105.36, 51.62; M.S. (E.I., 70 ev) *m*/*z* (%) = 310.10 (M^+^, 4.44), 85.05 (17.87), 91.05 (5.68), 59 (11.97), 113.10 (100.0, base peak), 69 (12.47).

#### 3.2.2. Synthesis of N-(benzyloxy)-2-chloroacetamide (**7**)

We added Et_3_N (1.75 mL, 11.27 mmol) to a solution of *o*-benzylhydroxylamine HCl (1.5 g, 9.39 mmol) in DCM (35 mL) at room temperature and stirred the mixture for 10 min. Afterwards, the reaction mixture was cooled to 0 °C. Chloroacetyl chloride (0.822 mL, 10.34 mmol) was added dropwise to the reaction mixture and stirred for 5 h at 0 °C. The reaction was washed with brine and diluted with DCM (40 mL). The resulting mixture was washed with brine (3 × 50 mL) and dried over MgSO_4_. The compound was isolated as a white solid that yielded 1.36 g (73%); Rf = 0.35 [Pet. ether: EtOAc (4:4)]; MP: 90 °C. I.R. (KBr) λ_max_. cm^−1^: 3153, 1695.44, 1650.39; ^1^H NMR (400 MHz, DMSO) δ 11.55 (s, 1H, NH), 7.39 (m, 5H, Ar-H), 4.83 (s, 2H, CH_2_O), 3.98 (s, 2H, CH_2_Cl); ^13^C NMR (101 MHz, DMSO) δ 163.04, 135.63, 128.84, 128.63, 128.33, 76.86, 40.29; M.S. (E.I., 70 ev) *m*/*z* (%) = 201.15 (M^+^ + 2, 2), 131.15 (6.46), 117 (26.27), 103 (22.44), 101 (18.63), 91 (0.63), 87 (16.60), 59 (100.0, base peak).

#### 3.2.3. Synthesis of 2-((4-Aminophenyl)selanyl)-N-(benzyloxy)acetamide (**8**)

*N*-(Benzyloxy)-2-chloroacetamide (398 mg, 2 mmol) and 4,4′-diselanediyldianiline (342 mg, 1 mmol) were dissolved in ethanol (40 mL) at room temperature, then sodium borohydride (250 mg, 6.60 mmol) was gradually added. TLC followed the reaction progress. After completion, 35 mL of water was added. Then, the product was extracted by DCM (2 × 20 mL) and dried with MgSO_4_. The product was isolated as a brown solid, which yielded 327 mg (97%); Rf = 0.53 [DCM: MeOH (95:5)]; MP: 100 °C. I.R. (KBr) λ_max_. cm^−1^: 3452, 3343, 3208, 1648, 1590; ^1^H NMR (400 MHz, DMSO) δ 11.07 (s, 1H, NH), 7.40–7.33 (m, 5H, Ar-H), 7.24 (d, *J* = 7.7 Hz, 2H, Ar-H), 6.52 (d, *J* = 7.7 Hz, 2H, Ar-H), 5.45 (s, 2H, NH_2_), 4.71 (s, 2H, CH_2_O), 3.21 (s, 2H, CH_2_Se); ^13^C NMR (101 MHz, DMSO) δ 166.60, 148.72, 135.90, 135.83, 128.80, 128.29, 128.22, 114.63, 112.59, 76.78, 27.88; M.S. (E.I., 70 ev) *m*/*z* (%) = 336.20 (M^+^, 10.01), 172 (6.38), 106.15 (16.69), 91 (100.0, base peak), 80.10 (9.92), 77.10 (9.26), 59.05 (0.40) 57.05 (0.81).

#### 3.2.4. Synthesis of 2-((4-Acetamidophenyl)selanyl)-N-(benzyloxy)acetamide (**9**)

We gently heated 2-((4-Aminophenyl)selanyl)-*N*-(benzyloxy)acetamide (**8**) (208 mg, 0.62 mmol) with NaOAc (500 mg, 6 mmol) at 60–65 °C in acetic anhydride (8 mL) for 2 h. The mixture was poured onto ice, and the pH was neutralised to 7 by adding sodium carbonate. The formed precipitate was then filtered off and washed with water. The product was isolated as a reddish pink powder, which yielded 198.5 mg (85%); Rf = 0.52 [chloroform: MeOH (95:5)]; MP: 108–107 °C. I.R. (KBr) λ_max_. cm^−1^: 3297, 1697, 1665; ^1^H NMR (400 MHz, CDCl_3_) δ 7.45 (t, *J* = 8.9 Hz, 2H, Ar-H), 7.40–7.29 (m, 7H, Ar-H), 4.92 (s, CH_2_O), 3.82 (s, 2H, CH_2_Se), 2.10 (s, 3H, CH_3_); ^13^C NMR (101 MHz, CDCl_3_) δ 168.88, 168.43, 138.22, 135.16, 133.58, 129.79, 129.40, 128.81, 123.48, 120.41, 78.28, 31.74, 24.64; M.S. (E.I., 70 ev) *m*/*z* (%) = 378.20 (M^+^, 0.04), 170.05 (2.05), 106.15 (10.91), 91 (100.0, base peak), 80.10 (2.96), 77.10 (8.81), 65.05 (6.77), 59.05 (0.14) 58.05 (0.12) 57.05 (0.68).

#### 3.2.5. Synthesis of N-(benzyloxy)-2-((4-formamidophenyl)selanyl)acetamide (**10**)

We dissolved 2-((4-Aminophenyl)selanyl)-*N*-(benzyloxy)acetamide (**8**) (335.27 mg, 1 mmol) in 2.5 mL THF. Freshly prepared acetic formic anhydride (60 µL, 0.68 mmol) was added dropwise. The reaction was stirred at room temperature and monitored via TLC. After the complete reaction, the mixture was extracted with chloroform, washed with water, and dried over MgSO_4_. Then, the oily product was rewashed with petroleum ether (2 × 10 mL) and 10 mL of ether to produce a white solid, which yielded 124.9 mg (34%); Rf = 0.34 [chloroform: MeOH 5%]; MP: 92 °C. I.R. (KBr) λ_max_. cm^−1^: 3193, 1649, 1510; ^1^H NMR (400 MHz, DMSO) δ 11.18 (s, 1H, NHO), 10.30 (s, 1H, CHO), 8.31 (s, 1H, NH), 7.54 (dd, *J* = 19.5, 8.6 Hz, 4H, Ar-H), 7.38 (dd, *J* = 10.1, 4.7 Hz, 5H), 7.16 (d, *J* = 8.3 Hz, 1H, Ar-H), 4.74 (s, 2H, CH_2_O), 3.40 (s, 2H, CH_2_Se); ^13^C NMR (101 MHz, DMSO) δ 166.84, 160.18, 138.21, 136.35, 134.66, 134.03, 129.33, 128.81, 123.71, 120.30, 118.53, 77.31, 27.35; M.S. (E.I., 70 ev) *m*/*z* (%) = 364.25 (M^+^, 1.43), 200.05 (2.01), 171.00 (1.81), 107.15 (3.69), 91.10 (100.0, base peak), 80.05 (8.76), 77.05 (15.38), 65.05 (11.49), 59.05 (39.53), 57.05 (3.88).

General procedure for the synthesis of *N*-succinalinic acids **11** and *N*-mealnilinic **12**.

To a well-stirred solution of amine (1.49 mmol) in 20 mL toluene, the appropriate anhydride of 1.64 mmol (maleic anhydride or succinic anhydride) was added to the reaction vessel. At room temperature, the mixture was stirred for 4 h. Afterwards, the formed precipitate was separated by filtration and washed with toluene.

#### 3.2.6. Synthesis of 4-((4-((2-((Benzyloxy)amino)-2-oxoethyl)selanyl)phenyl)amino)-4-oxobutanoic Acid (**11**)

Compound **11** was synthesised following general procedure I from 2-((4-aminophenyl)selanyl)-*N*-(benzyloxy)acetamide (**8**) (500 mg, 1.49 mmol) and succinic anhydride (164.11 mg, 1.64 mmol). Its formation was monitored by TLC (DCM: MeOH 5%). Compound **11** was isolated as a beige solid, which yielded 493.2 mg (76%); Rf = 0.36; MP: 167–168 °C. I.R. (KBr) λ_max_. cm^−1^: 3273, 1650, 1593; ^1^H NMR (400 MHz, DMSO) δ 12.18 (s, 1H, COOH), 11.16 (s, 1H, NH), 10.07 (s, 1H, NH), 7.55 (d, *J* = 8.6 Hz, 2H, Ar-H), 7.47 (d, *J* = 8.5 Hz, 2H, Ar-H), 7.40–7.34 (m, 5H, Ar-H), 4.73 (s, 2H, CH_2_O), 3.37 (s, 2H, CH_2_Se), 2.60–2.55 (m, 4H, CH_2_CH_2_); ^13^C NMR (101 MHz, DMSO) δ 174.32, 170.70, 166.85, 139.35, 136.35, 133.97, 129.31, 128.80, 128.77, 122.76, 120.01, 77.28, 31.54, 29.23, 27.40; M.S. (E.I., 70 ev) *m*/*z* (%) = 336.20 (M^+^- C.O. (C.H.)_2_COOH, 1.65), 170 (10.97), 106.10 (12.15), 91 (100.0, base peak), 87.05 (4.02), 80.05 (4.33), 77.10 (10.71), 65.00 (7.15), 59.05 (14.73), 57.05 (0.72).

#### 3.2.7. Synthesis of 4-((4-((2-((Benzyloxy)amino)-2-oxoethyl)selanyl)phenyl)amino)-4-oxobut-2-enoic Acid (**12**)

Compound **12** was synthesised following general procedure I from 2-((4-aminophenyl)selanyl)-*N*-(benzyloxy)acetamide (**8**) (500 mg, 1.49 mmol) and maleic anhydride (160.81 mg, 1.64 mmol). Its formation was monitored by TLC (DCM: MeOH 5%). Compound **12** was isolated as greenish yellow solid, which yielded 472 mg (73%); Rf = 0.25; MP: 159–160 °C. I.R. (KBr) λ_max_. cm^−1^: 3283, 1705, 1660; ^1^H NMR (400 MHz, DMSO) δ 13.09 (s, 1H, COOH), 11.19 (s, 1H, NH), 10.46 (s, 1H, NH), 7.59 (d, *J* = 8.4 Hz, 2H, Ar-H), 7.38 (d, *J* = 7.3 Hz, 5H, Ar-H), 6.47 (dd, 1H, = C.H.), 6.35 (dd, 1H, C.H. =), 4.74 (s, 2H, CH_2_O), 3.40 (s, 2H, CH_2_Se);^13^C NMR (101 MHz, DMSO) δ 167.39, 166.84, 163.76, 138.55, 136.34, 133.79, 132.06, 130.84, 129.33, 128.81, 124.09, 120.58, 77.29, 27.30; M.S. (E.I., 70 ev) *m*/*z* (%) = 336.20 (M^+^- C.O. (C.H.)_2_COOH, 10.01), 170 (11.03), 106.10 (19.92), 91 (100.0, base peak), 80.10 (8.44), 77.10 (9.26), 65.00 (9.86), 59.05 (3.99), 57.05 (0.72).

#### 3.2.8. Synthesis of N-(benzyloxy)-2-((4-(1,3-dioxoisoindolin-2-yl)phenyl)selanyl)acetamide (**13**)

We stirred 2-((4-Aminophenyl)selanyl)-N-(benzyloxy)acetamide (**8**) (335.27 mg, 1 mmol), phthalic anhydride (148 mg, 1 mmol), and 1 g of sodium acetate anhydrous under reflux in acetic acid (10 mL) for 6 h. After adding water, the mixture was neutralised with sodium carbonate. The resulting residue was filtered off and recrystallised from ethanol.

This resulted in formation of a purple solid, which yielded 248.7 mg (53%); Rf = 0.44 (chloroform: MeOH 5%); MP: 195–196 °C. I.R. (KBr) λ_max_. cm^−1^: 3145.51, 1701.88, 1654.62; ^1^H NMR (400 MHz, DMSO) δ 11.31 (s, 1H, NH), 7.95 (dd, *J* = 20.5, 2.7 Hz, 4H, Ar-H), 7.76–7.64 (m, 2H, Ar-H), 7.57–7.31 (m, 7H, Ar-H), 4.88 (s, 2H, CH_2_O), 3.51 (s, 2H, CH_2_Se); ^13^C NMR (101 MHz, DMSO) δ 167.39 (2 CO-N), 166.76, 136.31, 135.22, 132.34, 132.02, 131.22, 130.40, 129.37, 128.83, 128.42, 123.93, 77.31, 26.70; M.S. (E.I., 70 ev) *m*/*z* (%) = 466.25 (M^+^, 0.32), 302.15 (3.96), 170.90 (0.10), 107.15 (2.14), 91.10 (100.0, base peak), 80.05 (0.73), 77.05 (15.44), 76.05 (18.75), 65.05 (5.75), 59.05 (0.85), 57.05 (1.28).

#### 3.2.9. Synthesis of N-(benzyloxy)-2-((4-(2-chloroacetamido)phenyl)selanyl)acetamide (**14**)

Potassium carbonate (5 g, 36.17 mmol) was added to 2-((4-aminophenyl)selanyl)-*N*-(benzyloxy)acetamide (**8**) (670.54 mg, 2 mmol) in 30 mL of acetone. After cooling the reaction to 0 °C, chloroacetyl chloride (397.67 µL, 5 mmol) was added dropwise. The reaction was allowed to warm at room temperature and stirred overnight. The mixture was removed under reduced pressure and washed with water. The formed precipitate was collected to give a white solid, which yielded 651.4 mg (79%); Rf = 0.43 [DCM: MeOH 5%]; MP: 129–130 °C. I.R. (KBr) λ_max_. cm^−1^: 3202, 1652, 1539; ^1^H NMR (400 MHz, DMSO) δ 11.18 (s, 1H, NHO), 10.40 (s, 1H, NHCO), 7.45 (m, 4H, Ar-H), 7.37 (m, 4H, Ar-H), 4.73 (s, 2H, CH_2_O), 4.27 (s, 2H, CH_2_Se), 2.52 (s, 2H, CH_2_Cl); ^13^C NMR (101 MHz, DMSO) δ 166.81, 165.19, 138.40, 136.35, 133.82, 129.31, 128.80, 124.10, 120.47, 77.29, 44.04, 27.26. M.S. (E.I., 70 ev) *m*/*z* (%) = 412.20 (M^+^ + 1, 0.56), 262.10 (1.43), 106.15 (7.09), 92.10 (8.57), 91 (100.0, base peak), 80.10 (1.42), 77.10 (13.81), 65.05 (8.16), 59.05 (0.34), 57.10 (0.40).

#### 3.2.10. Synthesis of Ethyl 2-(2-(4-((2-((benzyloxy)amino)-2-oxoethyl)selanyl)phenyl)hydrazineylidene)-2-cyanoacetate (**15**)

We dissolved 2-((4-Aminophenyl)selanyl)-*N*-(benzyloxy)acetamide (**8**) (335.27 mg, 1 mmol) in a mixture of H_2_SO_4_ (1.5 mL) and distilled water (5 mL). The mixture was stirred at 0 °C in an ice bath. A cold solution of sodium nitrite (1.2 mmol, 82.8 mg in 1 mL of water) was added dropwise to produce the diazonium salt solution. The temperature of the solution was kept at 0 °C. Then, a solution of ethyl cyanoacetate (135.74 mg,1.2 mmol) and sodium acetate ((1 g), dissolved in 5 mL water) was added dropwise into the diazonium salt solution. The mixture was kept at 0 °C overnight. The resulting solution was neutralised with sodium carbonate to produce a yellow solid, which was washed with water. The product was isolated as a golden yellow solid, which yielded 189.4 mg (41%); Rf = 0.46 [Pet. ether: EtOAc (4:4)]; MP: 128–129 °C. I.R. (KBr) λ_max_. cm^−1^: 3218, 2220, 1713, 1599; ^1^HNMR (400 MHz, DMSO) δ 12.31 (s, 1H, NNH), 11.25 (s, 1H, NH), 7.59–7.55 (m, 2H, Ar-H), 7.41–7.37 (m, 7H, Ar-H), 4.75 (s, 2H, CH_2_O), 4.29 (q, *J* = 21.2, 7.1 Hz, 2H, CH_2_O), 3.39 (s, 2H, CH_2_Se), 1.31 (dt, *J* = 14.1, 7.1 Hz, 3H, CH_3_); ^13^C NMR (101 MHz, DMSO) δ 166.80, 161.06, 140.99, 136.36, 133.96, 129.29, 128.80, 128.76, 117.60, 117.41, 104.96, 77.28, 62.44, 61.68, 27.22, 14.70; M.S. (E.I., 70 ev) *m*/*z* (%) = 460.20 (M^+^ + 1, 3.51), 353.25 (1.70), 352.15 (7.48), 296.10 (4.87), 171.00 (0.69), 170.00 (1.19), 107.15 (7.21), 91 (100.0, base peak), 80.10 (1.55), 77.10 (14.70), 65.05 (5.61), 59.05 (1.73) 57.05 (0.73).

#### 3.2.11. Synthesis of N-(benzyloxy)-2-((4-((4-hydroxyphenyl)diazenyl)phenyl)selanyl)acetamide (**16**)

We dissolved 2-((4-aminophenyl)selanyl)-*N*-(benzyloxy)acetamide (**8**) (335.27 mg, 1 mmol) in distilled water (5 mL), and HCl (1.5 mL), and stirred at 0 °C in an ice bath. Then, a cold solution of sodium nitrite (164.5 mg, 2.2 mmol in 1 mL of water) was added dropwise into this solution while keeping the temperature between 0 and 5 °C. The formed diazonium salt solution was then added dropwise to a cooled mixture of phenol (112.93 mg, 1.2 mmol) and sodium hydroxide (10%, 5 mL). After this addition, the mixture was stirred for 2 h at 0 °C and then neutralised with sodium carbonate.

This resulted in the formation of a dark brown solid, which yielded 174 mg (40%); Rf = 0.43 [Pet. ether: EtOAc (4:4)]; MP: 142–143 °C. I.R. (KBr) λ_max_. cm^−1^: 3169, 1648, 1577; ^1^H NMR (400 MHz, DMSO) δ 11.42 (s, 1H, NH), 7.86–7.63 (m, 8H, Ar-H), 7.37 (m, 3H, Ar-H), 4.78 (s, 2H, CH_2_O), 3.78 (s, 2H, CH_2_Se); ^13^C NMR (101 MHz, DMSO) δ 166.62, 161.61, 151.44, 145.87, 136.33, 132.07, 131.54, 129.31, 128.80, 125.34, 123.15, 116.49, 77.31, 26.53; M.S. (E.I., 70 ev) *m*/*z* (%) = 441.20 (M^+^, 1.82), 277.20 (1.33), 171.00 (0.86), 121.15 (18.92), 107.15 (5.29), 93.10 (55.20), 91.10 (100.0, base peak), 80.10 (2.30), 77.10 (20.94), 76.15 (5.57), 65.05 (43.96), 59.05 (1.72), 57.05 (0.78).

### 3.3. Biological Assays

#### 3.3.1. Anticancer Activity

The products’ anticancer activity was examined using the MTT assay against breast (MCF-7) carcinoma cells, liver (HepG2), and normal WI-38 cells, following the previously reported method [38,44,45,46]. Experimental details can be found in the Supplementary Materials.

#### 3.3.2. Antimicrobial Activity

The products’ antimicrobial properties were examined according to the previously reported method, via agar well diffusion assay against *E. coli*, *S. aureus*, and *C. albicans* [45,46,47]. A microdilution method was also used to record MICs (M), following the previously reported procedure [29,45]. Experimental details can be found in the Supplementary Materials.

#### 3.3.3. Antioxidant Activity

The compounds’ antioxidant properties were assessed by in vitro bioassays using DPPH and ABTS, following the reported method [34,35,42,43,44]. Details are in the Supplementary Materials.

## 4. Conclusions

Novel hydroxamic acid-tethered OSe hybrids were synthesized in good yields, and their chemical structures were confirmed via spectroscopic methods. In addition, their biological activities were examined. Their antimicrobial and antitumor activities were examined against various microbial strains and cancer cells. OSe compound **8** showed promising anticancer activity with IC_50_ = 7.57 ± 0.5 µM against HepG2 and IC_50_ = 9.86 ± 0.7 µM against MCF-7 cells. Additionally, OSe compounds **8** and **15** exhibited good antimicrobial activities against *C. albicans* (IA% = 91.7 and 83.3) and *S. aureus* (IA% = 90.5 and 71.4).

Similarly, OSe compounds **8** and **16** showed 87 and 81.4% scavenging activities in the ABTS assay, compared to 88.4% by vitamin C. OSe compound **8** had 92.3% scavenging activity, compared to 94.5% by vitamin C in the DPPH assay. These results point to the potential antimicrobial, anticancer, and antioxidant properties of OSe compounds **8**, **13**, **15,** and **16,** and warrant further investigation of these hydroxamic acids’ derivatives.

While it is too early to evaluate why the OSe **8** was the most potential compound in most of the assays performed, one may guess that it might inhibit specific, biological target(s) and modify specific enzymes or proteins causing activation. Indeed, OSe **8** is amphiphilic, and therefore can enter cells without problems. Moreover, it is likely that OSe **8** may be altered in vivo into a metabolically active intermediate. It can also attack the cysteine proteins via its amino group, and this may upregulate the antioxidant pathways. Of course, these speculations require further in-depth research.

This pilot research has introduced simple synthetic avenues to develop tailor-made HA-based OSe compounds. Some of the synthesized HA-based OSe agents showed potential anticancer activities. We believe this provides ample scope for future study at the biology/chemistry interface. Future research might expand and/or refine the synthetic procedures proposed, building upon more and more diverse HA and OSe building blocks. Accordingly, the development of such HA-based OSe will be mainly driven by an interest in antimicrobial and anticancer drugs.

## Figures and Tables

**Table 1:** The antiproliferative activities of the OSe compounds.

Compounds	MCF7 ^a^		HepG2 ^a^		WI38 ^a^
	IC_50_ (µM) ^a^	TI ^c^	IC_50_ (µM) ^a^	TI ^c^	IC_50_ (µM) ^a^
Adriamycin	4.17 ± 0.2	1.6	4.50 ± 0.2	1.5	6.72 ± 0.5
**8**	9.86 ± 0.7	2.8	7.57 ± 0.5	3.6	27.42 ± 2.1
**9**	45.58 ± 2.9	1.9	53.04 ± 3.2	1.6	84.75 ± 4.8
**10**	49.29 ± 3.1	0.8	83.36 ± 4.2	0.5	37.39 ± 2.6
**11**	35.02 ± 2.5	1.5	48.78 ± 2.8	1.1	52.49 ± 3.2
**12**	63.02 ± 3.8	0.6	72.85 ± 3.7	0.5	36.53 ± 2.4
**13**	91.56 ± 4.6	0.8	>100 ^b^	0.6	61.57 ± 3.8
**14**	87.41 ± 4.1	1.1	>100 ^b^	1	>100 ^b^
**15**	21.58 ± 1.6	3.8	15.83 ± 1.3	5.2	82.03 ± 4.6
**16**	33.73 ± 2.4	1.9	26.48 ± 2.0	2.4	64.16 ± 3.9

^a^ Antitumor properties were estimated via MTT bioassay. MCF-7, HepG2, and WI-38 cells were incubated for 24 h with serial concentrations of the OSe compounds. ^b^ No proliferation inhibition was observed (IC_50_ ˃ 100 µM). ^c^ TI is defined as the ratio between the IC_50_ exhibited by the compound against WI38 cells to the compound’s respective IC_50_ against HepG2 and MCF-7 cells.

**Table 2:** The antimicrobial properties of the OSe compounds.

Compound	*E. coli*		*S. aureus*		*C. albicans*	
	ZID (mm) ^a^	IA% ^b^	ZID (mm) ^a^	IA% ^b^	ZID (mm) ^a^	IA% ^b^
**8**	16	69.6	19	90.5	22	91.7
**9**	6	26.1	7	33.3	9	37.5
**10**	NA	-	6	28.6	7	29.2
**11**	7	30.4	10	47.6	13	54.2
**12**	4	17.4	5	23.8	10	41.7
**13**	N.A.	-	NA	-	2	8.3
**14**	N.A.	-	NA	-	4	16.7
**15**	12	52.2	15	71.4	20	83.3
**16**	9	39.1	11	52.4	16	66.7
Ampicillin	23	100	21	100	-	-
Clotrimazole	-	-	-	-	24	100

^a^ Inhibition zones (ZID) are expressed as diameters (mm) employing 6-mm disks soaked with 20 μM of the OSe agents. ^b^ Activity index (IA%) percentage is the ratio of the inhibition zone of the OSe compound to the inhibition zone of the positive control.

**Table 3:** The MIC of OSe compound **8**.

Compounds	MIC (μM)		
	*E. coli*	*S. aureus*	*C. albicans*
**8**	8	4	4
Ampicillin	0.5	1	-
Clotrimazole	-	-	2

**Table 4:** OSe compound **8′s** redox properties.

Compounds	IC_50_ (µM)	
	DPPH	ABTS
Vitamin C	19.18 ± 0.13	28.16 ± 0.19
**8**	23.67 ± 0.21	27.24 ± 0.20

## Data Availability

Not applicable.

## Supplementary Materials

The following supporting information can be downloaded at: https://www.mdpi.com/article/10.3390/ph16030367/s1, Figure copies of ^1^H & ^13^CNMR spectra IR and MS. [42,47,48,49,50,51] are cited in the Supplementary Materials.
