# Supplementary material for: Design, Synthesis, and Biological Evaluation of Novel Hydroxamic Acid-Based Organoselenium Hybrids
Source: Pharmaceuticals (Basel). 2023 Feb 28;16(3):367. doi: 10.3390/ph16030367 (PMC10059083; doi:10.3390/ph16030367)
Supplement: Supplementary file 1 [file pharmaceuticals-16-00367-s001.zip › pharmaceuticals-2141764-supplementary.pdf]

Design, synthesis, and biological evaluation of novel hydroxamic acid-based organoselenium hybrids

Jameelah S. Alotaibi[1], Yasair S. Al-Faiyz[1], Saad Shaaban[1,2*]

[1]*Chemistry Department, College of Science, King Faisal University, Al-Ahsa 31982, Saudi Arabia.*

[2]*Chemistry Department, Faculty of Science, Mansoura University, Mansoura, Egypt.*

* Correspondence: sibrahim@kfu.edu.sa; dr_saad_chem@mans.edu.sa

SUPPORTING INFORMATION	PAGE
**The biological assays**	**S2**
**Copies of ^{1}H & ^{13}CNMR spectra IR and MS**	**S6**

1.1. The biological assays

1.1.1. The anticancer activity

The breast adenocarcinoma (MCF-7), Hepatocellular carcinoma (HEPG-2), and normal fibroblast (WI-38) cells were obtained from ATCC via Holding company for biological products and vaccines (VACSERA), Cairo, Egypt. The reagents RPMI-540 medium, MTT, DMSO, and fetal bovine serum was obtained from Sigma Aldrich. Dulbecco's modified Eagle's medium was used as the culture medium for the cells, and the cytotoxicity was evaluated using the reported MTT assay. Doxorubicin was used as the positive reference, and the IC_{50} values were obtained from the corresponding dose-response curve.

MTT assay[1-3]

This colorimetric assay is based on converting the yellow tetrazolium bromide (MTT) to a purple formazan derivative by mitochondrial succinate dehydrogenase in viable cells. Cell lines were cultured in RPMI-540 medium with 10% fetal bovine serum. Antibiotics added were 100 units/ml penicillin and 100µg/ml streptomycin at 37 C in a 5% Co_2 incubator. The cell lines were seeded in a 96-well plate at a density of $1.0x10^4$ cells/well. at 37 C for 48 h under 5% Co_2. After incubation, the cells were treated with different concentrations of compounds and incubated for 13 h. After 13 h of drug treatment, 9 µl of MTT solution at 5mg/ml was added and incubated for 4 h. Finally, dimethyl sulfoxide (DMSO) in the volume of 100 µl is added to each well to dissolve the purple formazan formed. The colorimetric assay is measured and recorded at an absorbance of 570 nm using a plate reader (EXL 800, USA). The percentage of the relative cell viability was calculated as (A570 of treated samples/A570 of the untreated sample) X 100.

1.1.2. The antimicrobial activity

The antimicrobial activities of the OSe compounds were evaluated against *C. albicans* yeast as well as *E. coli* gram-negative and *S. aureus* gram-positive bacteria employing the agar well diffusion assay [4].

Briefly, a concentration of 1 mM was prepared for each compound by dissolving in DMSO. Paper discs of standard size (5cm) were sterilized in an autoclave and soaked in 9 µL of the test compounds, and placed in the Petri dishes, which in turn contain a nutrient media (agar 9 g, peptone 5 g, and beef extract 3 g) seeded with the dedicated strain. Incubation lasted for 13 h at 36 ^{0}C. Experiments were replicated three times and the antifungal clotrimazole and antibiotic ampicillin were used as standards. The % activity index for the complex was determined and depicted in table 2.

Furthermore, the MICs (in µM) were determined by the microdilution method according to the reported protocol.

1.1.3. The antioxidant activity

1.1.3.1. The DPPH bioassay

The hydrogen atom or electron donation ability of the corresponding compounds was measured by estimating the bleaching of the purple color of a methanolic solution of DPPH [5]. This spectrophotometric assay uses stable DPPH$^{\cdot}$ reagent. The sample was prepared by adding 90 mL of the OSe compounds (1 mM in methanol) to 400 mL DPPH in methanol. After 30 min of incubation in the dark, the absorbance was read against a blank at 560 nm. Ascorbic acid (vitamin C) and ebselen were used as standard antioxidants (positive control). A blank sample was run without DPPH. A negative control sample was run using methanol instead of the sample. The radical scavenging activity was calculated

using the following equation:

$$\text{Inhibition\%} = (A_{blank} - A_{sample})/(A_{blank})*100.$$

1.1.3.2. The ABTS bioassay

The antioxidant activity of the investigated compounds was assessed using 2,2'-azino-bis(3-ethylbenzothiazoline-6-sulphonic acid (ABTS) method [6]. The radical cation derived from ABTS was

prepared by the reaction of 60 mM ABTS solution with 0.3 M manganese dioxide solution in 0.1M phosphate buffer, pH 7. Then, the mixture was shaken, centrifuged, filtered, and the absorbance ($A_{control}$) of the resulting green-blue solution (ABTS radical solution) was measured at wavelength 734 nm. Then, 50 mL of 1 mg/ml test compound in phosphate-buffered methanol was added. The absorbance (A_{test}) was measured. The reduction in color intensity was expressed as % inhibition. The % inhibition for each compound is calculated from the following equation

$$\text{Inhibition\%} = (A_{control}-A_{sample})/(A_{control})*100$$

Ascorbic acid (vitamin C) was used as a standard antioxidant (positive control). A blank sample was run without ABTS and using MeOH/phosphate buffer (1:1) instead of the sample. A negative control sample was run with MeOH/phosphate buffer (1:1) instead of a tested compound.

Synthesis of methyl (Z)-4-oxo-4-((4-selenocyanatophenyl)amino)but-2-enoate (**4**)

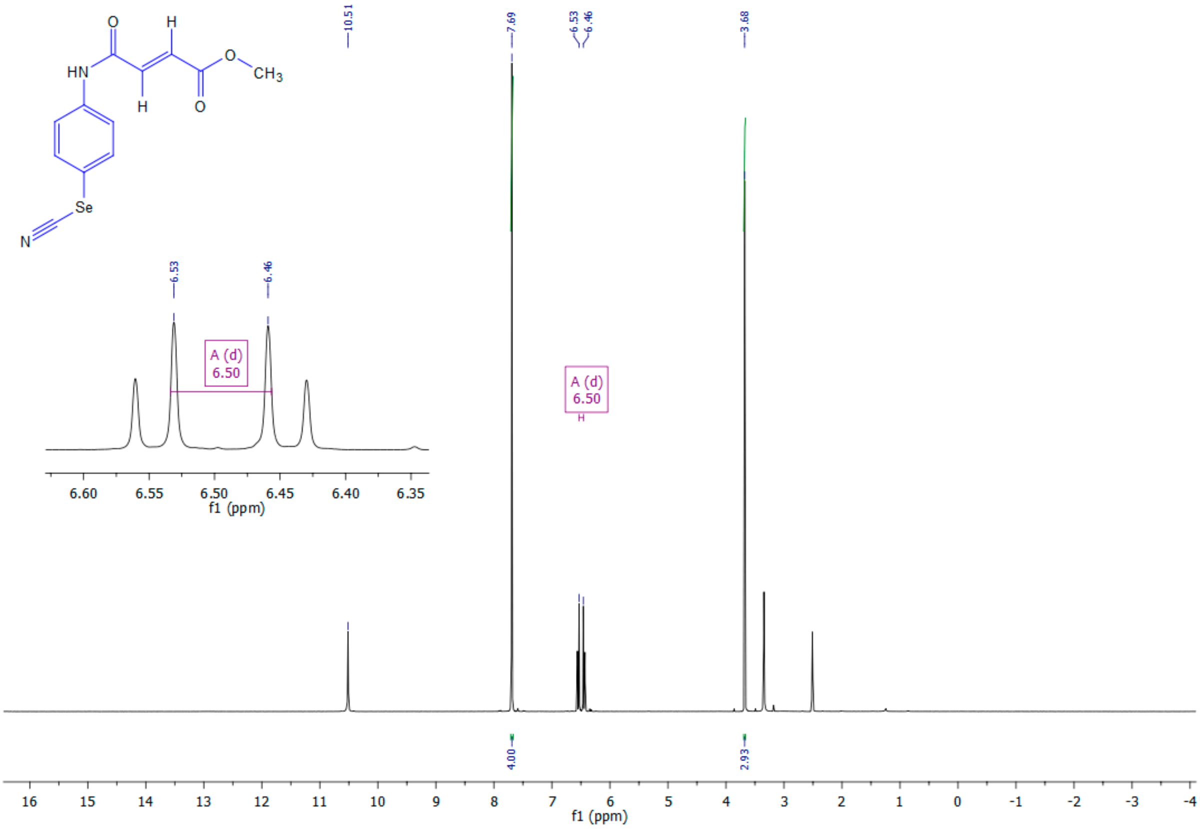

^{1}HNMR chart of compound **4**

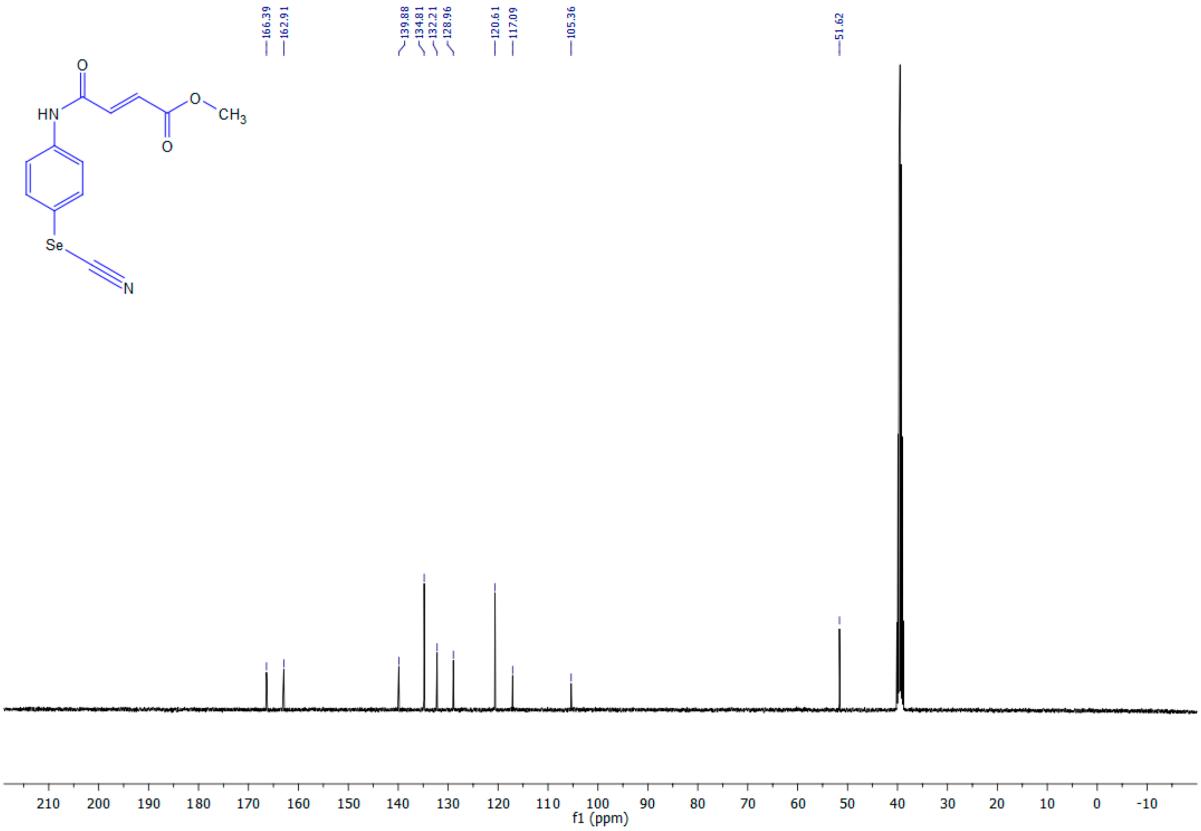

^{13}CNMR chart of compound **4**

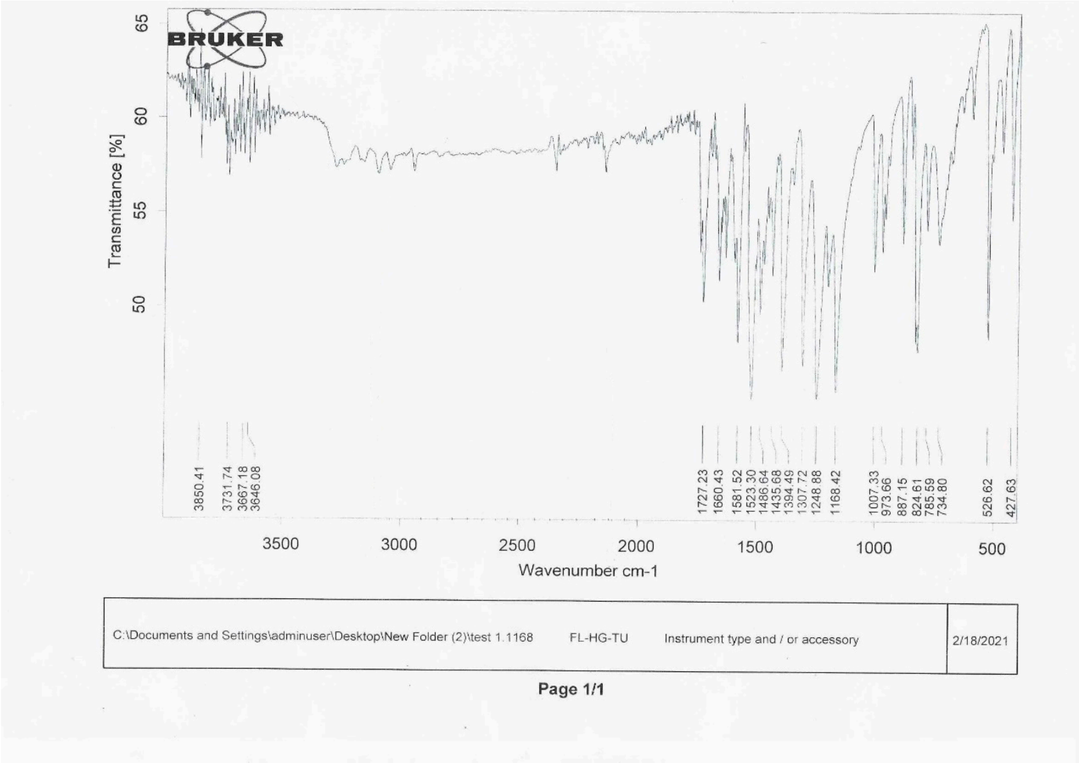

IR chart of compound **4**

Cairo University
Micro Analytical Center

DI Analysis
Shimadzu Qp-2010 Plus

Sample Information

Analyzed by	: Dr. Mai Younis
Analyzed	: 03/01/2007 08:51:36 م
Sample Name	: J1
Sample ID	:
Customer Name	: Dr.Mohamed Soliman - Science - Cairo
Data File	: C:\GCMSsolution\Data\Project1\J1.QGD
Org Data File	: C:\GCMSsolution\Data\Project1\J1.QGD
Method File	: C:\GCMSsolution\Data\Project1\High Temperature Op
Org Method File	: C:\GCMSsolution\Data\Project1\High Temperature Op
Report File	:
Tuning File	: C:\GCMSsolution\System\Tune1_default.qgt
$EndIf$Modified by	: Dr. Mai Younis
Modified	: 03/01/2007 08:57:09 م

Method

==== Analytical Line 1 ====

IonSourceTemp	:250.00 °C
[MS Table]	
--Group 1 - Event 1--	
Start Time	:0.00min
End Time	:10.00min
ACQ Mode	:Scan
Event Time	:0.50sec
Scan Speed	:1000
Start m/z	:50.00
End m/z	:510.00
Electron Voltage	: 70 eV
Ionization Mode	: EI

C:\GCMSsolution\Data\Project1\J1.QGD

Chromatogram J1 C:\GCMSsolution\Data\Project1\J1.QGD

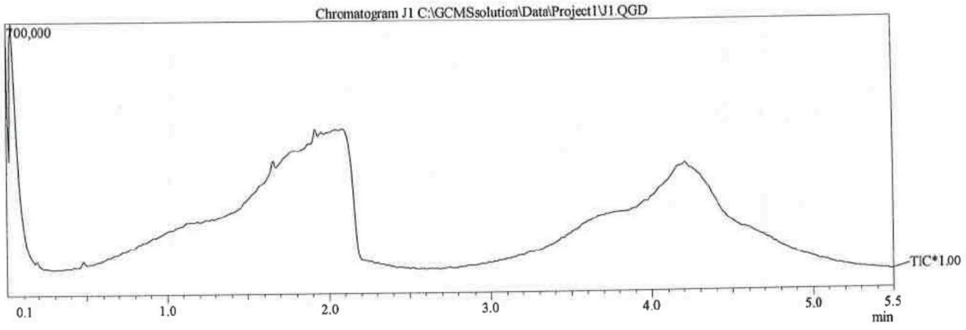

Spectrum

Line#:1 R.Time:2.0(Scan#:240)
MassPeaks:153
RawMode:Single 2.0(240) BasePeak:113(133438)
BG Mode:None Group 1 - Event 1

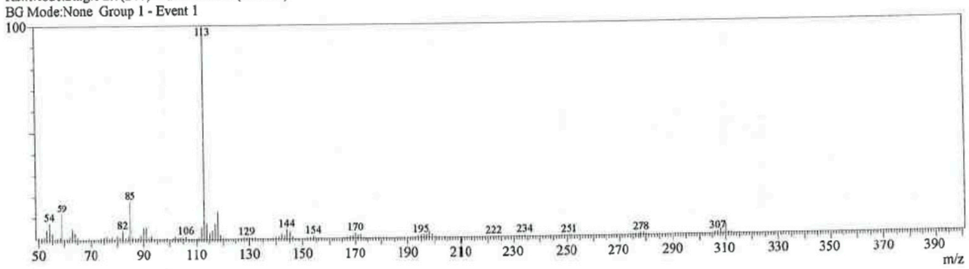

Mass Table
Line#:1 R.Time:2.0(Scan#:240)
MassPeaks:153
RawMode:Single 2.0(240) BasePeak:113(133438)
BG Mode:None Group 1 - Event 1

#	m/z	Abs. In	Rel. Int.	#	m/z	Abs. In	Rel. Int.	#	m/z	Abs. In	Rel. Int.
1	50.05	1593	1.19	4	53.00	5972	4.48	7	56.05	687	0.51
2	51.00	1282	0.96	5	54.00	10129	7.59	8	57.05	1217	0.91
3	52.05	1997	1.50	6	55.00	3809	2.85	9	58.05	1646	1.23

#	m/z	Abs. In	Rel. Int.	#	m/z	Abs. In	Rel. Int.	#	m/z	Abs. In	Rel. Int.
10	59.00	15969	11.97	58	107.05	607	0.45	106	174.00	294	0.22
11	60.00	737	0.55	59	108.10	354	0.27	107	179.00	231	0.17
12	61.05	850	0.64	60	109.10	270	0.20	108	181.00	303	0.23
13	62.05	1990	1.49	61	110.10	246	0.18	109	182.00	334	0.25
14	63.05	6812	5.10	62	111.15	1169	0.88	110	193.05	505	0.38
15	64.00	4215	3.16	63	112.15	7205	5.40	111	193.95	1133	0.85
16	65.00	1766	1.32	64	113.10	133438	100.00	112	195.00	1498	1.12
17	66.00	300	0.22	65	114.05	9392	7.04	113	196.00	1766	1.32
18	67.00	425	0.32	66	115.05	3720	2.79	114	197.05	2120	1.59
19	67.95	417	0.31	67	116.10	5118	3.84	115	198.05	3479	2.61
20	69.05	728	0.55	68	117.10	9236	6.92	116	199.05	1893	1.42
21	70.15	370	0.28	69	118.10	16642	12.47	117	200.00	705	0.53
22	71.10	572	0.43	70	119.05	2337	1.75	118	201.00	201	0.15
23	72.00	327	0.25	71	120.05	602	0.45	119	204.25	486	0.36
24	73.05	547	0.41	72	125.10	303	0.23	120	208.20	217	0.16
25	73.95	1081	0.81	73	126.00	294	0.22	121	222.20	263	0.20
26	75.05	1471	1.10	74	127.00	534	0.40	122	223.20	215	0.16
27	76.05	1933	1.45	75	128.05	909	0.68	123	224.20	306	0.23
28	77.05	790	0.59	76	129.05	992	0.74	124	230.20	218	0.16
29	78.00	1801	1.35	77	130.05	675	0.51	125	231.20	310	0.23
30	79.05	630	0.47	78	131.10	550	0.41	126	232.05	454	0.34
31	80.00	2382	1.79	79	132.10	753	0.56	127	233.05	416	0.31
32	81.05	1265	0.95	80	133.10	313	0.23	128	234.05	866	0.65
33	82.00	5273	3.95	81	135.10	244	0.18	129	235.10	201	0.15
34	83.10	1170	0.88	82	138.05	301	0.23	130	236.10	215	0.16
35	84.15	1921	1.44	83	139.05	602	0.45	131	248.10	319	0.24
36	85.05	23847	17.87	84	140.05	914	0.68	132	249.10	337	0.25
37	86.05	1449	1.09	85	141.05	1361	1.02	133	250.10	314	0.24
38	87.10	958	0.72	86	142.05	3143	2.36	134	250.95	406	0.30
39	88.15	1154	0.86	87	143.15	1966	1.47	135	252.05	488	0.37
40	89.10	2473	1.85	88	144.05	5600	4.20	136	274.10	308	0.23
41	90.10	6863	5.14	89	145.10	4322	3.24	137	275.05	385	0.29
42	91.05	7573	5.68	90	146.05	1295	0.97	138	276.10	783	0.59
43	92.05	1342	1.01	91	152.00	276	0.21	139	277.10	551	0.41
44	93.00	2266	1.70	92	153.05	630	0.47	140	278.10	1300	0.97
45	94.00	276	0.21	93	154.10	1449	1.09	141	279.10	1209	0.91
46	95.00	578	0.43	94	154.90	623	0.47	142	280.10	402	0.30
47	96.00	238	0.18	95	155.90	487	0.36	143	281.10	310	0.23
48	97.20	383	0.29	96	156.90	297	0.22	144	284.10	313	0.23
49	98.15	378	0.28	97	165.00	212	0.16	145	285.10	226	0.17
50	99.10	862	0.65	98	166.00	582	0.44	146	306.15	1105	0.83
51	100.05	330	0.25	99	167.00	921	0.69	147	307.10	1156	0.87
52	101.15	799	0.60	100	168.00	1546	1.16	148	308.10	2849	2.14
53	102.05	1604	1.20	101	169.05	1438	1.08	149	309.15	596	0.45
54	103.00	1105	0.83	102	170.00	3001	2.25	150	310.10	5922	4.44
55	104.00	1129	0.85	103	171.00	1598	1.20	151	311.05	1075	0.81
56	105.00	1108	0.83	104	172.00	2441	1.83	152	312.10	1116	0.84
57	106.00	1822	1.37	105	172.95	652	0.49	153	313.10	287	0.22

Mass chart of compound **4**

Synthesis of N-(benzyloxy)-2-chloroacetamide (7)

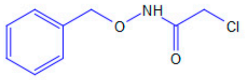

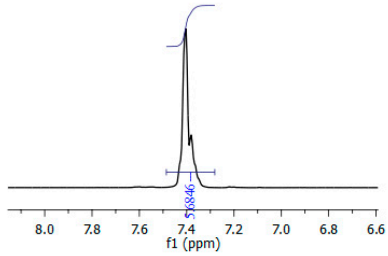

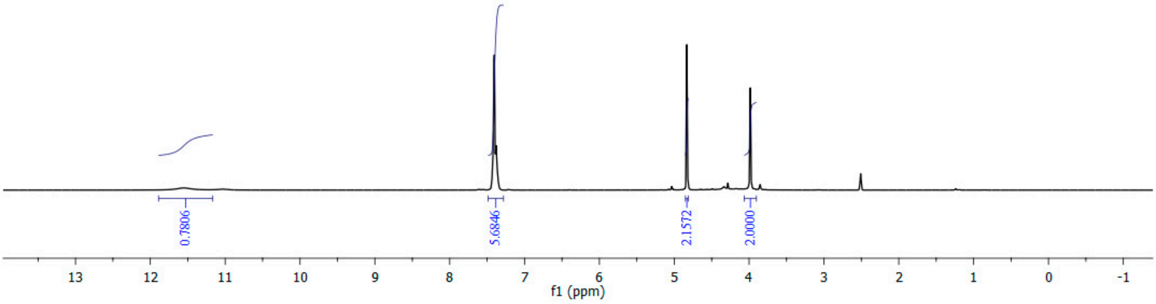

^{1}HNMR chart of compound **7**

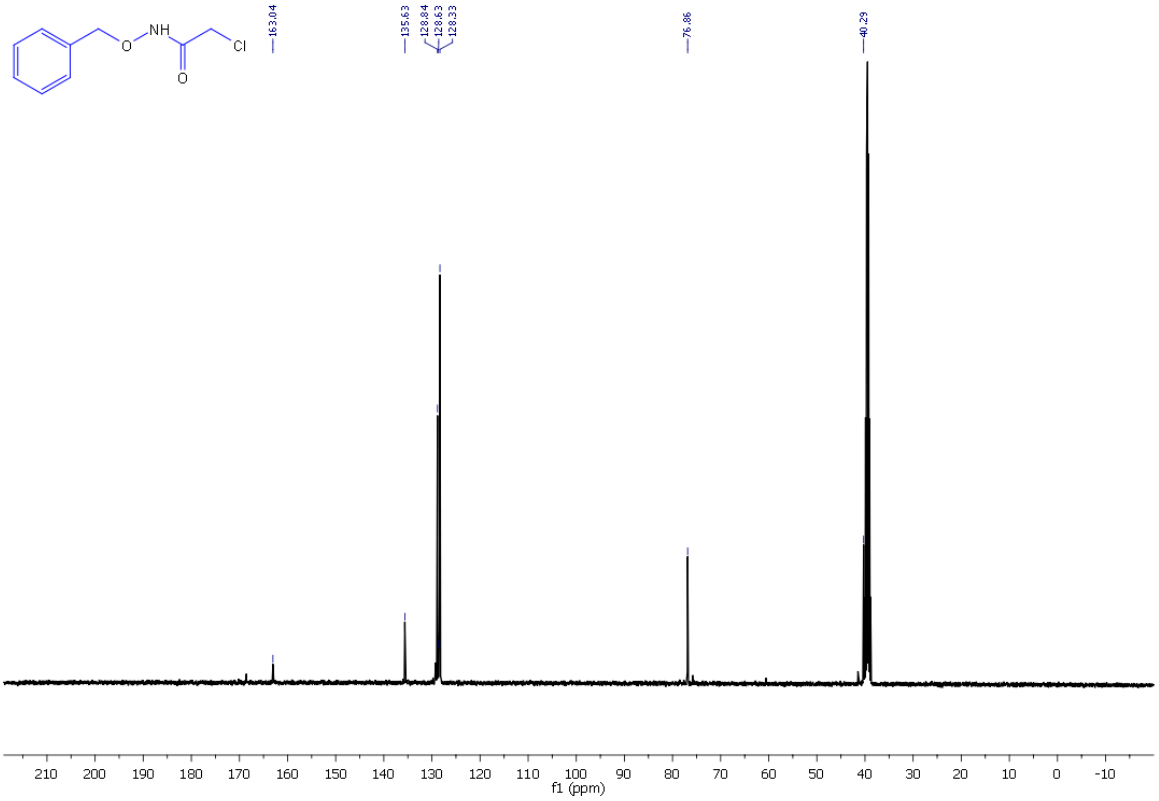

^{13}CNMR chart of compound **7**

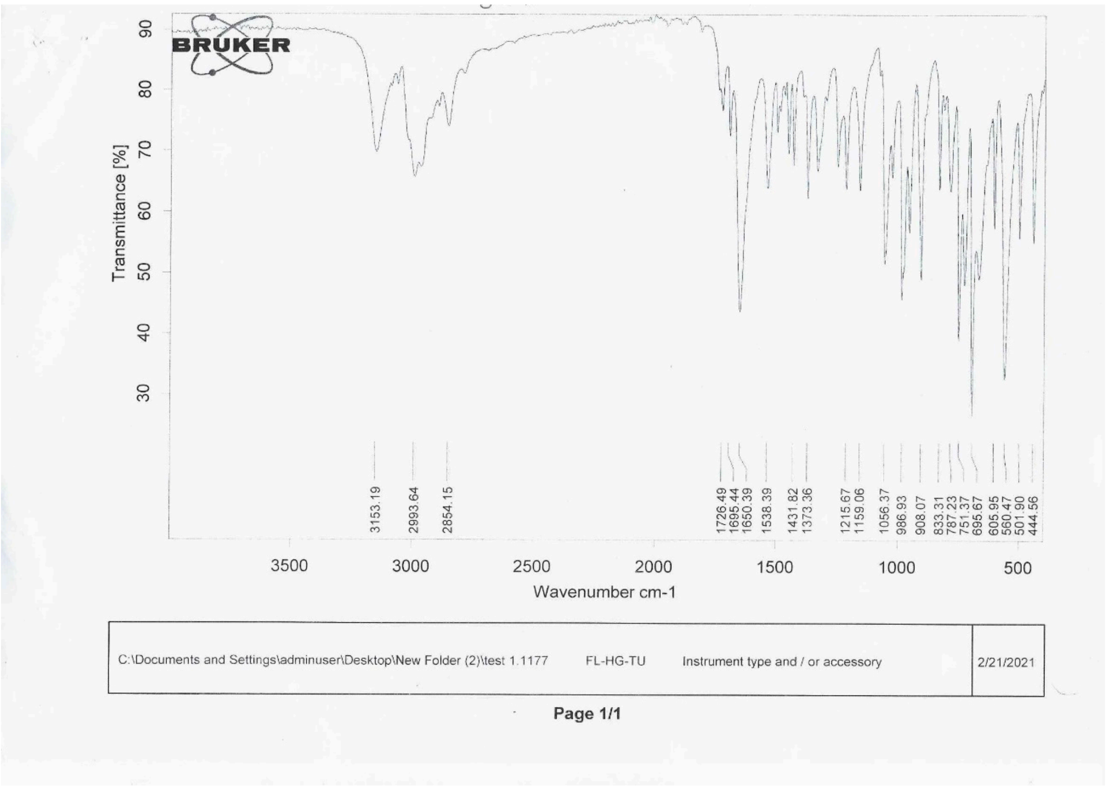

IR chart of compound **7**

03-Jan-07 20:52:07

Cairo University
Micro Analytical Center

DI Analysis
Shimadzu Qp-2010 Plus

Sample Information

Analyzed by	: Dr. Mai Younis
Analyzed	: 03/01/2007 08:42:53 م
Sample Name	: J3
Sample ID	:
Customer Name	: Dr.Mohamed Soliman - Science - Cairo
Data File	: C:\GCMSsolution\Data\Project1\J3.QGD
Org Data File	: C:\GCMSsolution\Data\Project1\J3.QGD
Method File	: C:\GCMSsolution\Data\Project1\High Temperature Op
Org Method File	: C:\GCMSsolution\Data\Project1\High Temperature Op
Report File	:
Tuning File	: C:\GCMSsolution\System\Tune1_default.qgt
$EndIf$Modified by	: Dr. Mai Younis
Modified	: 03/01/2007 08:48:35 م

Method

===== Analytical Line 1 =====

IonSourceTemp	:250.00 °C
[MS Table]	
--Group 1 - Event 1--	
Start Time	:0.00min
End Time	:10.00min
ACQ Mode	:Scan
Event Time	:0.50sec
Scan Speed	:1000
Start m/z	:50.00
End m/z	:510.00
Electron Voltage	: 70 eV
Ionization Mode	: EI

C:\GCMSsolution\Data\Project1\J3.QGD

Chromatogram J3 C:\GCMSsolution\Data\Project1\J3.QGD

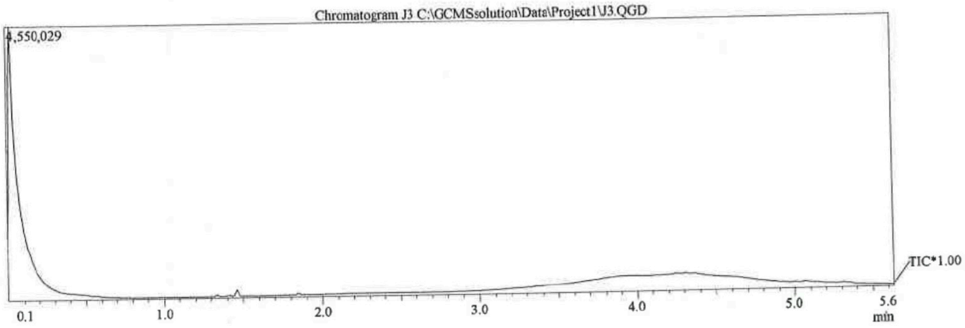

Spectrum

Line#:1 R.Time:4.3(Scan#:512)
MassPeaks:153
RawMode:Single 4.3(512) BasePeak:59(65519)
BG Mode:None Group 1 - Event 1

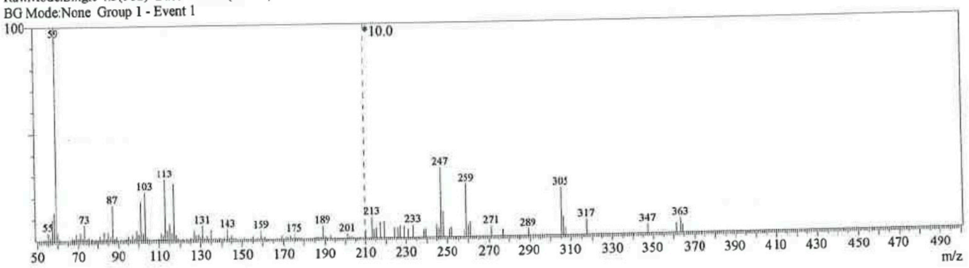

Mass Table
Line#:1 R.Time:4.3(Scan#:512)
MassPeaks:153
RawMode:Single 4.3(512) BasePeak:59(65519)
BG Mode:None Group 1 - Event 1

#	m/z	Abs. Int	Rel. Int.	#	m/z	Abs. Int	Rel. Int.	#	m/z	Abs. Int	Rel. Int.
1	53.00	348	0.53	4	56.05	1156	1.76	7	59.05	65519	100.00
2	53.95	403	0.62	5	57.05	6546	9.99	8	60.00	2690	4.11
3	55.05	2596	3.96	6	58.05	8638	13.18	9	61.05	657	1.00

#	m/z	Abs. In	Rel. Int.
10	67.05	711	1.09
11	68.05	522	0.80
12	69.05	2365	3.61
13	70.10	977	1.49
14	71.05	2607	3.98
15	72.05	959	1.46
16	73.05	4814	7.35
17	74.05	598	0.91
18	75.05	1157	1.77
19	77.05	602	0.92
20	79.10	321	0.49
21	80.10	265	0.40
22	81.05	1455	2.22
23	82.15	523	0.80
24	83.10	2743	4.19
25	84.15	845	1.29
26	85.05	2722	4.15
27	86.15	1354	2.07
28	87.10	10878	16.60
29	88.05	731	1.12
30	89.05	1478	2.26
31	91.15	410	0.63
32	93.25	385	0.59
33	94.15	375	0.57
34	95.15	1540	2.35
35	96.15	636	0.97
36	97.10	1817	2.77
37	98.15	866	1.32
38	99.15	2961	4.52
39	100.15	1932	2.95
40	101.10	12030	18.36
41	102.15	2166	3.31
42	103.10	14703	22.44
43	104.10	1009	1.54
44	105.10	391	0.60
45	107.10	305	0.47
46	108.20	212	0.32
47	109.15	598	0.91
48	110.15	475	0.72
49	111.10	2210	3.37
50	112.15	1595	2.43
51	113.10	18516	28.26
52	114.15	3014	4.60
53	115.15	5055	7.72
54	116.15	2239	3.42
55	117.15	17215	26.27
56	118.20	1753	2.68
57	119.15	810	1.24
58	121.10	356	0.54
59	123.10	460	0.70
60	125.10	647	0.99
61	126.15	638	0.97
62	127.15	2971	4.53
63	128.15	1431	2.18
64	129.15	1760	2.69
65	130.15	778	1.19
66	131.15	4233	6.46
67	132.15	618	0.94
68	133.20	1233	1.88
69	134.25	462	0.71
70	135.15	3046	4.65
71	136.20	358	0.55
72	137.20	298	0.45
73	139.10	641	0.98
74	140.10	302	0.46
75	141.10	759	1.16
76	142.15	312	0.48
77	143.10	3119	4.76
78	144.15	579	0.88
79	145.10	1303	1.99
80	146.15	522	0.80
81	147.10	537	0.82
82	151.10	694	1.06
83	152.10	270	0.41
84	153.10	314	0.48
85	154.10	330	0.50
86	155.10	929	1.42
87	157.15	521	0.80
88	158.15	307	0.47
89	159.10	2626	4.01
90	160.05	499	0.76
91	161.00	743	1.13
92	166.00	212	0.32
93	167.00	311	0.47
94	169.15	1046	1.60
95	170.20	214	0.33
96	171.15	742	1.13
97	172.15	491	0.75
98	173.15	985	1.50
99	174.25	366	0.56
100	175.15	1449	2.21
101	176.15	347	0.53
102	177.20	385	0.59
103	177.80	276	0.42
104	178.80	340	0.52
105	185.15	377	0.58
106	186.10	486	0.74
107	187.05	471	0.72
108	188.25	412	0.63
109	189.15	3802	5.80
110	190.15	605	0.92
111	191.20	274	0.42
112	193.15	935	1.43
113	195.20	209	0.32
114	201.15	1311	2.00
115	202.20	409	0.62
116	203.20	546	0.83
117	204.20	210	0.32
118	207.20	220	0.34
119	210.20	233	0.36
120	213.30	615	0.94
121	214.30	233	0.36
122	215.30	297	0.45
123	217.20	462	0.71
124	219.20	487	0.74
125	224.20	295	0.45
126	226.20	287	0.44
127	227.20	356	0.54
128	229.20	329	0.50
129	231.20	247	0.38
130	233.40	367	0.56
131	238.40	217	0.33
132	239.40	260	0.40
133	245.10	375	0.57
134	246.20	260	0.40
135	247.25	2116	3.23
136	248.25	754	1.15
137	251.20	241	0.37
138	252.20	294	0.45
139	258.30	212	0.32
140	259.25	1589	2.43
141	260.30	350	0.53
142	261.10	444	0.68
143	271.10	302	0.46
144	277.10	209	0.32
145	289.10	201	0.31
146	305.30	1431	2.18
147	306.25	547	0.83
148	307.30	214	0.33
149	317.40	449	0.69
150	347.40	263	0.40
151	361.40	284	0.43
152	363.35	436	0.67
153	364.40	246	0.38

Mass chart of compound **7**

*Synthesis of 2-((4-aminophenyl)selanyl)-N-(benzyloxy)acetamide (**8**)*

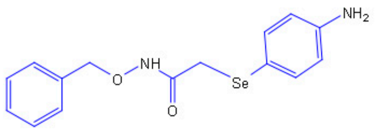

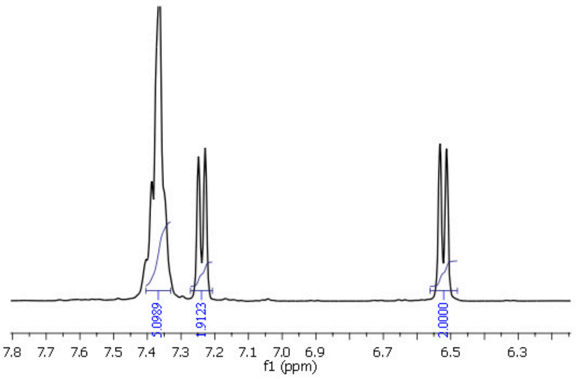

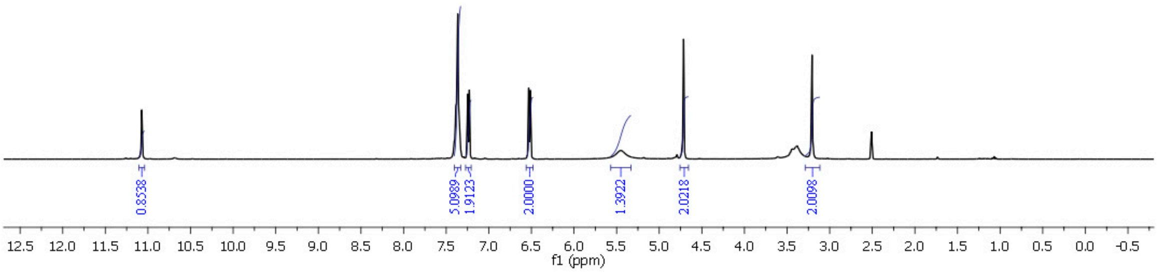

^{1}HNMR chart of compound **8**

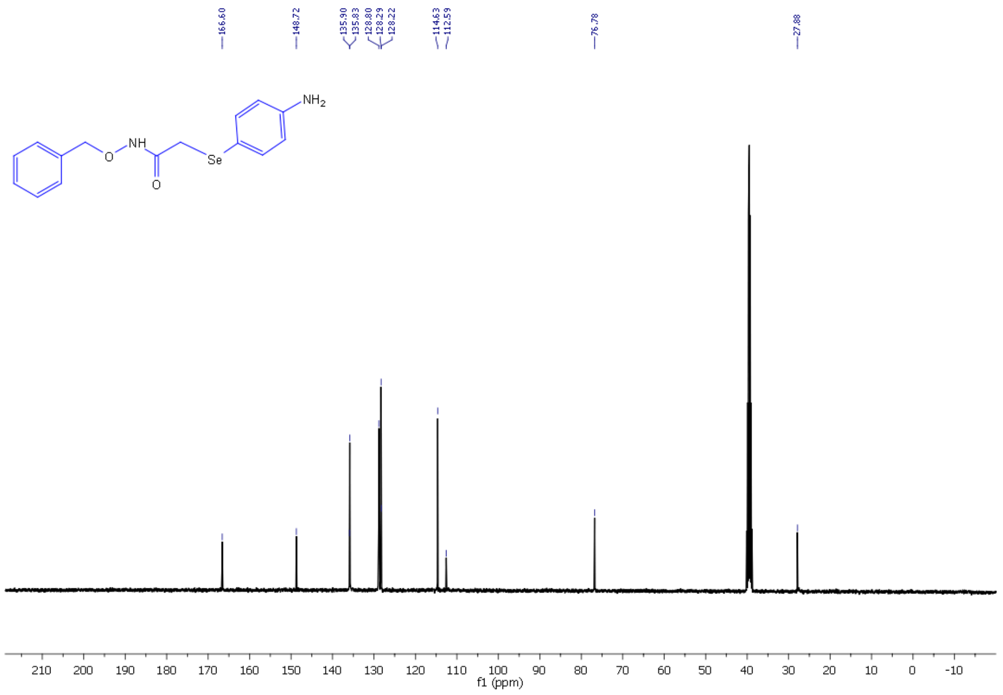

^{13}CNMR chart of compound **8**

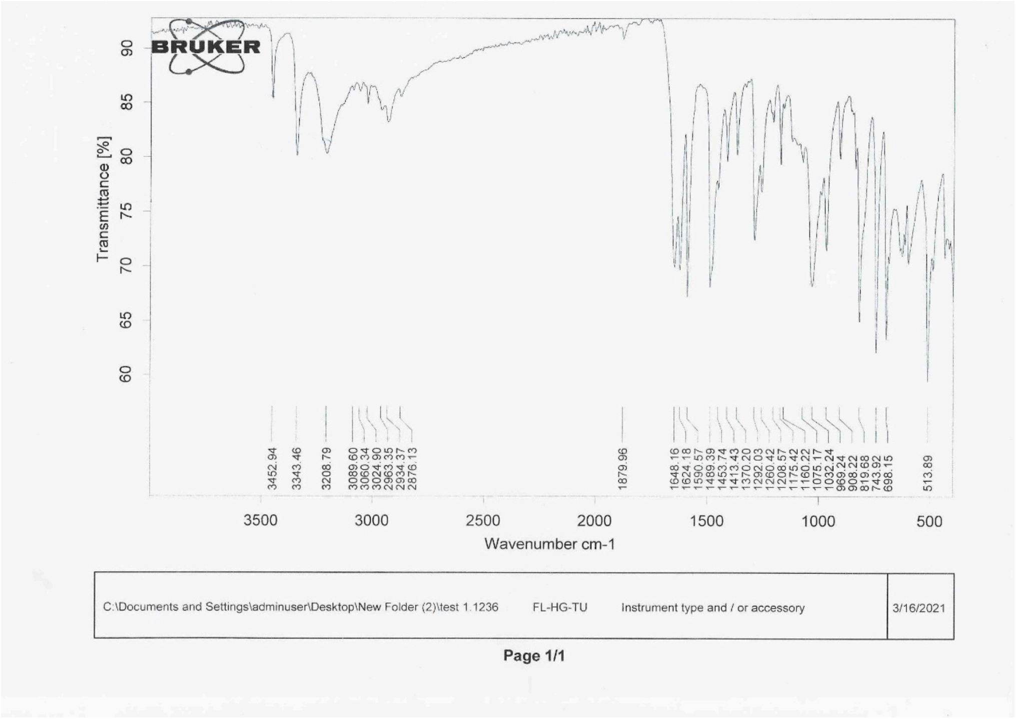

IR chart of compound **8**

Cairo University
Micro Analytical Center

DI Analysis
Shimadzu Qp-2010 Plus

Sample Information

Analyzed by	: Dr. Mai Younis
Analyzed	: 06/01/2007 05:50:43 م
Sample Name	: J5
Sample ID	:
Customer Name	: Dr.Mohamed Soliman - Science - Cairo
Data File	: C:\GCMSsolution\Data\Project1\J5.QGD
Org Data File	: C:\GCMSsolution\Data\Project1\J5.QGD
Method File	: C:\GCMSsolution\Data\Project1\High Temperature Op
Org Method File	: C:\GCMSsolution\Data\Project1\High Temperature Op
Report File	:
Tuning File	: C:\GCMSsolution\System\Tune1_default.qgt
$EndIf$Modified by	: Dr. Mai Younis
Modified	: 06/01/2007 05:54:37 م

Method

==== Analytical Line 1 ====

IonSourceTemp	:250.00 °C
[MS Table]	
--Group 1 - Event 1--	
Start Time	:0.00min
End Time	:10.00min
ACQ Mode	:Scan
Event Time	:0.50sec
Scan Speed	:1000
Start m/z	:50.00
End m/z	:510.00
Electron Voltage	:70 eV
Ionization Mode	: EI

C:\GCMSsolution\Data\Project1\J5.QGD

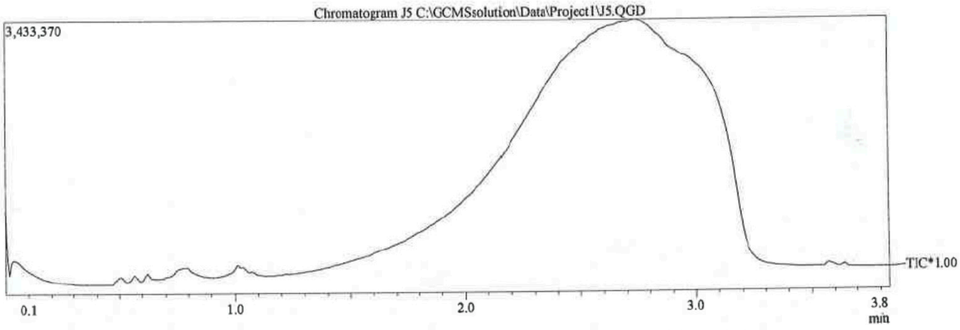

Spectrum

Line#:1 R.Time:2.4(Scan#:285)
MassPeaks:182
RawMode:Single 2.4(285) BasePeak:91(960460)
BG Mode:None Group 1 - Event 1

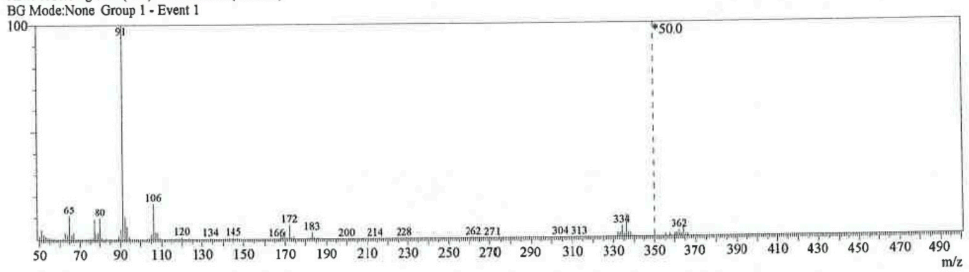

Mass Table
Line#:1 R.Time:2.4(Scan#:285)
MassPeaks:182
RawMode:Single 2.4(285) BasePeak:91(960460)
BG Mode:None Group 1 - Event 1

#	m/z	Abs. Int	Rel. Int.	#	m/z	Abs. Int	Rel. Int.	#	m/z	Abs. Int	Rel. Int.
1	50.05	15768	1.64	4	53.05	15780	1.64	7	56.05	4677	0.49
2	51.05	43235	4.50	5	54.05	9148	0.95	8	57.05	7827	0.81
3	52.05	21915	2.28	6	55.05	8837	0.92	9	58.10	1159	0.12

#	m/z	Abs. In	Rel. Int.
10	59.05	3823	0.40
11	60.05	3004	0.31
12	61.10	2431	0.25
13	62.10	7174	0.75
14	63.05	32170	3.35
15	64.15	21067	2.19
16	65.05	106060	11.04
17	66.10	17980	1.87
18	67.05	30605	3.19
19	68.10	2798	0.29
20	69.10	5318	0.55
21	70.10	2114	0.22
22	71.15	4248	0.44
23	72.15	765	0.08
24	73.05	3984	0.41
25	74.05	5212	0.54
26	75.10	4412	0.46
27	76.15	8246	0.86
28	77.10	88946	9.26
29	78.10	24337	2.53
30	79.15	30610	3.19
31	80.10	95319	9.92
32	81.10	8633	0.90
33	82.15	2120	0.22
34	83.15	5500	0.57
35	84.15	2638	0.27
36	85.15	3189	0.33
37	86.10	1175	0.12
38	87.10	1695	0.18
39	88.15	1354	0.14
40	89.10	15700	1.63
41	90.15	44615	4.65
42	91.10	960460	100.00
43	92.10	98228	10.23
44	93.10	56674	5.90
45	94.10	11602	1.21
46	95.10	4519	0.47
47	96.15	2320	0.24
48	97.15	3774	0.39
49	98.15	2232	0.23
50	99.15	1190	0.12
51	100.15	506	0.05
52	101.15	929	0.10
53	102.10	990	0.10
54	103.15	1634	0.17
55	104.15	7689	0.80
56	105.15	24236	2.52
57	106.15	160312	16.69
58	107.15	32112	3.34
59	108.10	29788	3.10
60	109.15	6176	0.64
61	110.15	1323	0.14
62	111.15	1816	0.19
63	112.15	1175	0.12
64	113.10	1337	0.14
65	114.10	1284	0.13
66	115.05	2947	0.31
67	116.05	1880	0.20
68	117.05	4301	0.45
69	118.05	2899	0.30
70	119.10	2788	0.29
71	120.10	10220	1.06
72	121.15	2539	0.26
73	122.15	937	0.10
74	123.15	4636	0.48
75	124.15	1292	0.13
76	125.15	799	0.08
77	126.10	564	0.06
78	127.10	922	0.10
79	128.05	703	0.07
80	129.10	1506	0.16
81	130.10	862	0.09
82	131.05	786	0.08
83	132.10	911	0.09
84	133.15	1465	0.15
85	134.15	4315	0.45
86	135.10	1537	0.16
87	136.05	482	0.05
88	137.15	486	0.05
89	138.05	375	0.04
90	139.05	677	0.07
91	140.05	723	0.08
92	141.05	2098	0.22
93	142.05	2068	0.22
94	143.05	3399	0.35
95	144.05	2313	0.24
96	145.00	5676	0.59
97	146.05	875	0.09
98	147.00	1714	0.18
99	148.10	628	0.07
100	149.10	572	0.06
101	150.10	220	0.02
102	151.10	303	0.03
103	152.00	396	0.04
104	153.00	447	0.05
105	153.95	404	0.04
106	155.00	348	0.04
107	155.90	390	0.04
108	156.90	402	0.04
109	158.00	492	0.05
110	159.00	238	0.02
111	160.00	217	0.02
112	165.05	553	0.06
113	166.00	1567	0.16
114	167.05	1160	0.12
115	168.05	13576	1.41
116	169.05	13380	1.39
117	170.00	31930	3.32
118	171.05	5744	0.60
119	172.05	61287	6.38
120	172.95	5359	0.56
121	174.05	10628	1.11
122	174.95	915	0.10
123	180.20	590	0.06
124	181.25	654	0.07
125	182.25	5051	0.53
126	183.15	28298	2.95
127	184.15	7005	0.73
128	185.05	1633	0.17
129	186.00	5919	0.62
130	187.10	1247	0.13
131	188.05	1063	0.11
132	200.10	298	0.03
133	210.10	201	0.02
134	212.10	222	0.02
135	213.10	298	0.03
136	214.10	382	0.04
137	224.10	233	0.02
138	225.10	255	0.03
139	226.10	447	0.05
140	227.10	241	0.03
141	228.10	951	0.10
142	229.10	358	0.04
143	230.15	747	0.08
144	258.10	623	0.06
145	259.15	627	0.07
146	260.10	1359	0.14
147	261.10	826	0.09
148	262.10	2494	0.26
149	263.10	1543	0.16
150	264.05	821	0.09
151	265.20	462	0.05
152	270.20	335	0.03
153	271.30	486	0.05
154	295.30	239	0.02
155	302.30	242	0.03
156	304.20	569	0.06
157	313.45	508	0.05
158	319.40	210	0.02
159	321.40	375	0.04
160	330.20	1868	0.19
161	331.25	952	0.10
162	332.20	18135	1.89
163	333.20	18686	1.95
164	334.20	47150	4.91
165	335.25	12104	1.26
166	336.20	96114	10.01
167	337.15	18937	1.97
168	338.20	19132	1.99
169	339.15	3752	0.39
170	340.15	624	0.06
171	341.25	362	0.04
172	342.20	281	0.03
173	344.20	388	0.04
174	355.20	238	0.02
175	357.20	249	0.03
176	360.20	282	0.03
177	361.20	292	0.03
178	362.20	513	0.05
179	363.20	212	0.02
180	364.20	870	0.09
181	365.20	207	0.02
182	366.20	242	0.03

Mass chart of compound **8**

*Synthesis of 2-((4-acetamidophenyl)selanyl)-N-(benzyloxy)acetamide (**9**)*

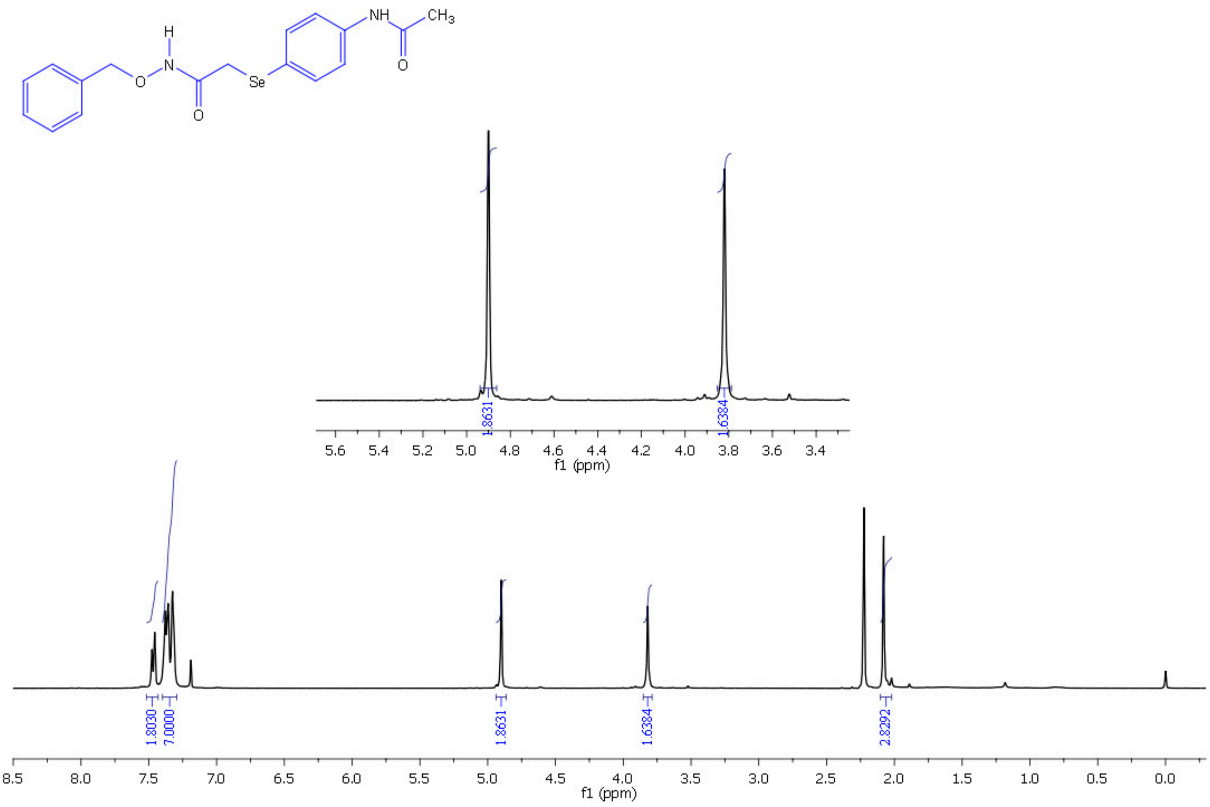

^{1}HNMR chart of compound **9**

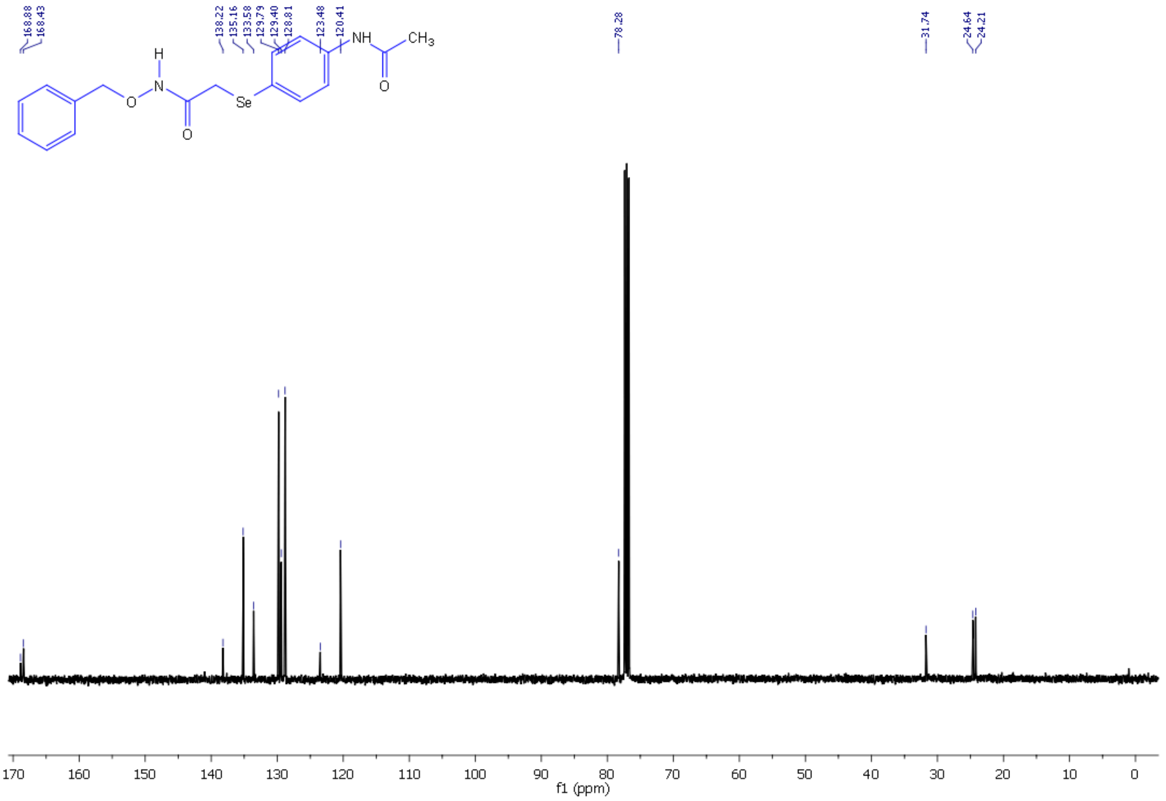

^{13}CNMR chart of compound **9**

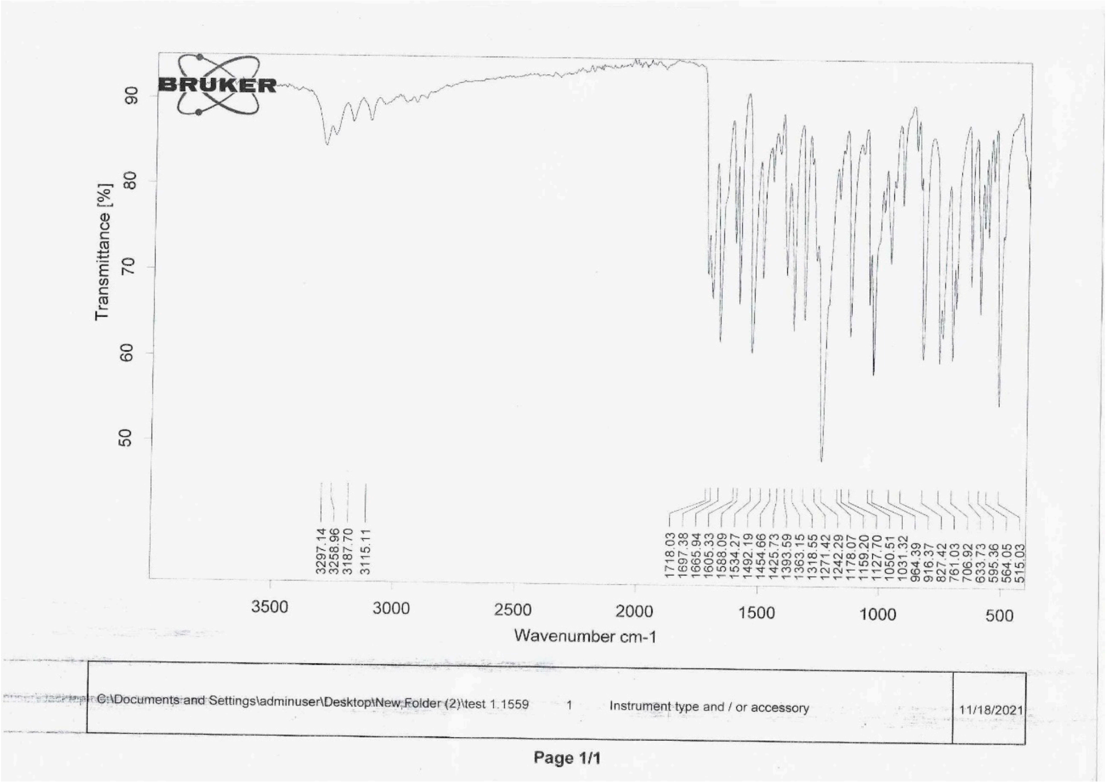

IR chart of compound **9**

Cairo University
Micro Analytical Center

DI Analysis
Shimadzu Qp-2010 Plus

Sample Information

Analyzed by : Dr. Mai Younis
Analyzed : 06/01/2007 06:02:32 م
Sample Name : J7
Sample ID :
Customer Name : Dr.Mohamed Soliman - Science - Cairo
Data File : C:\GCMSsolution\Data\Project1\J7.QGD
Org Data File : C:\GCMSsolution\Data\Project1\J7.QGD
Method File : C:\GCMSsolution\Data\Project1\High Temperature Op
Org Method File : C:\GCMSsolution\Data\Project1\High Temperature Op
Report File :
Tuning File : C:\GCMSsolution\System\Tune1_default.qgt
$EndIf$Modified by : Dr. Mai Younis
Modified : 06/01/2007 06:08:09 م

Method

===== Analytical Line 1 =====
IonSourceTemp :250.00 °C
[MS Table]
--Group 1 - Event 1--
Start Time :0.00min
End Time :10.00min
ACQ Mode :Scan
Event Time :0.50sec
Scan Speed :1000
Start m/z :50.00
End m/z :510.00

Electron Voltage : 70 eV
Ionization Mode : EI

C:\GCMSsolution\Data\Project1\J7.QGD

Chromatogram J7 C:\GCMSsolution\Data\Project1\J7.QGD

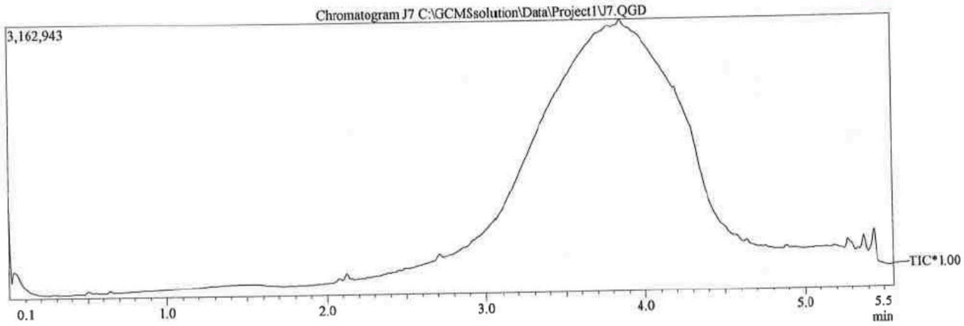

Spectrum

Line#:1 R.Time:3.6(Scan#:434)
MassPeaks:264
RawMode:Single 3.6(434) BasePeak:91(1210535)
BG Mode:None Group 1 - Event 1

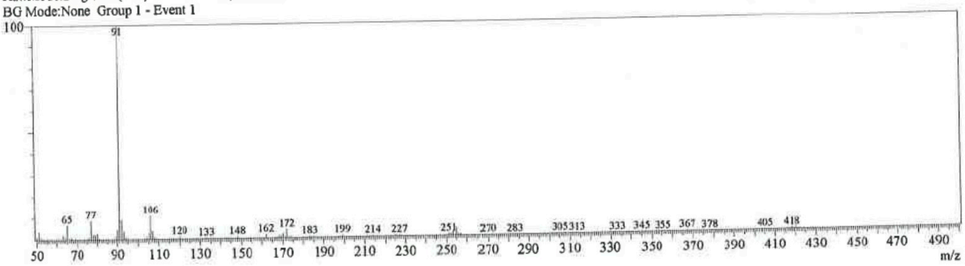

Mass Table
Line#:1 R.Time:3.6(Scan#:434)
MassPeaks:264
RawMode:Single 3.6(434) BasePeak:91(1210535)
BG Mode:None Group 1 - Event 1

#	m/z	Abs. In	Rel. Int.	#	m/z	Abs. In	Rel. Int.	#	m/z	Abs. In	Rel. Int.
1	50.05	15477	1.28	4	53.05	7183	0.59	7	56.10	3204	0.26
2	51.05	45107	3.73	5	54.05	3209	0.27	8	57.10	8273	0.68
3	52.05	13698	1.13	6	55.05	7711	0.64	9	58.05	1463	0.12

#	m/z	Abs. Int	Rel. Int.
10	59.05	1648	0.14
11	60.00	3386	0.28
12	61.05	1703	0.14
13	62.10	5530	0.46
14	63.05	26216	2.17
15	64.15	13903	1.15
16	65.05	81929	6.77
17	66.10	8124	0.67
18	67.10	11962	0.99
19	68.10	2002	0.17
20	69.10	5015	0.41
21	70.10	2759	0.23
22	71.10	4161	0.34
23	72.15	679	0.06
24	73.10	2354	0.19
25	74.05	5066	0.42
26	75.10	4806	0.40
27	76.15	10571	0.87
28	77.10	106641	8.81
29	78.10	26940	2.23
30	79.10	25310	2.09
31	80.10	35786	2.96
32	81.10	5642	0.47
33	82.15	1861	0.15
34	83.15	4489	0.37
35	84.15	2733	0.23
36	85.15	3762	0.31
37	86.15	1819	0.15
38	87.10	1862	0.15
39	88.15	2303	0.19
40	89.10	18366	1.52
41	90.15	53112	4.39
42	91.10	121053	100.00
43	92.10	107703	8.90
44	93.10	42689	3.53
45	94.10	8558	0.71
46	95.10	4926	0.41
47	96.20	1993	0.16
48	97.15	3817	0.32
49	98.15	3390	0.28
50	99.15	1680	0.14
51	100.10	3327	0.27
52	101.10	2022	0.17
53	102.15	1829	0.15
54	103.15	3258	0.27
55	104.15	10203	0.84
56	105.15	32274	2.67
57	106.15	132039	10.91
58	107.15	42954	3.55
59	108.10	9614	0.79
60	109.15	3215	0.27
61	110.20	1223	0.10
62	111.20	1921	0.16
63	112.15	1332	0.11
64	113.15	1333	0.11
65	114.10	1834	0.15
66	115.10	3858	0.32
67	116.10	3359	0.28
68	117.05	4485	0.37
69	118.10	4479	0.37
70	119.15	3593	0.30
71	120.10	14065	1.16
72	121.15	2792	0.23
73	122.20	954	0.08
74	123.20	2657	0.22
75	124.20	1007	0.08
76	125.20	844	0.07
77	126.15	784	0.06
78	127.15	1405	0.12

#	m/z	Abs. Int	Rel. Int.
79	128.15	2296	0.19
80	129.15	1947	0.16
81	130.10	1274	0.11
82	131.20	1105	0.09
83	132.15	3370	0.28
84	133.15	4647	0.38
85	134.15	2904	0.24
86	135.15	2204	0.18
87	136.15	1953	0.16
88	137.15	871	0.07
89	138.05	415	0.03
90	139.05	1003	0.08
91	140.05	1472	0.12
92	141.05	2462	0.20
93	142.05	2465	0.20
94	143.05	3038	0.25
95	144.05	3335	0.28
96	145.05	3859	0.32
97	146.10	1494	0.12
98	147.15	2016	0.17
99	148.15	9010	0.74
100	149.15	1723	0.14
101	150.15	1046	0.09
102	151.10	657	0.05
103	152.15	595	0.05
104	153.05	787	0.07
105	154.10	762	0.06
106	155.10	2196	0.18
107	156.10	2415	0.20
108	157.10	914	0.08
109	158.00	650	0.05
110	159.00	378	0.03
111	161.15	923	0.08
112	162.10	20801	1.72
113	163.10	2178	0.18
114	164.05	538	0.04
115	165.05	997	0.08
116	166.05	2864	0.24
117	167.05	3483	0.29
118	168.05	10991	0.91
119	169.05	10983	0.91
120	170.05	24761	2.05
121	171.05	10754	0.89
122	172.05	46264	3.82
123	173.00	5638	0.47
124	174.05	8057	0.67
125	175.10	1546	0.13
126	176.10	407	0.03
127	180.15	742	0.06
128	181.15	1412	0.12
129	182.15	2914	0.24
130	183.15	6155	0.51
131	184.15	4073	0.34
132	185.05	4157	0.34
133	186.05	3933	0.32
134	187.05	1321	0.11
135	188.10	698	0.06
136	189.10	278	0.02
137	193.10	220	0.02
138	196.10	201	0.02
139	197.25	338	0.03
140	198.25	790	0.07
141	199.15	9156	0.76
142	200.10	1450	0.12
143	206.20	2641	0.22
144	207.30	396	0.03
145	208.30	354	0.03
146	209.15	350	0.03
147	210.10	1562	0.13

#	m/z	Abs. Int	Rel. Int.
148	211.10	2118	0.17
149	212.05	3257	0.27
150	213.15	1940	0.16
151	214.10	5497	0.45
152	215.10	1975	0.16
153	216.10	1086	0.09
154	217.10	279	0.02
155	223.20	972	0.08
156	224.20	2063	0.17
157	225.15	4873	0.40
158	226.10	2642	0.22
159	227.10	5913	0.49
160	228.10	3570	0.29
161	229.10	1793	0.15
162	230.15	881	0.07
163	231.10	250	0.02
164	239.25	551	0.05
165	240.20	220	0.02
166	241.20	247	0.02
167	242.20	209	0.02
168	249.15	781	0.06
169	250.15	524	0.04
170	251.15	8107	0.67
171	252.15	8960	0.74
172	253.10	21750	1.80
173	254.15	4718	0.39
174	255.10	43601	3.60
175	256.05	8298	0.69
176	257.15	9296	0.77
177	258.05	1894	0.16
178	259.10	476	0.04
179	260.15	669	0.06
180	261.15	606	0.05
181	262.15	1108	0.09
182	263.15	853	0.07
183	264.25	876	0.07
184	265.20	550	0.05
185	266.15	374	0.03
186	267.15	389	0.03
187	268.15	723	0.06
188	269.15	466	0.04
189	270.10	1394	0.12
190	271.20	660	0.05
191	272.15	1298	0.11
192	273.05	516	0.04
193	274.00	450	0.04
194	275.20	390	0.03
195	276.20	238	0.02
196	281.20	337	0.03
197	283.10	562	0.05
198	284.10	273	0.02
199	285.20	428	0.04
200	287.20	241	0.02
201	293.20	346	0.03
202	294.15	368	0.03
203	295.15	746	0.06
204	296.15	453	0.04
205	297.20	1457	0.12
206	298.15	822	0.07
207	299.20	889	0.07
208	300.15	770	0.06
209	301.15	1391	0.11
210	302.20	1428	0.12
211	303.20	2758	0.23
212	304.20	1407	0.12
213	305.20	3174	0.26
214	306.15	874	0.07
215	307.20	607	0.05
216	310.50	337	0.03

#	m/z	Abs. In	Rel. Int.	#	m/z	Abs. In	Rel. Int.	#	m/z	Abs. In	Rel. Int.
217	311.45	424	0.04	233	344.25	389	0.03	249	380.20	218	0.02
218	312.45	517	0.04	234	345.20	1706	0.14	250	381.20	287	0.02
219	313.50	1609	0.13	235	346.20	561	0.05	251	383.20	215	0.02
220	314.45	916	0.08	236	347.25	485	0.04	252	390.20	209	0.02
221	315.25	566	0.05	237	348.30	214	0.02	253	404.25	530	0.04
222	316.25	555	0.05	238	353.30	204	0.02	254	405.25	900	0.07
223	317.20	849	0.07	239	354.30	222	0.02	255	406.30	270	0.02
224	318.20	660	0.05	240	355.45	482	0.04	256	414.30	212	0.02
225	319.20	295	0.02	241	366.35	412	0.03	257	416.25	2962	0.24
226	331.20	238	0.02	242	367.30	4286	0.35	258	417.25	2955	0.24
227	333.20	258	0.02	243	368.30	1478	0.12	259	418.20	7457	0.62
228	339.45	682	0.06	244	369.35	397	0.03	260	419.25	2100	0.17
229	340.20	218	0.02	245	375.30	207	0.02	261	420.20	14682	1.21
230	341.20	674	0.06	246	376.30	351	0.03	262	421.15	3707	0.31
231	342.25	627	0.05	247	377.30	220	0.02	263	422.20	3202	0.26
232	343.20	954	0.08	248	378.20	484	0.04	264	423.15	741	0.06

Mass chart of compound **9**

*Synthesis of N-(benzyloxy)-2-((4-formamidophenyl)selanyl)acetamide (**10**)*

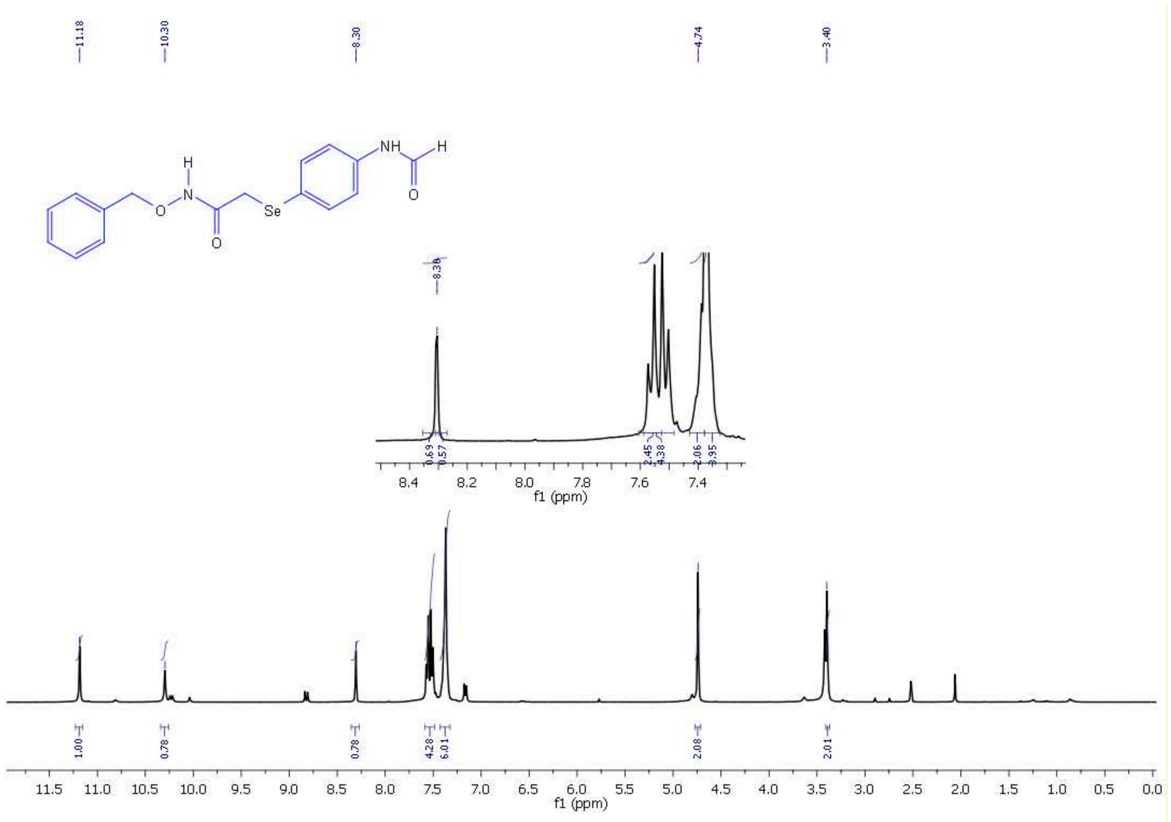

^{1}HNMR chart of compound **10**

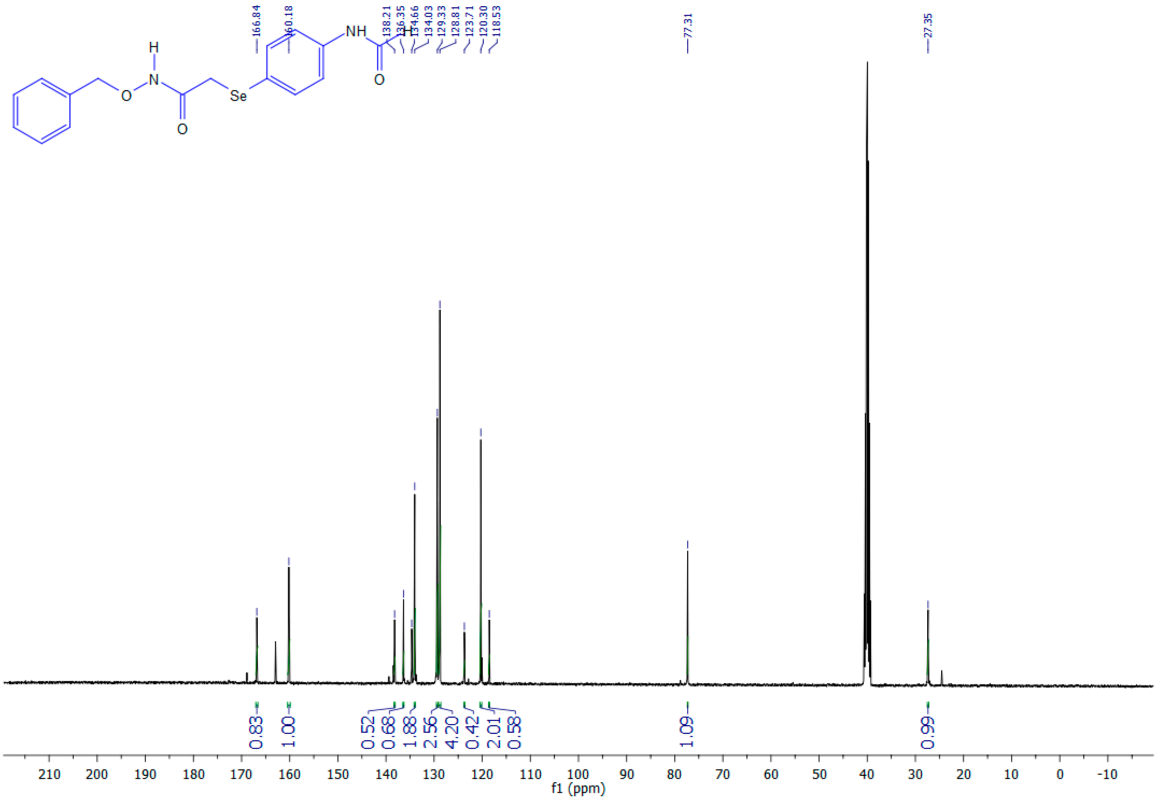

^{13}CNMR chart of compound **10**

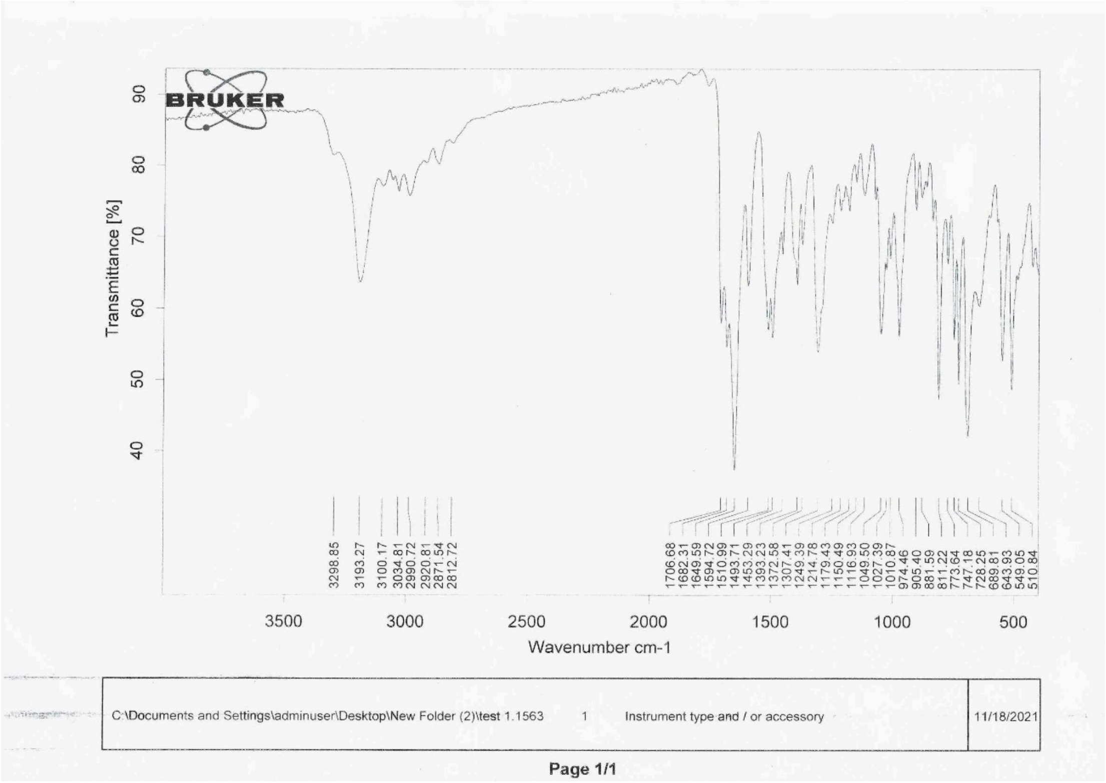

IR chart of compound of **10**

Cairo University
Micro Analytical Center

DI Analysis
Shimadzu Qp-2010 Plus

Sample Information

Analyzed by	: Dr. Mai Younis
Analyzed	: 06/01/2007 06:39:34 م
Sample Name	: J15
Sample ID	:
Customer Name	: Dr.Mohamed Soliman - Science - Cairo
Data File	: C:\GCMSsolution\Data\Project1\J15.QGD
Org Data File	: C:\GCMSsolution\Data\Project1\J15.QGD
Method File	: C:\GCMSsolution\Data\Project1\High Temperature Op
Org Method File	: C:\GCMSsolution\Data\Project1\High Temperature Op
Report File	:
Tuning File	: C:\GCMSsolution\System\Tune1_default.qgt
$EndIf$Modified by	: Dr. Mai Younis
Modified	: 06/01/2007 06:44:23 م

Method

==== Analytical Line 1 ====

IonSourceTemp	:250.00 °C
[MS Table]	
--Group 1 - Event 1--	
Start Time	:0.00min
End Time	:10.00min
ACQ Mode	:Scan
Event Time	:0.50sec
Scan Speed	:1000
Start m/z	:50.00
End m/z	:510.00
Electron Voltage	: 70 eV
Ionization Mode	: EI

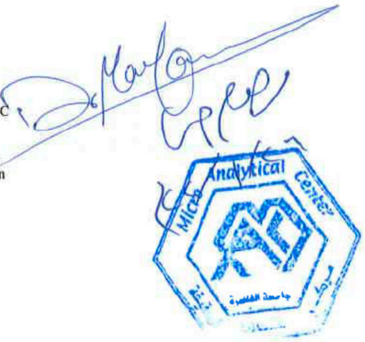

C:\GCMSsolution\Data\Project1\J15.QGD

Chromatogram J15 C:\GCMSsolution\Data\Project1\J15.QGD

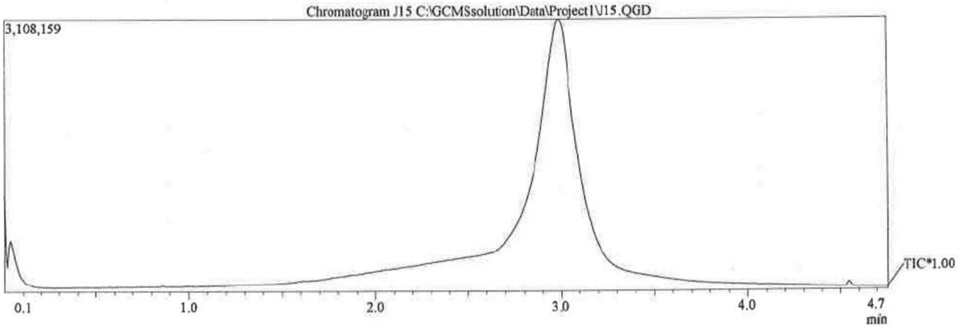

Spectrum

Line#:1 R.Time:3.0(Scan#:362)
MassPeaks:313(Peak Elimination m/z: 437.40, 455.40)
RawMode:Single 3.0(362) BasePeak:91(701185)
BG Mode:None Group 1 - Event 1

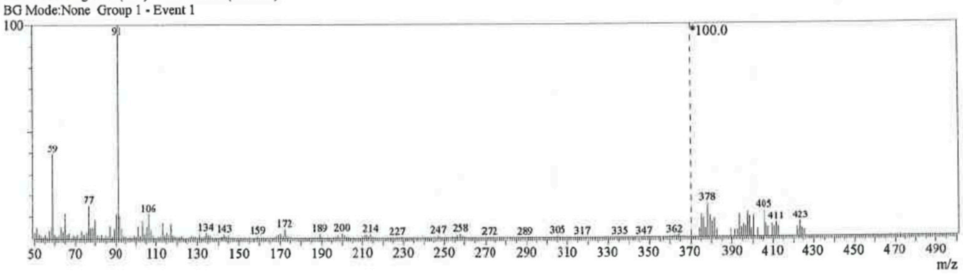

Mass Table
Line#:1 R.Time:3.0(Scan#:362)
MassPeaks:313(Peak Elimination m/z: 437.40, 455.40)
RawMode:Single 3.0(362) BasePeak:91(701185)
BG Mode:None Group 1 - Event 1

#	m/z	Abs. In	Rel. Int.	#	m/z	Abs. In	Rel. Int.	#	m/z	Abs. In	Rel. Int.
1	50.05	18306	2.61	4	53.05	9508	1.36	7	56.05	5385	0.77
2	51.00	35108	5.01	5	54.10	4943	0.70	8	57.05	27181	3.88
3	52.05	14886	2.12	6	55.05	11963	1.71	9	58.05	36337	5.18

#	m/z	Abs. In	Rel. Int.
10	59.05	277185	39.53
11	60.00	13026	1.86
12	61.05	6217	0.89
13	62.05	10649	1.52
14	63.05	36737	5.24
15	64.05	21412	3.05
16	65.05	80550	11.49
17	66.05	12945	1.85
18	67.00	17186	2.45
19	68.05	2987	0.43
20	69.05	11276	1.61
21	70.05	5218	0.74
22	71.00	12086	1.72
23	72.05	4991	0.71
24	73.05	24750	3.53
25	74.05	13753	1.96
26	75.05	13118	1.87
27	76.15	18283	2.61
28	77.05	107829	15.38
29	78.05	32785	4.68
30	79.05	34505	4.92
31	80.05	61428	8.76
32	81.05	10306	1.47
33	82.15	2853	0.41
34	83.05	12014	1.71
35	84.15	3523	0.50
36	85.10	10122	1.44
37	86.15	6782	0.97
38	87.05	41037	5.85
39	88.15	5806	0.83
40	89.10	31284	4.46
41	90.15	78211	11.15
42	91.10	701185	100.00
43	92.10	71798	10.24
44	93.05	32658	4.66
45	94.05	9575	1.37
46	95.05	8204	1.17
47	96.10	2594	0.37
48	97.10	6538	0.93
49	98.15	2925	0.42
50	99.10	8266	1.18
51	100.15	6500	0.93
52	101.10	38386	5.47
53	102.15	8334	1.19
54	103.15	55677	7.94
55	104.15	13353	1.90
56	105.15	36003	5.13
57	106.15	79678	11.36
58	107.15	25892	3.69
59	108.15	12012	1.71
60	109.15	3212	0.46
61	110.15	1564	0.22
62	111.15	6034	0.86
63	112.25	6235	0.89
64	113.15	49721	7.09
65	114.15	10159	1.45
66	115.15	17024	2.43
67	116.25	9691	1.38
68	117.20	47164	6.73
69	118.10	9286	1.32
70	119.10	6382	0.91
71	120.15	3172	0.45
72	121.20	4009	0.57
73	122.15	6660	0.95
74	123.15	1954	0.28
75	124.15	714	0.10
76	125.20	1495	0.21
77	126.25	2144	0.31
78	127.20	9042	1.29
79	128.20	4665	0.67
80	129.20	5682	0.81
81	130.20	3086	0.44
82	131.15	12803	1.83
83	132.15	4696	0.67
84	133.25	5619	0.80
85	134.20	16919	2.41
86	135.20	10226	1.46
87	136.20	8598	1.23
88	137.15	3534	0.50
89	138.10	1094	0.16
90	139.15	2197	0.31
91	140.10	1846	0.26
92	141.10	4375	0.62
93	142.15	3968	0.57
94	143.10	11502	1.64
95	144.10	5322	0.76
96	145.10	8807	1.26
97	146.10	2105	0.30
98	147.10	2425	0.35
99	148.10	1932	0.28
100	149.15	927	0.13
101	150.25	663	0.09
102	151.20	2362	0.34
103	152.15	1897	0.27
104	153.15	1488	0.21
105	154.15	925	0.13
106	155.15	2770	0.40
107	156.15	1100	0.16
108	157.15	1736	0.25
109	158.25	1263	0.18
110	159.20	6394	0.91
111	160.25	917	0.13
112	161.20	1492	0.21
113	162.15	984	0.14
114	163.20	511	0.07
115	164.05	304	0.04
116	165.05	813	0.12
117	166.05	1065	0.15
118	167.05	1810	0.26
119	168.05	6531	0.93
120	169.05	10292	1.47
121	170.05	15292	2.18
122	171.15	8248	1.18
123	172.05	27835	3.97
124	173.10	7732	1.10
125	174.05	5518	0.79
126	175.20	4231	0.60
127	176.20	1100	0.16
128	177.20	1340	0.19
129	178.20	316	0.05
130	179.10	246	0.04
131	180.15	574	0.08
132	181.15	782	0.11
133	182.15	1297	0.18
134	183.15	1889	0.27
135	184.10	1881	0.27
136	185.15	1969	0.28
137	186.10	2553	0.36
138	187.15	2322	0.33
139	188.25	1370	0.20
140	189.20	12263	1.75
141	190.15	2145	0.31
142	191.20	766	0.11
143	192.25	316	0.05
144	193.25	2281	0.33
145	194.10	905	0.13
146	195.05	520	0.07
147	196.05	3282	0.47
148	197.05	3342	0.48
149	198.00	7778	1.11
150	199.05	2393	0.34
151	200.05	14081	2.01
152	201.15	7510	1.07
153	202.10	3604	0.51
154	203.15	2020	0.29
155	204.15	654	0.09
156	205.20	498	0.07
157	207.20	298	0.04
158	208.15	385	0.05
159	209.15	743	0.11
160	210.15	3949	0.56
161	211.15	9729	1.39
162	212.10	9746	1.39
163	213.15	4324	0.62
164	214.05	10691	1.52
165	215.10	2289	0.33
166	216.05	2100	0.30
167	217.15	1409	0.20
168	218.10	412	0.06
169	219.20	799	0.11
170	220.20	225	0.03
171	221.20	225	0.03
172	224.15	728	0.10
173	225.15	1328	0.19
174	226.15	1083	0.15
175	227.15	1616	0.23
176	228.15	1472	0.21
177	229.20	1295	0.18
178	230.15	1025	0.15
179	231.15	896	0.13
180	232.05	314	0.04
181	233.20	660	0.09
182	234.30	311	0.04
183	235.15	440	0.06
184	236.20	212	0.03
185	237.20	204	0.03
186	238.15	380	0.05
187	239.20	908	0.13
188	240.15	548	0.08
189	241.15	735	0.10
190	242.20	950	0.14
191	243.00	438	0.06
192	244.15	298	0.04
193	245.20	983	0.14
194	246.35	617	0.09
195	247.30	5943	0.85
196	248.20	1900	0.27
197	249.15	441	0.06
198	251.15	428	0.06
199	252.10	753	0.11
200	253.15	677	0.10
201	254.10	3342	0.48
202	255.15	2531	0.36
203	256.10	7935	1.13
204	257.15	1989	0.28
205	258.10	12047	1.72
206	259.25	6053	0.86
207	260.15	3195	0.46
208	261.20	927	0.13
209	262.40	513	0.07
210	263.35	485	0.07
211	264.35	442	0.06
212	265.30	562	0.08
213	266.30	214	0.03
214	268.05	363	0.05
215	269.25	391	0.06
216	270.25	776	0.11

#	m/z	Abs. In	Rel. Int.
217	271.25	754	0.11
218	272.15	1314	0.19
219	273.25	723	0.10
220	274.15	465	0.07
221	275.20	356	0.05
222	276.20	276	0.04
223	277.30	554	0.08
224	285.30	580	0.08
225	286.35	372	0.05
226	287.30	478	0.07
227	288.30	394	0.06
228	289.25	1146	0.16
229	290.25	484	0.07
230	291.25	933	0.13
231	292.20	322	0.05
232	293.25	414	0.06
233	299.30	268	0.04
234	300.30	297	0.04
235	301.40	378	0.05
236	302.35	448	0.06
237	303.35	950	0.14
238	304.35	948	0.14
239	305.35	4728	0.67
240	306.30	1756	0.25
241	307.25	488	0.07
242	313.45	782	0.11
243	314.50	314	0.04
244	315.50	222	0.03
245	316.35	407	0.06
246	317.35	1606	0.23
247	318.15	573	0.08
248	319.15	451	0.06
249	320.10	247	0.04
250	321.30	521	0.07
251	323.30	593	0.08
252	329.30	220	0.03
253	331.30	476	0.07
254	332.30	209	0.03
255	333.45	408	0.06
256	334.50	286	0.04
257	335.50	602	0.09
258	336.50	238	0.03
259	337.50	201	0.03
260	339.40	582	0.08
261	341.40	252	0.04
262	343.40	212	0.03
263	346.40	218	0.03
264	347.35	1144	0.16
265	348.35	355	0.05
266	349.25	481	0.07
267	351.30	292	0.04
268	353.30	268	0.04
269	355.30	250	0.04
270	358.30	206	0.03
271	359.25	346	0.05
272	360.25	2084	0.30
273	361.25	2545	0.36
274	362.20	4982	0.71
275	363.25	2971	0.42
276	364.25	10056	1.43
277	365.15	2555	0.36
278	366.20	1932	0.28
279	367.30	900	0.13
280	368.30	241	0.03
281	374.40	263	0.04
282	375.45	725	0.10
283	376.35	613	0.09
284	377.30	273	0.04
285	378.30	1097	0.16
286	379.40	678	0.10
287	380.40	484	0.07
288	381.50	585	0.08
289	382.50	222	0.03
290	389.50	250	0.04
291	391.50	207	0.03
292	392.60	217	0.03
293	393.60	735	0.10
294	394.60	290	0.04
295	395.55	387	0.06
296	396.50	324	0.05
297	397.50	818	0.12
298	398.45	644	0.09
299	399.20	255	0.04
300	400.20	676	0.10
301	402.20	255	0.04
302	405.45	851	0.12
303	406.50	409	0.06
304	407.50	302	0.04
305	409.50	404	0.06
306	410.50	303	0.04
307	411.55	459	0.07
308	412.55	313	0.04
309	421.60	298	0.04
310	422.40	202	0.03
311	423.45	478	0.07
312	424.40	265	0.04
313	425.40	207	0.03

Mass chart of compound **10**

*Synthesis of 4-((4-((2-((benzyloxy)amino)-2-oxoethyl)selanyl)phenyl)amino)-4-oxobutanoic acid (**11**)*

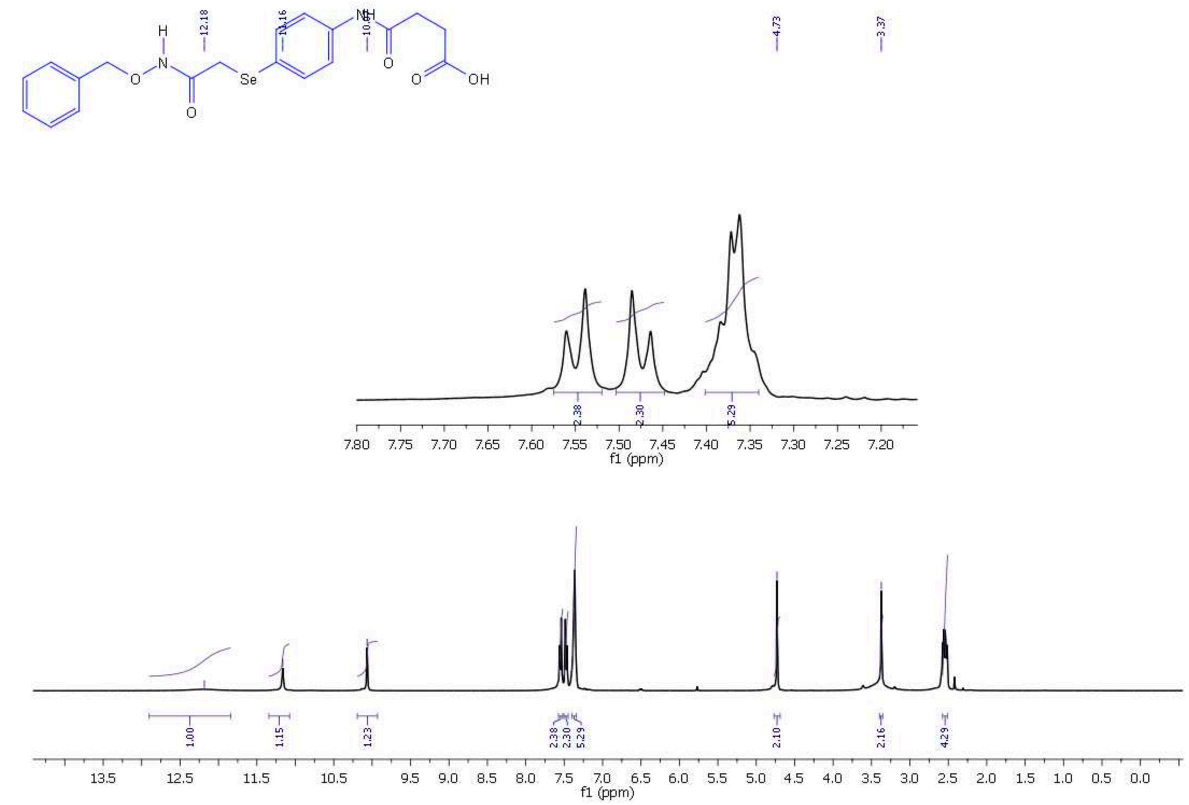

^{1}HNMR chart of compound **11**

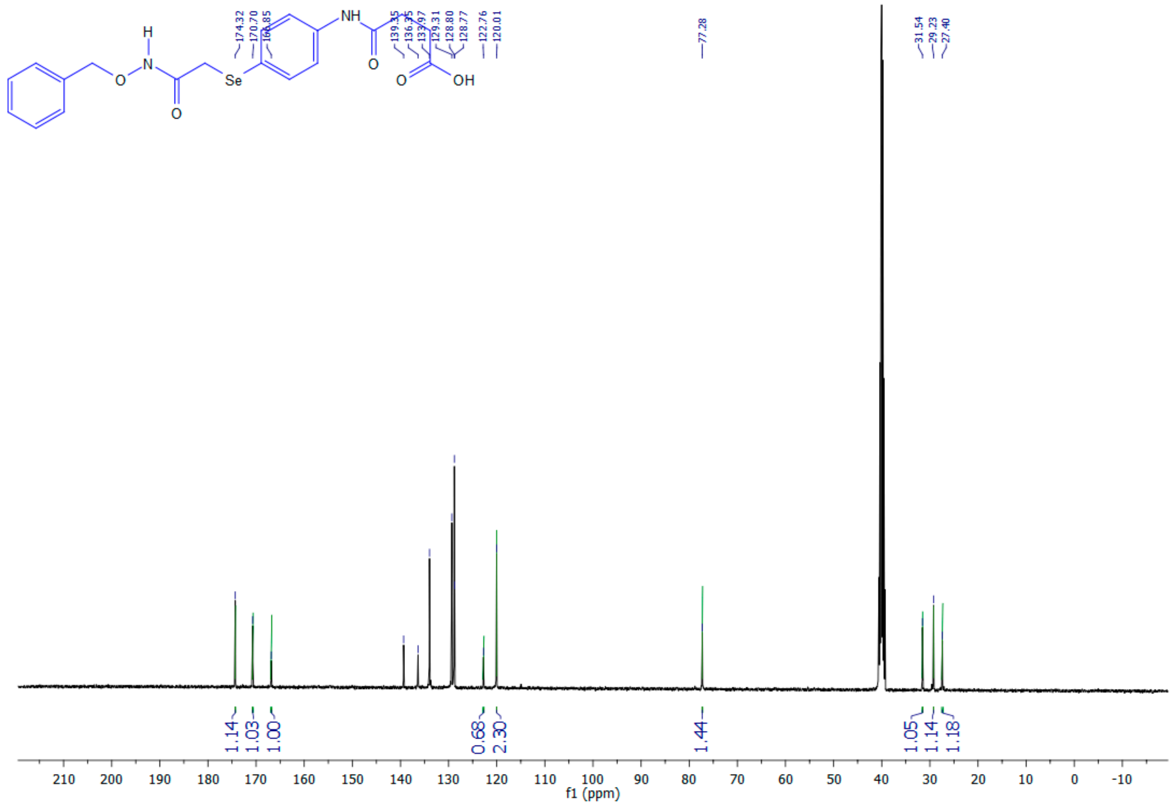

^{1}HNMR chart of compound **11**

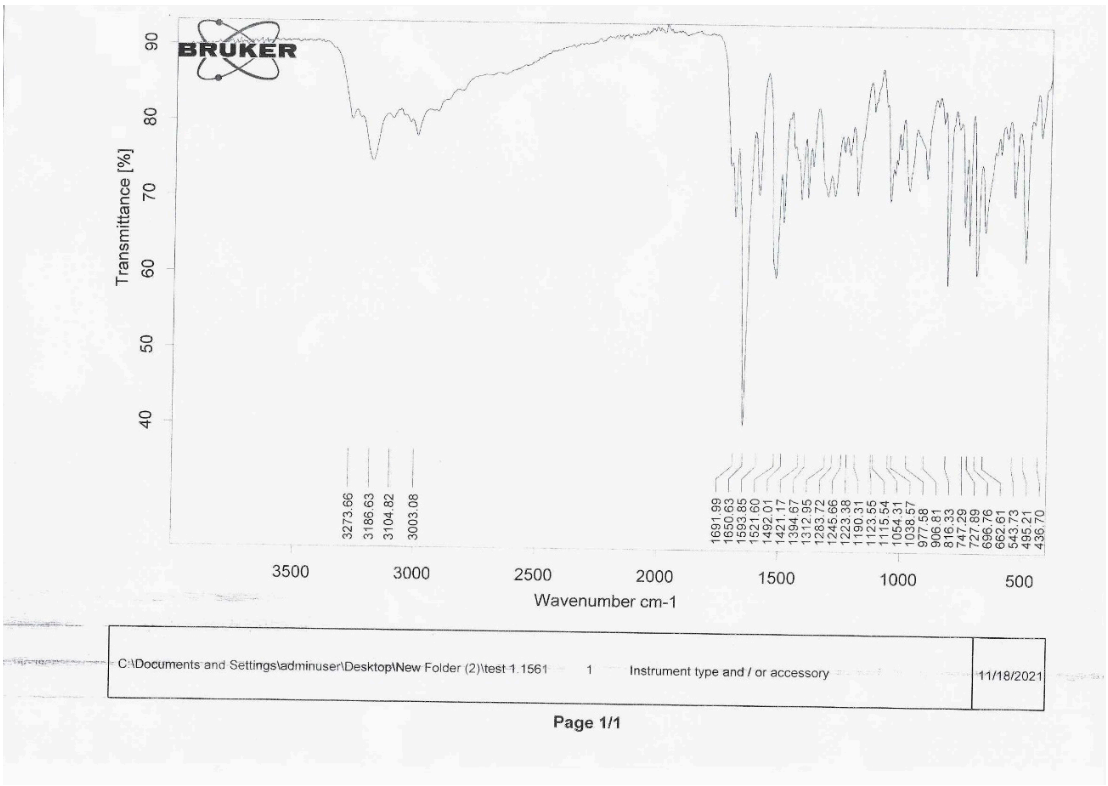

IR chart of compound **11**

Cairo University
Micro Analytical Center

DI Analysis
Shimadzu Qp-2010 Plus

Sample Information

Analyzed by	: Dr. Mai Younis
Analyzed	: 03/01/2007 09:00:00 م
Sample Name	: J10
Sample ID	:
Customer Name	: Dr.Mohamed Soliman - Science - Cairo
Data File	: C:\GCMSsolution\Data\Project1\J10.QGD
Org Data File	: C:\GCMSsolution\Data\Project1\J10.QGD
Method File	: C:\GCMSsolution\Data\Project1\High Temperature Op
Org Method File	: C:\GCMSsolution\Data\Project1\High Temperature Op
Report File	:
Tuning File	: C:\GCMSsolution\System\Tune1_default.qgt
$EndIf$Modified by	: Dr. Mai Younis
Modified	: 03/01/2007 09:05:28 م

Method

==== Analytical Line 1 ====

IonSourceTemp	:250.00 °C
[MS Table]	
--Group 1 - Event 1--	
Start Time	:0.00min
End Time	:10.00min
ACQ Mode	:Scan
Event Time	:0.50sec
Scan Speed	:1000
Start m/z	:50.00
End m/z	:510.00
Electron Voltage	:70 eV
Ionization Mode	:EI

C:\GCMSsolution\Data\Project1\J10.QGD

Chromatogram J10 C:\GCMSsolution\Data\Project1\J10.QGD

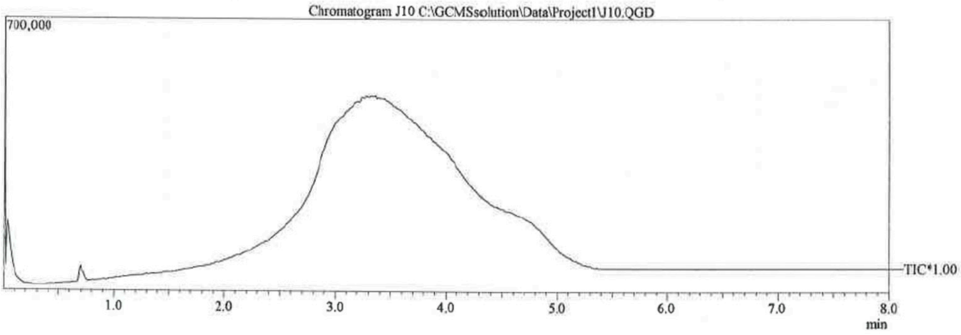

Spectrum

Line#:1 R.Time:3.8(Scan#:457)
MassPeaks:181
RawMode:Single 3.8(457) BasePeak:91(115204)
BG Mode:None Group 1 - Event 1

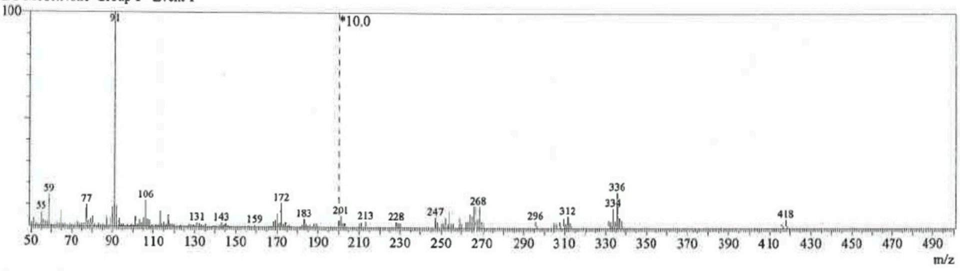

Mass Table
Line#:1 R.Time:3.8(Scan#:457)
MassPeaks:181
RawMode:Single 3.8(457) BasePeak:91(115204)
BG Mode:None Group 1 - Event 1

#	m/z	Abs. In	Rel. Int.	#	m/z	Abs. In	Rel. Int.	#	m/z	Abs. In	Rel. Int.
1	50.05	1945	1.69	4	53.05	1292	1.12	7	56.00	3238	2.81
2	51.00	4190	3.64	5	54.05	1117	0.97	8	57.05	2271	1.97
3	52.05	1636	1.42	6	55.00	7308	6.34	9	58.05	2379	2.07

03-Jan-07 21:16:28

#	m/z	Abs. In	Rel. Int.	#	m/z	Abs. In	Rel. Int.	#	m/z	Abs. In	Rel. Int.
10	59.00	16964	14.73	68	117.15	6087	5.28	126	186.00	1239	1.08
11	60.00	849	0.74	69	118.05	1331	1.16	127	187.10	540	0.47
12	61.00	436	0.38	70	119.10	1262	1.10	128	188.05	1709	1.48
13	62.05	769	0.67	71	120.10	1086	0.94	129	189.15	1769	1.54
14	63.05	2693	2.34	72	121.15	360	0.31	130	190.05	773	0.67
15	64.05	1718	1.49	73	122.10	246	0.21	131	191.00	321	0.28
16	65.00	8239	7.15	74	123.10	633	0.55	132	193.00	209	0.18
17	66.05	1119	0.97	75	125.10	281	0.24	133	196.00	231	0.20
18	67.00	1436	1.25	76	126.25	325	0.28	134	198.00	418	0.36
19	68.00	274	0.24	77	127.20	1298	1.13	135	200.00	230	0.20
20	69.05	1175	1.02	78	128.15	883	0.77	136	201.10	598	0.52
21	70.05	615	0.53	79	129.15	915	0.79	137	202.10	210	0.18
22	71.05	1172	1.02	80	130.25	648	0.56	138	203.10	202	0.18
23	72.05	500	0.43	81	131.15	2109	1.83	139	210.10	207	0.18
24	73.05	2134	1.85	82	132.15	1716	1.49	140	211.10	226	0.20
25	74.05	1019	0.88	83	133.20	889	0.77	141	213.10	302	0.26
26	75.05	1291	1.12	84	134.15	605	0.53	142	228.10	263	0.23
27	76.05	1732	1.50	85	135.15	1346	1.17	143	229.10	212	0.18
28	77.05	11559	10.03	86	136.10	202	0.18	144	230.10	222	0.19
29	78.05	3103	2.69	87	139.20	382	0.33	145	247.20	534	0.46
30	79.05	3815	3.31	88	140.05	376	0.33	146	248.20	295	0.26
31	80.05	4986	4.33	89	141.05	691	0.60	147	250.20	238	0.21
32	81.05	1216	1.06	90	142.05	668	0.58	148	251.00	239	0.21
33	82.05	454	0.39	91	143.00	1937	1.68	149	251.95	486	0.42
34	83.10	1522	1.32	92	144.05	845	0.73	150	253.10	210	0.18
35	84.10	582	0.51	93	145.05	1680	1.46	151	254.10	846	0.73
36	85.10	1159	1.01	94	146.15	764	0.66	152	255.10	218	0.19
37	86.15	703	0.61	95	147.15	573	0.50	153	256.10	222	0.19
38	87.05	4634	4.02	96	148.20	258	0.22	154	259.20	506	0.44
39	88.15	749	0.65	97	149.20	242	0.21	155	260.20	236	0.20
40	89.15	4402	3.82	98	151.20	247	0.21	156	262.20	300	0.26
41	90.15	10221	8.87	99	153.10	356	0.31	157	263.10	329	0.29
42	91.05	115204	100.00	100	154.05	368	0.32	158	264.15	694	0.60
43	92.05	11047	9.59	101	155.10	348	0.30	159	265.15	589	0.51
44	93.05	4079	3.54	102	156.10	519	0.45	160	266.10	1119	0.97
45	94.05	1002	0.87	103	157.15	393	0.34	161	267.15	437	0.38
46	95.10	1118	0.97	104	158.10	351	0.30	162	268.10	1127	0.98
47	96.10	449	0.39	105	159.10	911	0.79	163	269.10	337	0.29
48	97.05	904	0.78	106	160.10	310	0.27	164	270.10	255	0.22
49	98.15	458	0.40	107	165.10	242	0.21	165	296.10	330	0.29
50	99.10	1004	0.87	108	166.05	539	0.47	166	305.10	279	0.24
51	100.15	865	0.75	109	167.05	580	0.50	167	306.10	233	0.20
52	101.10	5194	4.51	110	168.05	2882	2.50	168	308.10	305	0.26
53	102.15	981	0.85	111	169.05	3305	2.87	169	310.10	487	0.42
54	103.10	3543	3.08	112	170.00	6798	5.90	170	311.10	220	0.19
55	104.15	1784	1.55	113	171.05	1859	1.61	171	312.10	607	0.53
56	105.10	4430	3.85	114	172.00	12639	10.97	172	313.10	270	0.23
57	106.10	14001	12.15	115	172.95	1539	1.34	173	332.15	399	0.35
58	107.10	3910	3.39	116	174.00	2425	2.10	174	333.15	338	0.29
59	108.10	3198	2.78	117	175.10	649	0.56	175	334.15	1050	0.91
60	109.10	834	0.72	118	176.10	1036	0.90	176	335.25	356	0.31
61	110.15	298	0.26	119	177.10	318	0.28	177	336.15	1897	1.65
62	111.05	868	0.75	120	180.10	318	0.28	178	337.10	524	0.45
63	112.15	789	0.68	121	181.15	378	0.33	179	338.20	398	0.35
64	113.10	7921	6.88	122	182.10	1002	0.87	180	416.20	215	0.19
65	114.10	1380	1.20	123	183.10	4110	3.57	181	418.25	456	0.40
66	115.10	2383	2.07	124	184.05	1558	1.35				
67	116.15	1146	0.99	125	185.05	643	0.56				

2 / 2

Mass chart of compound **11**

*Synthesis of 4-((4-((2-((benzyloxy)amino)-2-oxoethyl)selanyl)phenyl)amino)-4-oxobut-2-enoic acid (**12**)*

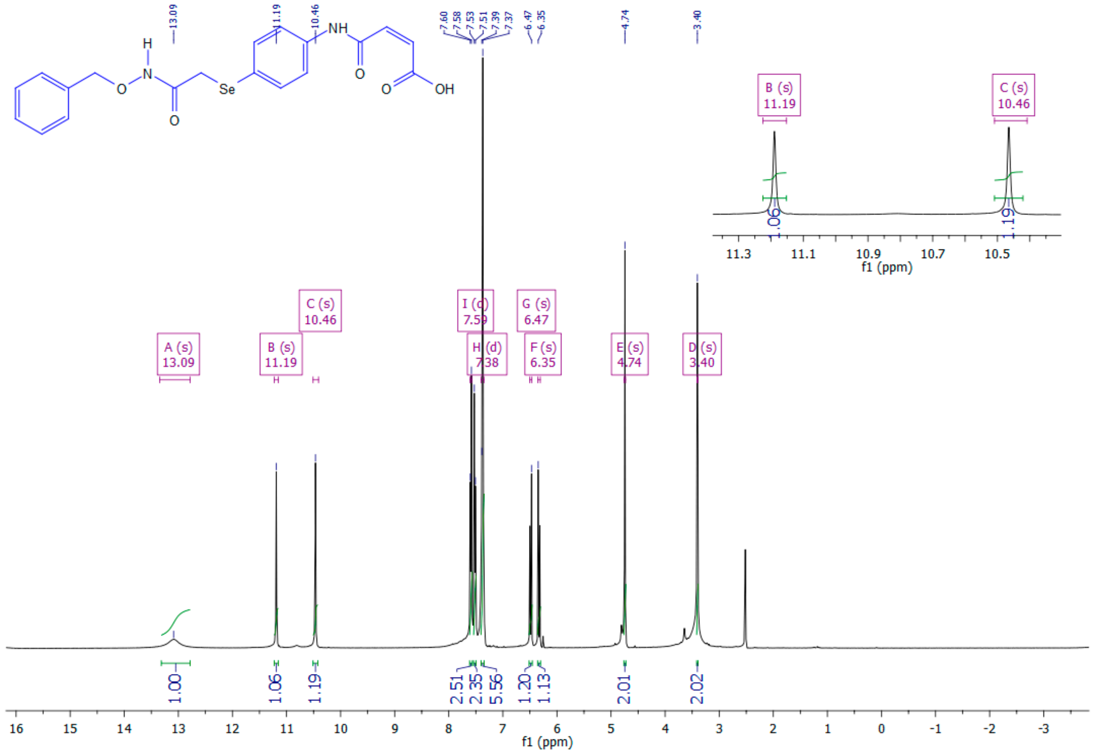

^{1}HNMR chart of compound **12**

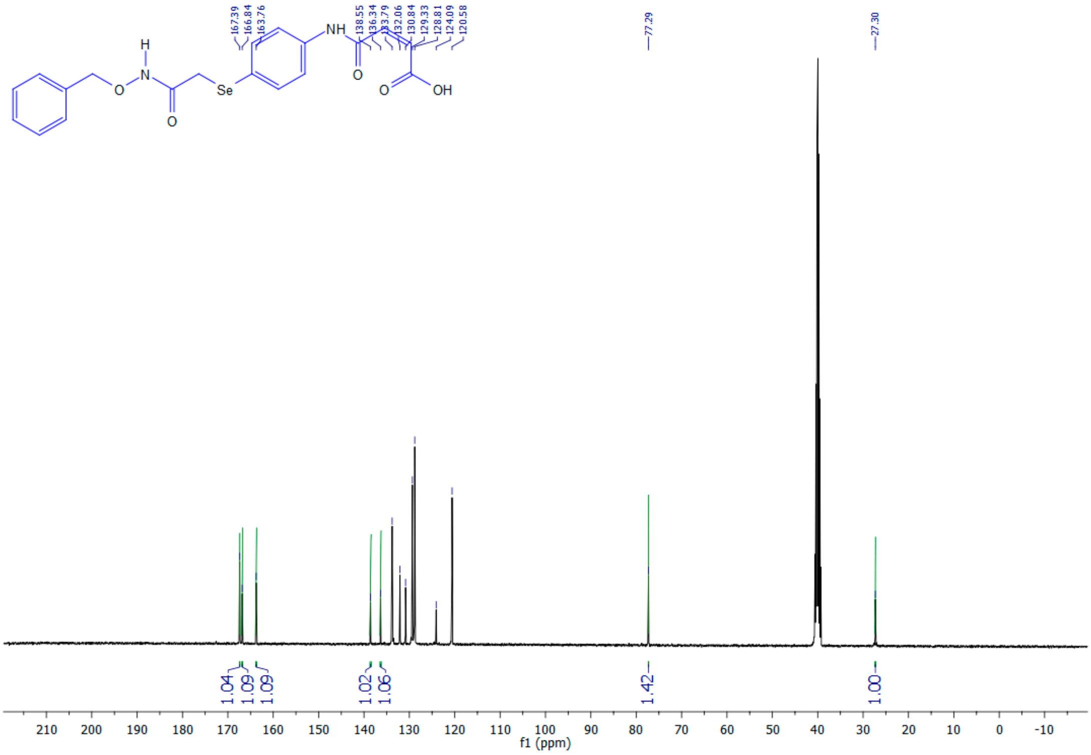

^{13}CNMR chart of compound **12**

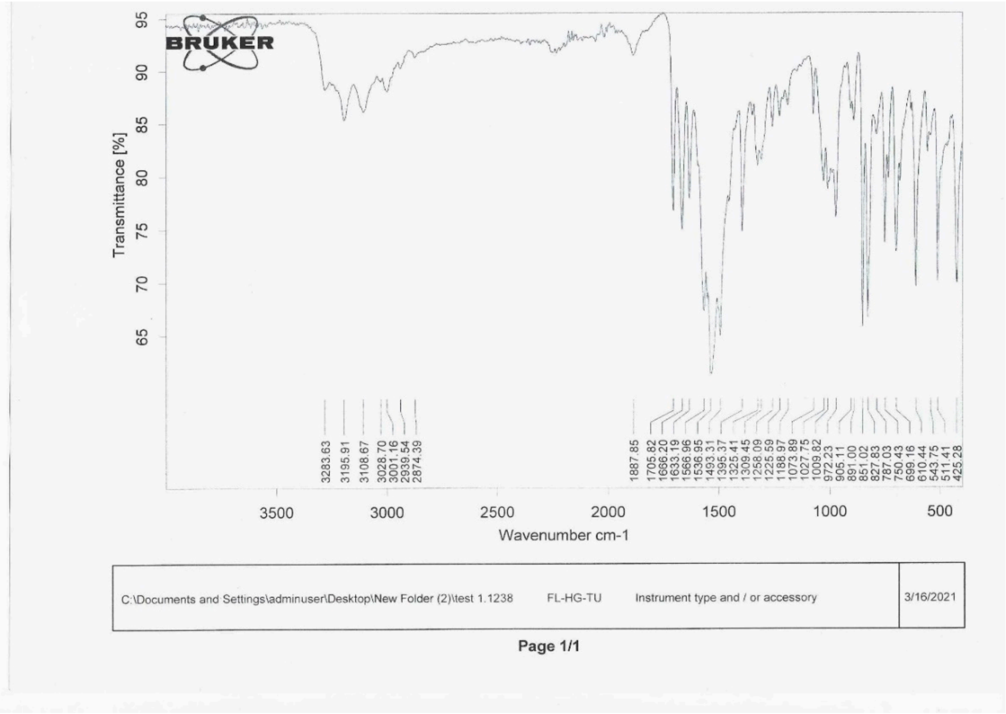

IR chart of compound **12**

Cairo University
Micro Analytical Center

DI Analysis
Shimadzu Qp-2010 Plus

Sample Information

Analyzed by	: Dr. Mai Younis
Analyzed	: 03/01/2007 09:07:47 م
Sample Name	: J6
Sample ID	:
Customer Name	: Dr.Mohamed Soliman - Science - Cairo
Data File	: C:\GCMSsolution\Data\Project1\J6.QGD
Org Data File	: C:\GCMSsolution\Data\Project1\J6.QGD
Method File	: C:\GCMSsolution\Data\Project1\High Temperature Op
Org Method File	: C:\GCMSsolution\Data\Project1\High Temperature Op
Report File	:
Tuning File	: C:\GCMSsolution\System\Tune1_default.qgt
$EndIf$Modified by	: Dr. Mai Younis
Modified	: 03/01/2007 09:12:26 م

Method

==== Analytical Line 1 ====

IonSourceTemp	:250.00 °C
[MS Table]	
--Group 1 - Event 1--	
Start Time	:0.00min
End Time	:10.00min
ACQ Mode	:Scan
Event Time	:0.50sec
Scan Speed	:1000
Start m/z	:50.00
End m/z	:510.00
Electron Voltage	: 70 eV
Ionization Mode	: EI

C:\GCMSsolution\Data\Project1\J6.QGD

Chromatogram J6 C:\GCMSsolution\Data\Project1\J6.QGD

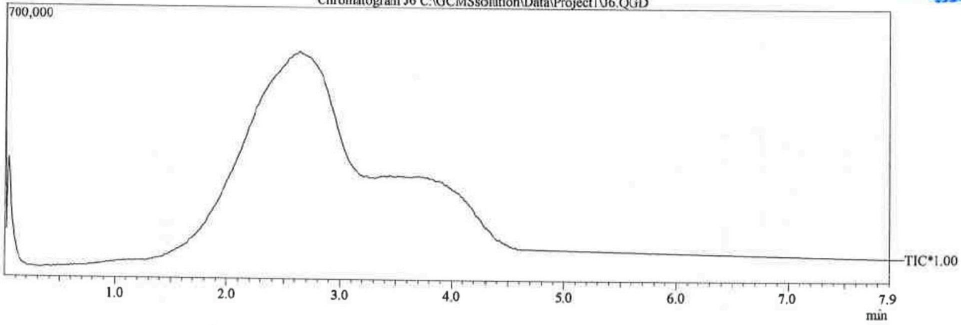

Spectrum

Line#:1 R.Time:2.6(Scan#:318)
MassPeaks:155
RawMode:Single 2.6(318) BasePeak:91(160524)
BG Mode:None Group 1 - Event 1

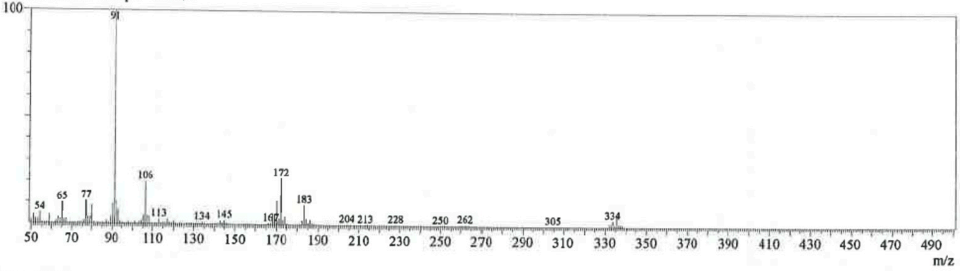

Mass Table
Line#:1 R.Time:2.6(Scan#:318)
MassPeaks:155
RawMode:Single 2.6(318) BasePeak:91(160524)
BG Mode:None Group 1 - Event 1

#	m/z	Abs. In	Rel. Int.	#	m/z	Abs. In	Rel. Int.	#	m/z	Abs. In	Rel. Int.
1	50.00	2658	1.66	4	53.05	3473	2.16	7	56.05	550	0.34
2	51.00	6460	4.02	5	54.00	8020	5.00	8	57.05	1150	0.72
3	52.00	3343	2.08	6	55.00	1062	0.66	9	58.05	1191	0.74

#	m/z	Abs. In	Rel. Int.	#	m/z	Abs. In	Rel. Int.	#	m/z	Abs. In	Rel. Int.
10	59.00	6412	3.99	59	108.10	5361	3.34	108	165.05	403	0.25
11	60.05	499	0.31	60	109.15	1139	0.71	109	166.00	958	0.60
12	61.05	407	0.25	61	110.20	305	0.19	110	167.05	986	0.61
13	62.05	1434	0.89	62	111.10	540	0.34	111	168.00	7663	4.77
14	63.00	4708	2.93	63	112.15	387	0.24	112	169.05	7641	4.76
15	64.05	3127	1.95	64	113.10	3642	2.27	113	170.00	17698	11.03
16	65.00	15834	9.86	65	114.10	929	0.58	114	171.05	4402	2.74
17	66.05	2618	1.63	66	115.05	1776	1.11	115	172.00	34906	21.75
18	67.05	2996	1.87	67	116.10	1247	0.78	116	172.95	3390	2.11
19	68.00	295	0.18	68	117.05	3574	2.23	117	174.00	6183	3.85
20	69.05	642	0.40	69	118.05	1225	0.76	118	174.95	781	0.49
21	70.05	500	0.31	70	119.05	1079	0.67	119	180.10	505	0.31
22	71.00	689	0.43	71	120.10	2570	1.60	120	181.15	596	0.37
23	72.05	405	0.25	72	121.10	591	0.37	121	182.15	3140	1.96
24	73.05	1073	0.67	73	122.10	236	0.15	122	183.10	14934	9.30
25	74.00	1164	0.73	74	123.10	1327	0.83	123	184.05	4336	2.70
26	75.05	1243	0.77	75	124.15	399	0.25	124	185.05	1336	0.83
27	76.05	2258	1.41	76	125.20	247	0.15	125	186.00	3825	2.38
28	77.05	17187	10.71	77	126.10	202	0.13	126	186.90	828	0.52
29	78.00	4534	2.82	78	127.05	737	0.46	127	187.95	821	0.51
30	79.05	4743	2.95	79	128.15	717	0.45	128	189.10	751	0.47
31	80.05	13542	8.44	80	129.10	815	0.51	129	204.20	516	0.32
32	81.00	1236	0.77	81	130.10	620	0.39	130	213.10	366	0.23
33	82.10	329	0.20	82	131.10	1158	0.72	131	214.00	358	0.22
34	83.10	711	0.44	83	132.10	758	0.47	132	226.00	281	0.18
35	84.05	368	0.23	84	133.15	806	0.50	133	228.00	572	0.36
36	85.10	649	0.40	85	134.10	1457	0.91	134	229.00	206	0.13
37	86.15	501	0.31	86	135.15	923	0.57	135	230.00	415	0.26
38	87.05	2214	1.38	87	139.10	342	0.21	136	250.00	231	0.14
39	88.15	823	0.51	88	140.00	332	0.21	137	252.00	313	0.19
40	89.15	5673	3.53	89	141.00	1113	0.69	138	259.20	471	0.29
41	90.15	14663	9.13	90	142.00	985	0.61	139	260.15	419	0.26
42	91.05	160524	100.00	91	143.05	2124	1.32	140	261.10	324	0.20
43	92.05	17009	10.60	92	144.05	1332	0.83	141	262.15	955	0.59
44	93.05	10578	6.59	93	145.00	2942	1.83	142	263.15	580	0.36
45	94.05	2029	1.26	94	146.10	1478	0.92	143	264.20	514	0.32
46	95.05	944	0.59	95	147.00	886	0.55	144	265.20	263	0.16
47	95.95	397	0.25	96	148.00	308	0.19	145	266.20	278	0.17
48	97.05	668	0.42	97	149.00	294	0.18	146	305.20	217	0.14
49	98.05	2091	1.30	98	151.00	228	0.14	147	330.20	210	0.13
50	99.10	718	0.45	99	152.00	241	0.15	148	332.20	1775	1.11
51	100.15	447	0.28	100	153.00	247	0.15	149	333.15	1844	1.15
52	101.10	2162	1.35	101	154.00	279	0.17	150	334.15	4462	2.78
53	102.05	672	0.42	102	155.00	318	0.20	151	335.20	1271	0.79
54	103.10	1687	1.05	103	156.00	302	0.19	152	336.15	8917	5.55
55	104.15	2403	1.50	104	157.00	351	0.22	153	337.15	1686	1.05
56	105.15	6490	4.04	105	158.00	441	0.27	154	338.15	1717	1.07
57	106.10	31970	19.92	106	159.20	439	0.27	155	339.05	308	0.19
58	107.10	5873	3.66	107	160.10	340	0.21				

Mass chart of compound **12**

*Synthesis of N-(benzyloxy)-2-((4-(1,3-dioxoisoindolin-2-yl)phenyl)selanyl)acetamide (**13**)*

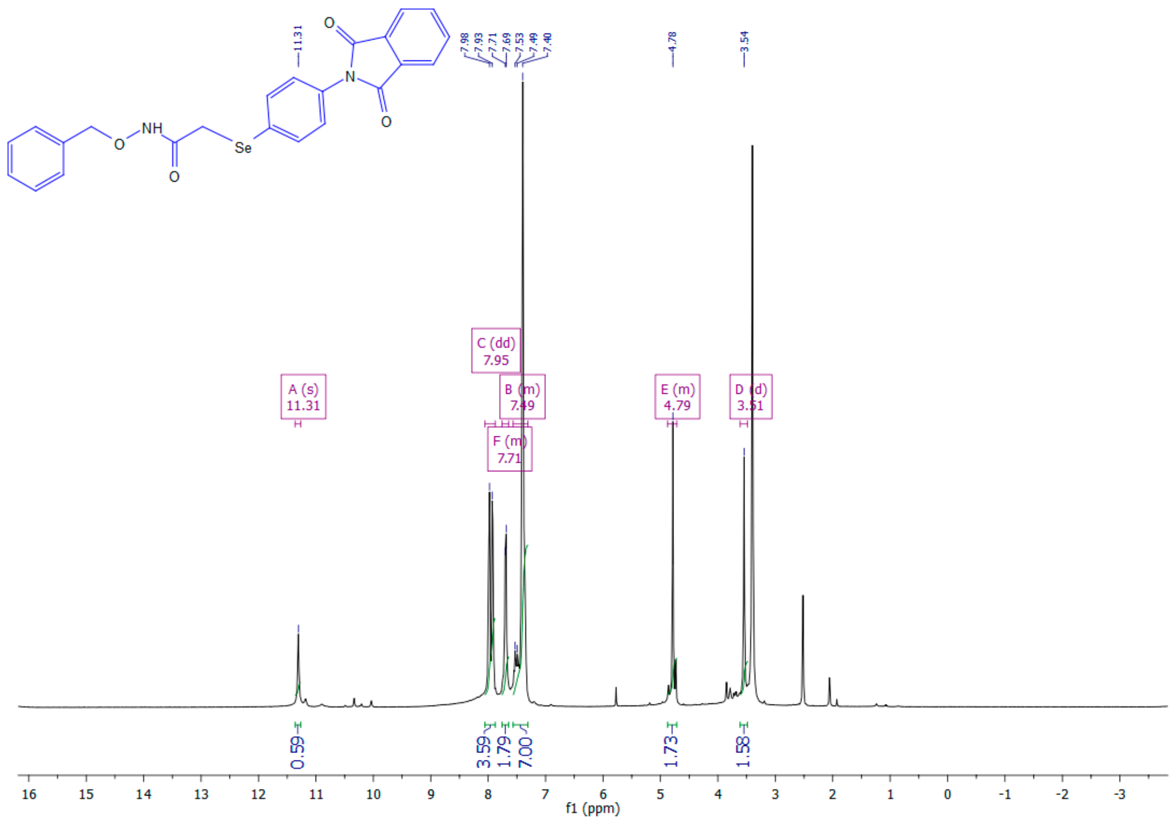

^{1}HNMR chart of compound **13**

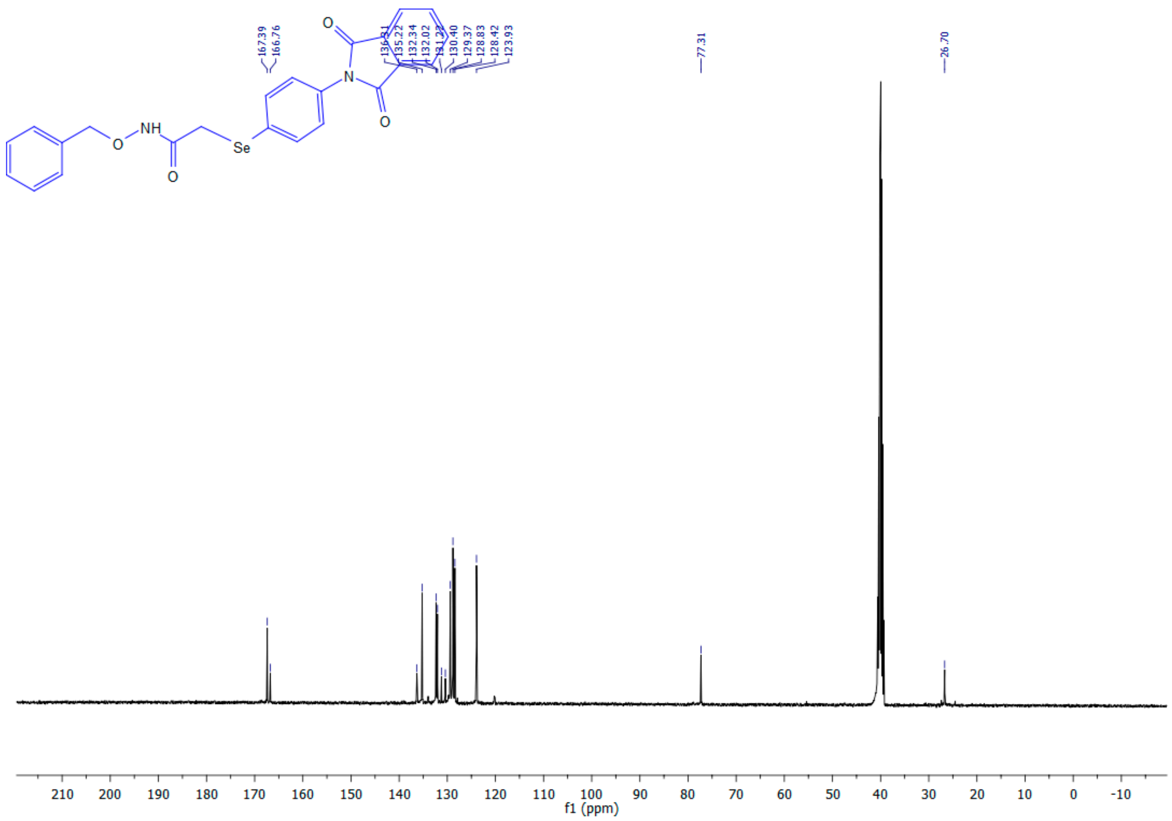

^{13}CNMR chart of compound **13**

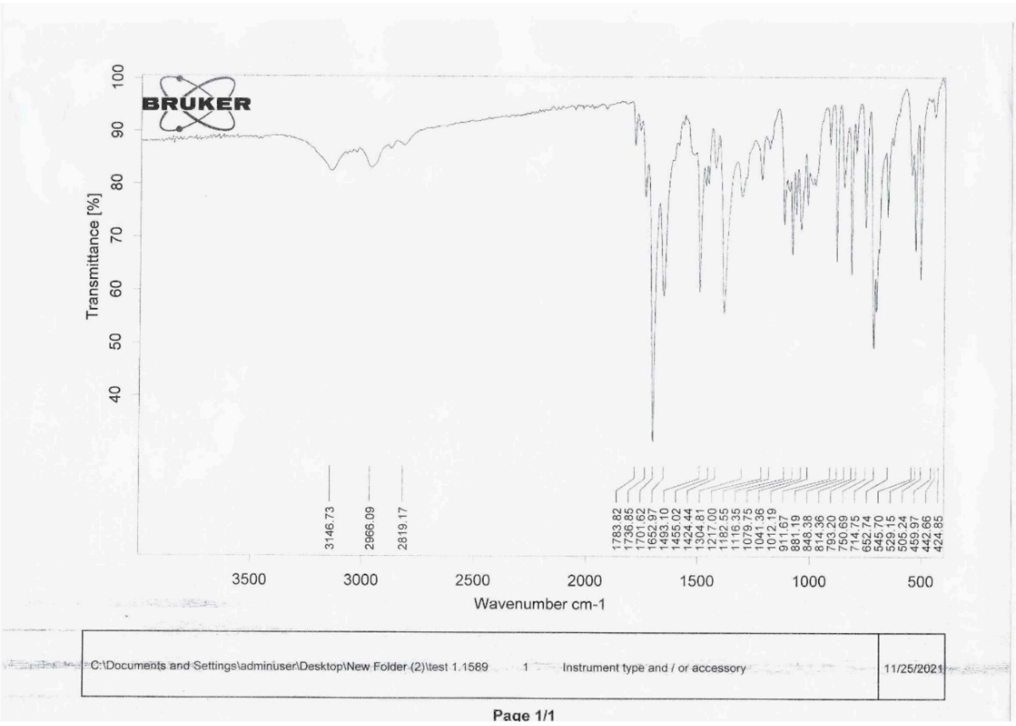

IR chart of compound **13**

06-Jan-07 18:31:14

Cairo University
Micro Analytical Center

DI Analysis
Shimadzu Qp-2010 Plus

Sample Information

Analyzed by	: Dr. Mai Younis
Analyzed	: 06/01/2007 06:25:47 م
Sample Name	: J16
Sample ID	:
Customer Name	: Dr.Mohamed Soliman - Science - Cairo
Data File	: C:\GCMSsolution\Data\Project1\J16.QGD
Org Data File	: C:\GCMSsolution\Data\Project1\J16.QGD
Method File	: C:\GCMSsolution\Data\Project1\High Temperature Op
Org Method File	: C:\GCMSsolution\Data\Project1\High Temperature Op
Report File	:
Tuning File	: C:\GCMSsolution\System\Tune1_default.qgt
$EndIf$Modified by	: Dr. Mai Younis
Modified	: 06/01/2007 06:29:55 م

Method

==== Analytical Line 1 ====

IonSourceTemp	:250.00 °C
[MS Table]	
--Group 1 - Event 1--	
Start Time	:0.00min
End Time	:10.00min
ACQ Mode	:Scan
Event Time	:0.50sec
Scan Speed	:1000
Start m/z	:50.00
End m/z	:510.00
Electron Voltage	: 70 eV
Ionization Mode	: EI

C:\GCMSsolution\Data\Project1\J16.QGD

Chromatogram J16 C:\GCMSsolution\Data\Project1\J16.QGD

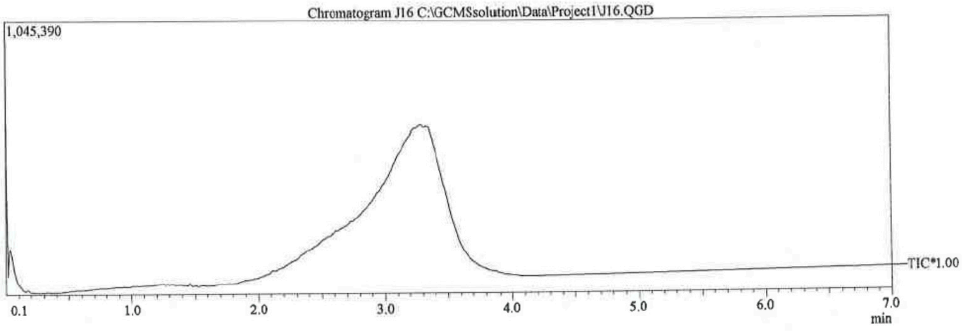

Spectrum

Line#:1 R.Time:3.3(Scan#:391)
MassPeaks:187
RawMode:Single 3.3(391) BasePeak:91(208863)
BG Mode:None Group 1 - Event 1

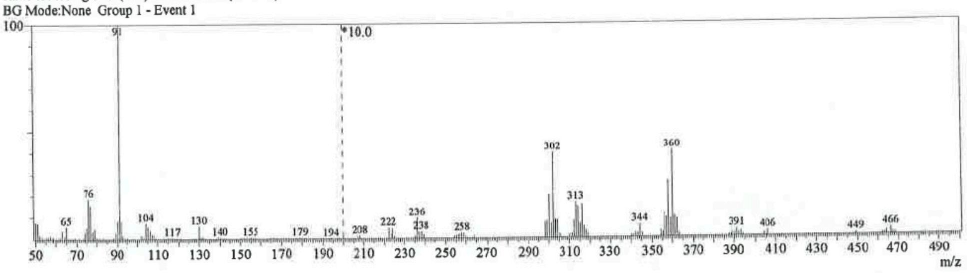

Mass Table
Line#:1 R.Time:3.3(Scan#:391)
MassPeaks:187
RawMode:Single 3.3(391) BasePeak:91(208863)
BG Mode:None Group 1 - Event 1

#	m/z	Abs. In	Rel. Int.	#	m/z	Abs. In	Rel. Int.	#	m/z	Abs. In	Rel. Int.
1	50.00	16015	7.67	4	53.05	1982	0.95	7	56.05	1571	0.75
2	51.00	15266	7.31	5	54.05	690	0.33	8	57.05	2674	1.28
3	52.00	3894	1.86	6	55.05	2418	1.16	9	58.05	2004	0.96

#	m/z	Abs. In	Rel. Int.
10	59.00	1785	0.85
11	60.00	601	0.29
12	60.95	959	0.46
13	62.05	2054	0.98
14	63.05	7960	3.81
15	64.15	3136	1.50
16	65.05	12011	5.75
17	66.05	1062	0.51
18	67.10	916	0.44
19	68.15	438	0.21
20	69.05	1671	0.80
21	70.10	559	0.27
22	71.10	1407	0.67
23	72.15	348	0.17
24	73.15	1422	0.68
25	74.05	6208	2.97
26	75.15	10680	5.11
27	76.05	39161	18.75
28	77.05	32239	15.44
29	78.10	7353	3.52
30	79.10	10062	4.82
31	80.10	1535	0.73
32	81.15	1056	0.51
33	82.15	574	0.27
34	83.10	1401	0.67
35	84.20	702	0.34
36	85.15	992	0.47
37	86.15	456	0.22
38	87.15	779	0.37
39	88.15	1219	0.58
40	89.15	6416	3.07
41	90.15	17171	8.22
42	91.10	208863	100.00
43	92.10	16934	8.11
44	93.00	3321	1.59
45	94.05	841	0.40
46	95.10	1343	0.64
47	96.15	528	0.25
48	97.20	977	0.47
49	98.15	896	0.43
50	99.10	390	0.19
51	100.10	342	0.16
52	101.15	660	0.32
53	102.10	3724	1.78
54	103.15	2054	0.98
55	104.10	15006	7.18
56	105.10	11479	5.50
57	106.15	8538	4.09
58	107.15	4476	2.14
59	108.15	4944	2.37
60	109.20	892	0.43
61	110.20	335	0.16
62	111.15	662	0.32
63	112.15	366	0.18
64	113.10	798	0.38
65	114.15	572	0.27
66	115.10	1082	0.52
67	116.15	748	0.36
68	117.10	1370	0.66
69	118.10	487	0.23

#	m/z	Abs. In	Rel. Int.
70	119.15	860	0.41
71	120.10	350	0.17
72	121.10	399	0.19
73	122.10	281	0.13
74	123.10	284	0.14
75	125.10	313	0.15
76	126.10	358	0.17
77	127.10	554	0.27
78	128.05	461	0.22
79	129.15	937	0.45
80	130.15	12238	5.86
81	131.10	1382	0.66
82	132.10	2113	1.01
83	133.15	459	0.22
84	136.10	274	0.13
85	137.10	343	0.16
86	138.10	514	0.25
87	139.10	1380	0.66
88	140.15	1312	0.63
89	141.10	398	0.19
90	142.10	282	0.14
91	143.05	426	0.20
92	144.10	295	0.14
93	150.00	356	0.17
94	151.05	814	0.39
95	152.05	1030	0.49
96	153.00	1066	0.51
97	154.05	610	0.29
98	155.00	1230	0.59
99	156.05	680	0.33
100	157.05	367	0.18
101	158.10	321	0.15
102	164.15	551	0.26
103	165.10	740	0.35
104	166.10	1158	0.55
105	167.05	446	0.21
106	168.10	378	0.18
107	168.95	305	0.15
108	169.90	500	0.24
109	170.90	215	0.10
110	171.90	202	0.10
111	177.15	564	0.27
112	178.15	659	0.32
113	179.15	992	0.47
114	180.20	425	0.20
115	193.20	303	0.15
116	194.20	324	0.16
117	198.20	222	0.11
118	207.20	217	0.10
119	208.20	258	0.12
120	222.15	999	0.48
121	223.15	408	0.20
122	224.15	905	0.43
123	225.20	242	0.12
124	235.20	250	0.12
125	236.15	1946	0.93
126	237.15	577	0.28
127	238.15	643	0.31
128	239.20	332	0.16
129	254.20	210	0.10

#	m/z	Abs. In	Rel. Int.
130	255.20	234	0.11
131	256.20	322	0.15
132	257.10	378	0.18
133	258.15	495	0.24
134	259.05	435	0.21
135	260.00	209	0.10
136	264.00	220	0.11
137	298.15	1504	0.72
138	299.15	1564	0.75
139	300.15	4131	1.98
140	301.25	1352	0.65
141	302.15	8270	3.96
142	303.15	1718	0.82
143	304.15	1691	0.81
144	305.20	353	0.17
145	310.20	268	0.13
146	311.35	408	0.20
147	312.30	1612	0.77
148	313.30	3425	1.64
149	314.25	2991	1.43
150	315.25	1330	0.64
151	316.20	3210	1.54
152	317.15	1051	0.50
153	318.10	711	0.34
154	319.10	354	0.17
155	340.10	206	0.10
156	341.10	238	0.11
157	342.20	420	0.20
158	343.30	382	0.18
159	344.30	1217	0.58
160	345.30	372	0.18
161	354.20	604	0.29
162	355.25	440	0.21
163	356.25	2236	1.07
164	357.25	1845	0.88
165	358.20	5396	2.58
166	359.25	1724	0.83
167	360.20	8442	4.04
168	361.15	2023	0.97
169	362.20	1754	0.84
170	363.15	374	0.18
171	387.20	204	0.10
172	388.20	217	0.10
173	389.10	410	0.20
174	390.25	296	0.14
175	391.20	708	0.34
176	392.20	353	0.17
177	393.25	511	0.24
178	394.20	210	0.10
179	404.20	295	0.14
180	406.20	508	0.24
181	449.20	220	0.11
182	462.20	220	0.11
183	463.20	220	0.11
184	464.20	406	0.19
185	466.25	662	0.32
186	467.30	231	0.11
187	468.30	218	0.10

Mass chart of compound **13**

*Synthesis of N-(benzyloxy)-2-((4-(2-chloroacetamido)phenyl)selanyl)acetamide (**14**)*

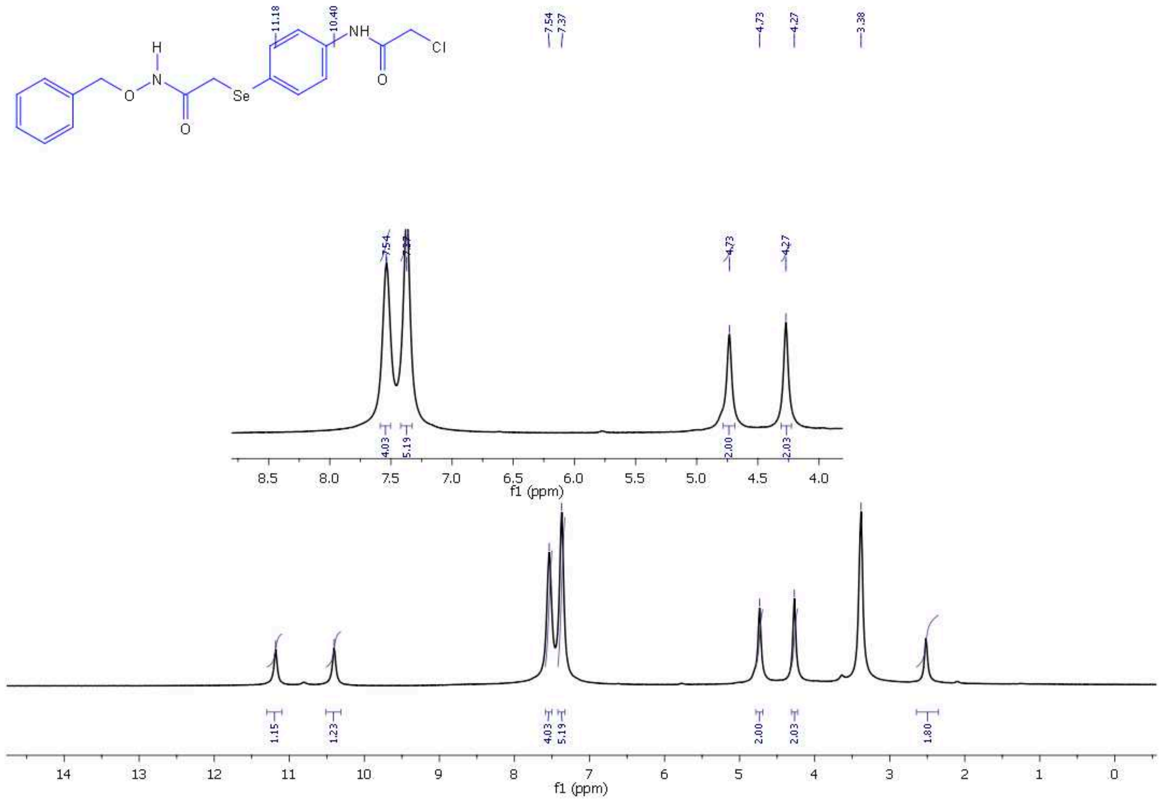

^{1}HNMR chart of compound **14**

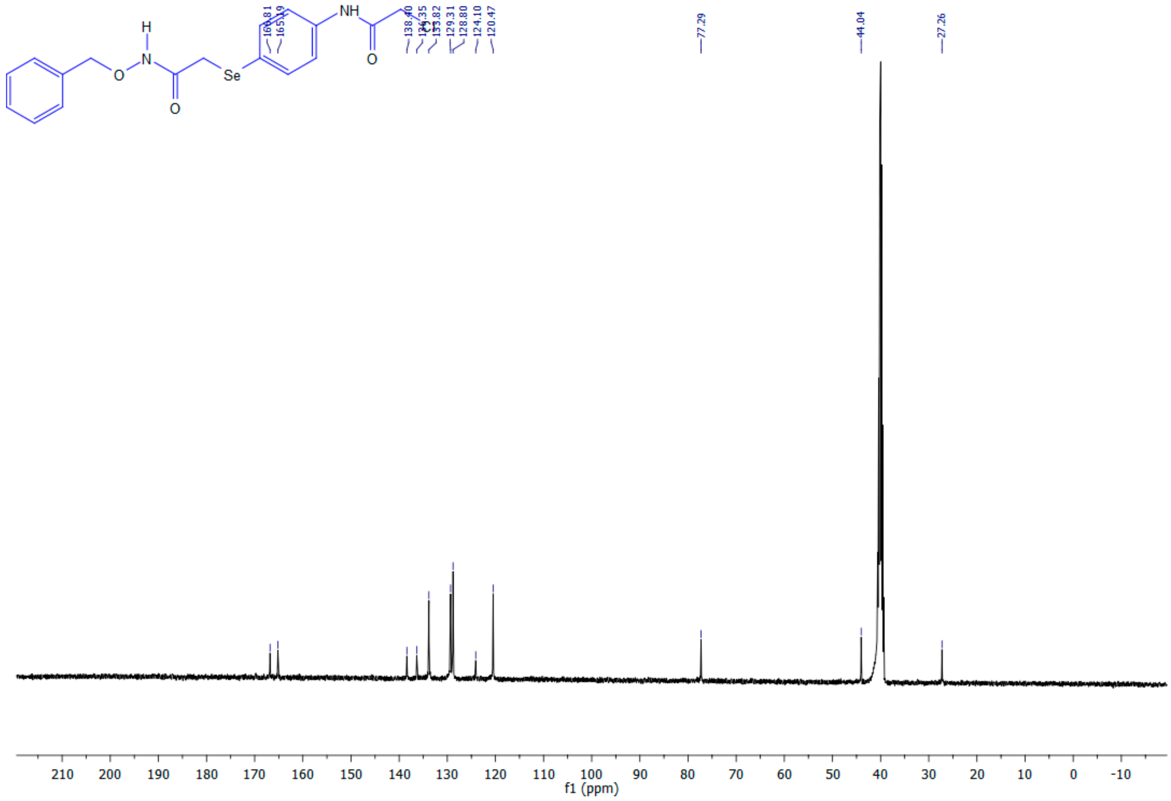

^{13}CNMR chart of compound **14**

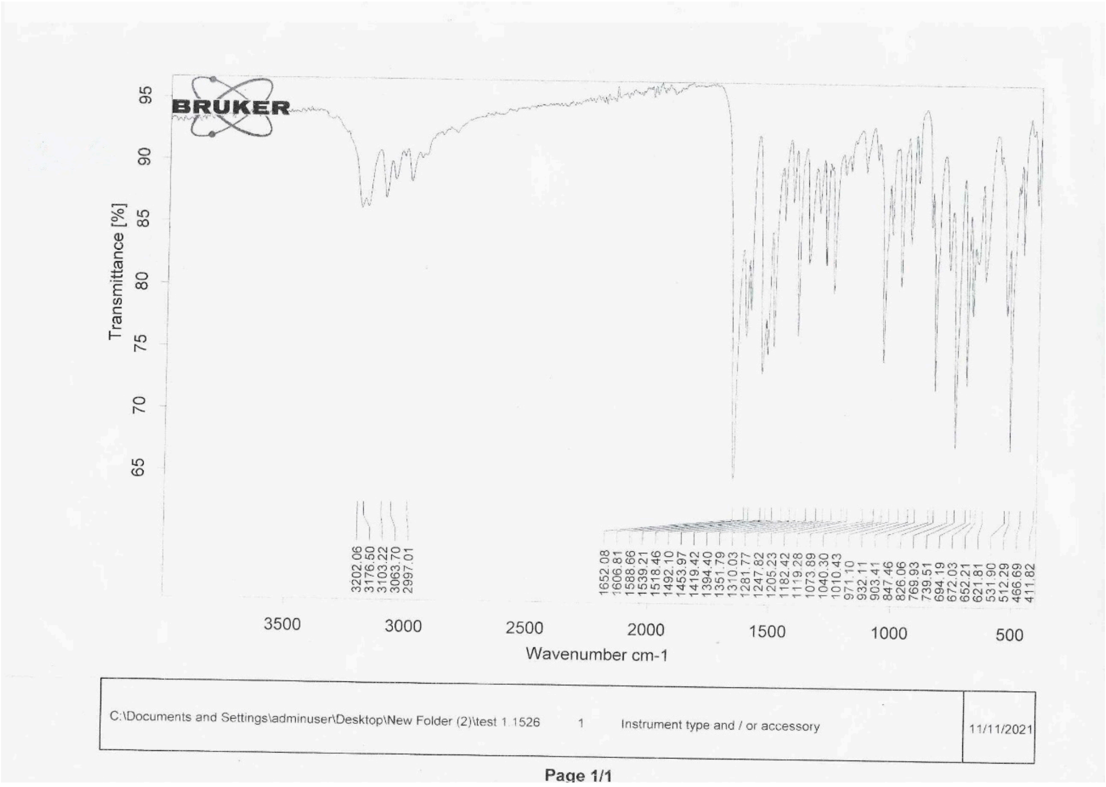

IR chart of compound **14**

06-Jan-07 18:22:50

Cairo University
Micro Analytical Center

DI Analysis
Shimadzu Qp-2010 Plus

Sample Information

Analyzed by	: Dr. Mai Younis
Analyzed	: 06/01/2007 06:15:31 م
Sample Name	: J11
Sample ID	:
Customer Name	: Dr.Mohamed Soliman - Science - Cairo
Data File	: C:\GCMSsolution\Data\Project1\J11.QGD
Org Data File	: C:\GCMSsolution\Data\Project1\J11.QGD
Method File	: C:\GCMSsolution\Data\Project1\High Temperature Op
Org Method File	: C:\GCMSsolution\Data\Project1\High Temperature Op
Report File	:
Tuning File	: C:\GCMSsolution\System\Tune1_default.qgt
$EndIf$Modified by	: Dr. Mai Younis
Modified	: 06/01/2007 06:21:22 م

Method

==== Analytical Line 1 ====

IonSourceTemp	:250.00 °C
[MS Table]	
--Group 1 - Event 1--	
Start Time	:0.00min
End Time	:10.00min
ACQ Mode	:Scan
Event Time	:0.50sec
Scan Speed	:1000
Start m/z	:50.00
End m/z	:510.00
Electron Voltage	: 70 eV
Ionization Mode	: EI

C:\GCMSsolution\Data\Project1\J11.QGD

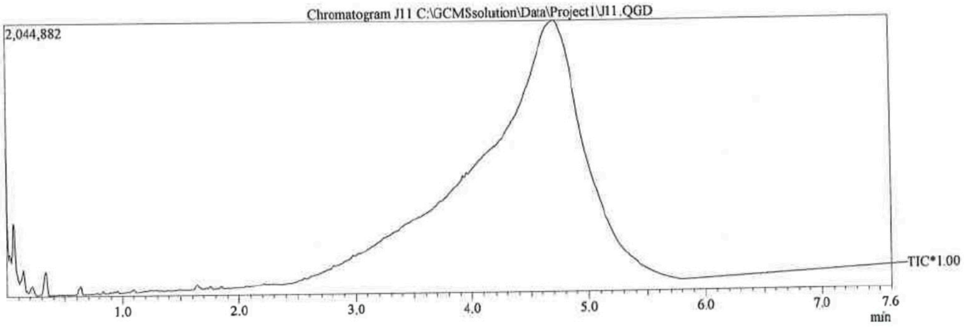

Spectrum

Line#:1 R.Time:4.7(Scan#:570)
MassPeaks:238
RawMode:Single 4.7(570) BasePeak:91(851255)
BG Mode:None Group 1 - Event 1

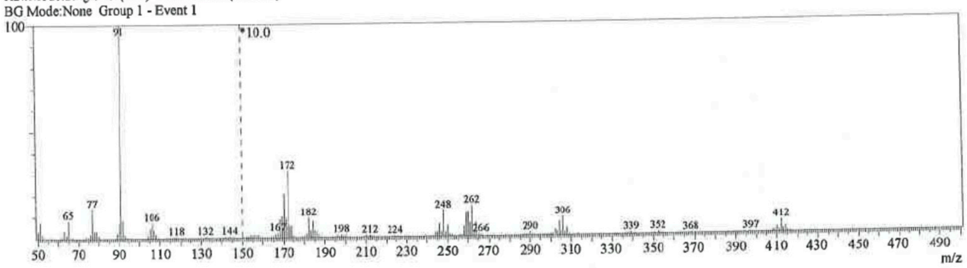

Mass Table
Line#:1 R.Time:4.7(Scan#:570)
MassPeaks:238
RawMode:Single 4.7(570) BasePeak:91(851255)
BG Mode:None Group 1 - Event 1

#	m/z	Abs. In	Rel. Int.	#	m/z	Abs. In	Rel. Int.	#	m/z	Abs. In	Rel. Int.
1	50.05	26498	3.11	4	53.05	6330	0.74	7	56.05	3355	0.39
2	51.00	62826	7.38	5	54.00	2010	0.24	8	57.10	3425	0.40
3	52.05	16634	1.95	6	55.05	3162	0.37	9	58.05	2314	0.27

#	m/z	Abs. Int	Rel. Int.	#	m/z	Abs. Int	Rel. Int.	#	m/z	Abs. Int	Rel. Int.
10	59.00	2914	0.34	79	128.05	830	0.10	148	210.10	681	0.08
11	60.00	846	0.10	80	129.05	781	0.09	149	211.00	308	0.04
12	61.05	2677	0.31	81	130.05	1057	0.12	150	212.00	692	0.08
13	62.05	8432	0.99	82	131.25	855	0.10	151	212.90	433	0.05
14	63.05	30193	3.55	83	132.15	4499	0.53	152	213.90	295	0.03
15	64.05	14147	1.66	84	133.15	3408	0.40	153	224.40	366	0.04
16	65.05	69432	8.16	85	134.20	1378	0.16	154	225.40	268	0.03
17	66.05	5685	0.67	86	135.20	559	0.07	155	229.40	263	0.03
18	67.05	3844	0.45	87	136.20	476	0.06	156	239.40	620	0.07
19	68.00	850	0.10	88	137.20	385	0.05	157	240.40	220	0.03
20	69.00	1666	0.20	89	138.10	638	0.07	158	242.40	286	0.03
21	70.10	676	0.08	90	139.15	822	0.10	159	243.10	233	0.03
22	71.10	1313	0.15	91	140.05	1877	0.22	160	244.10	1740	0.20
23	72.00	567	0.07	92	141.10	2433	0.29	161	245.10	1831	0.22
24	73.05	1953	0.23	93	142.05	3545	0.42	162	246.10	4918	0.58
25	74.05	7741	0.91	94	143.05	2860	0.34	163	247.15	1825	0.21
26	75.10	6108	0.72	95	144.05	5590	0.66	164	248.05	10251	1.20
27	76.15	16573	1.95	96	145.00	3033	0.36	165	249.10	1668	0.20
28	77.05	117544	13.81	97	146.10	1969	0.23	166	250.10	4417	0.52
29	78.10	28295	3.32	98	147.10	1087	0.13	167	251.05	826	0.10
30	79.05	29940	3.52	99	148.10	410	0.05	168	252.15	699	0.08
31	80.10	12058	1.42	100	149.10	345	0.04	169	253.20	284	0.03
32	81.05	2067	0.24	101	150.10	300	0.04	170	255.20	297	0.03
33	82.05	503	0.06	102	151.10	356	0.04	171	256.20	447	0.05
34	83.10	1474	0.17	103	152.10	687	0.08	172	257.15	449	0.05
35	84.10	961	0.11	104	153.05	614	0.07	173	258.15	3861	0.45
36	85.10	1204	0.14	105	154.05	1135	0.13	174	259.15	9088	1.07
37	86.15	822	0.10	106	155.10	850	0.10	175	260.15	9463	1.11
38	87.05	1128	0.13	107	156.10	1455	0.17	176	261.15	5402	0.63
39	88.15	1785	0.21	108	157.00	878	0.10	177	262.10	12170	1.43
40	89.10	18943	2.23	109	158.00	1070	0.13	178	263.10	2558	0.30
41	90.15	59327	6.97	110	159.00	340	0.04	179	264.10	5287	0.62
42	91.10	851255	100.00	111	160.00	266	0.03	180	265.20	1114	0.13
43	92.10	72916	8.57	112	161.00	206	0.02	181	266.15	712	0.08
44	93.05	15277	1.79	113	162.00	274	0.03	182	267.20	252	0.03
45	94.05	4637	0.54	114	164.00	314	0.04	183	269.20	226	0.03
46	95.00	2529	0.30	115	165.05	614	0.07	184	272.20	212	0.02
47	96.05	827	0.10	116	166.00	1231	0.14	185	278.20	230	0.03
48	97.10	1162	0.14	117	167.05	1808	0.21	186	280.20	254	0.03
49	98.15	1043	0.12	118	168.05	7175	0.84	187	282.20	303	0.04
50	99.00	652	0.08	119	169.05	8355	0.98	188	283.20	212	0.02
51	100.10	204	0.02	120	170.05	17341	2.04	189	285.20	290	0.03
52	101.10	489	0.06	121	171.15	7406	0.87	190	286.20	407	0.05
53	102.10	1054	0.12	122	172.05	26661	3.13	191	287.00	452	0.05
54	103.15	2371	0.28	123	173.05	4747	0.56	192	288.10	612	0.07
55	104.15	10392	1.22	124	174.05	4511	0.53	193	289.10	527	0.06
56	105.15	38854	4.56	125	175.00	791	0.09	194	290.10	1241	0.15
57	106.15	60369	7.09	126	176.00	233	0.03	195	291.00	527	0.06
58	107.15	31898	3.75	127	178.00	207	0.02	196	292.05	599	0.07
59	108.15	15831	1.86	128	179.00	254	0.03	197	293.10	290	0.03
60	109.15	2705	0.32	129	180.05	754	0.09	198	294.10	327	0.04
61	110.10	751	0.09	130	181.15	1492	0.18	199	295.10	407	0.05
62	111.10	777	0.09	131	182.10	7746	0.91	200	296.10	358	0.04
63	112.15	775	0.09	132	183.10	2647	0.31	201	297.10	460	0.05
64	113.05	1096	0.13	133	184.05	6362	0.75	202	298.10	350	0.04
65	114.10	1684	0.20	134	185.05	2702	0.32	203	299.10	417	0.05
66	115.05	2828	0.33	135	186.05	2279	0.27	204	300.15	751	0.09
67	116.15	2603	0.31	136	186.95	1198	0.14	205	301.05	620	0.07
68	117.05	5078	0.60	137	187.95	307	0.04	206	302.10	2270	0.27
69	118.05	5122	0.60	138	189.00	295	0.03	207	303.10	1636	0.19
70	119.10	2842	0.33	139	194.00	290	0.03	208	304.10	5492	0.65
71	120.10	2775	0.33	140	195.10	271	0.03	209	305.05	1488	0.17
72	121.20	1481	0.17	141	196.10	839	0.10	210	306.10	7274	0.85
73	122.15	780	0.09	142	197.00	417	0.05	211	307.05	1272	0.15
74	123.10	1151	0.14	143	198.00	918	0.11	212	308.10	3009	0.35
75	124.30	729	0.09	144	199.05	516	0.06	213	309.05	553	0.06
76	125.30	647	0.08	145	200.10	906	0.11	214	310.10	318	0.04
77	126.05	519	0.06	146	202.10	218	0.03	215	311.10	228	0.03
78	127.05	590	0.07	147	209.10	314	0.04	216	313.40	556	0.07

06-Jan-07 18:22:50

#	m/z	Abs. In	Rel. Int.
217	336.30	257	0.03
218	337.25	437	0.05
219	338.30	482	0.06
220	339.30	622	0.07
221	341.30	247	0.03
222	350.30	295	0.03
223	352.30	820	0.10
224	353.30	207	0.02

#	m/z	Abs. In	Rel. Int.
225	354.30	361	0.04
226	368.30	201	0.02
227	394.30	204	0.02
228	396.30	209	0.02
229	397.30	242	0.03
230	408.15	840	0.10
231	409.10	1046	0.12
232	410.10	2286	0.27

#	m/z	Abs. In	Rel. Int.
233	411.20	894	0.11
234	412.20	4748	0.56
235	413.10	1244	0.15
236	414.10	2274	0.27
237	415.10	535	0.06
238	416.20	462	0.05

Mass chart of compound **14**

*Synthesis of ethyl 2-(2-(4-((2-((benzyloxy)amino)-2-oxoethyl)selanyl)phenyl)hydrazineylidene)-2-cyanoacetate (**15**)*

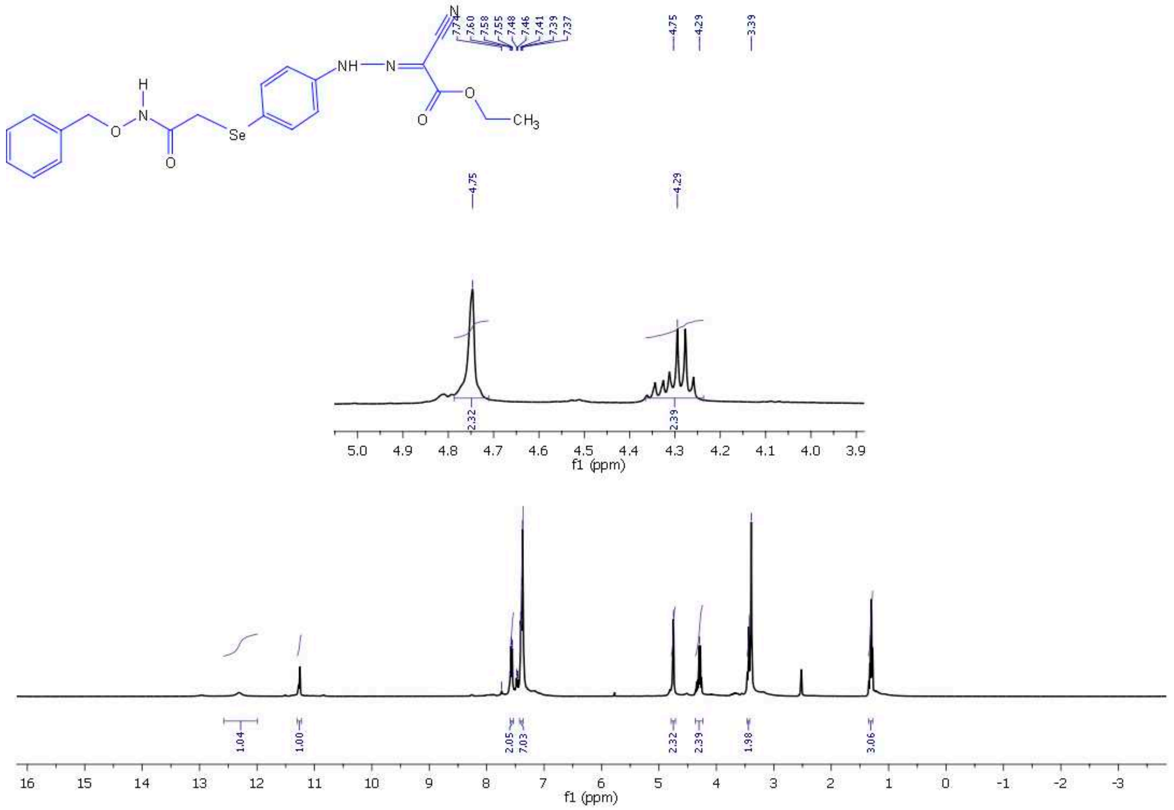

^{1}HNMR chart of compound **15**

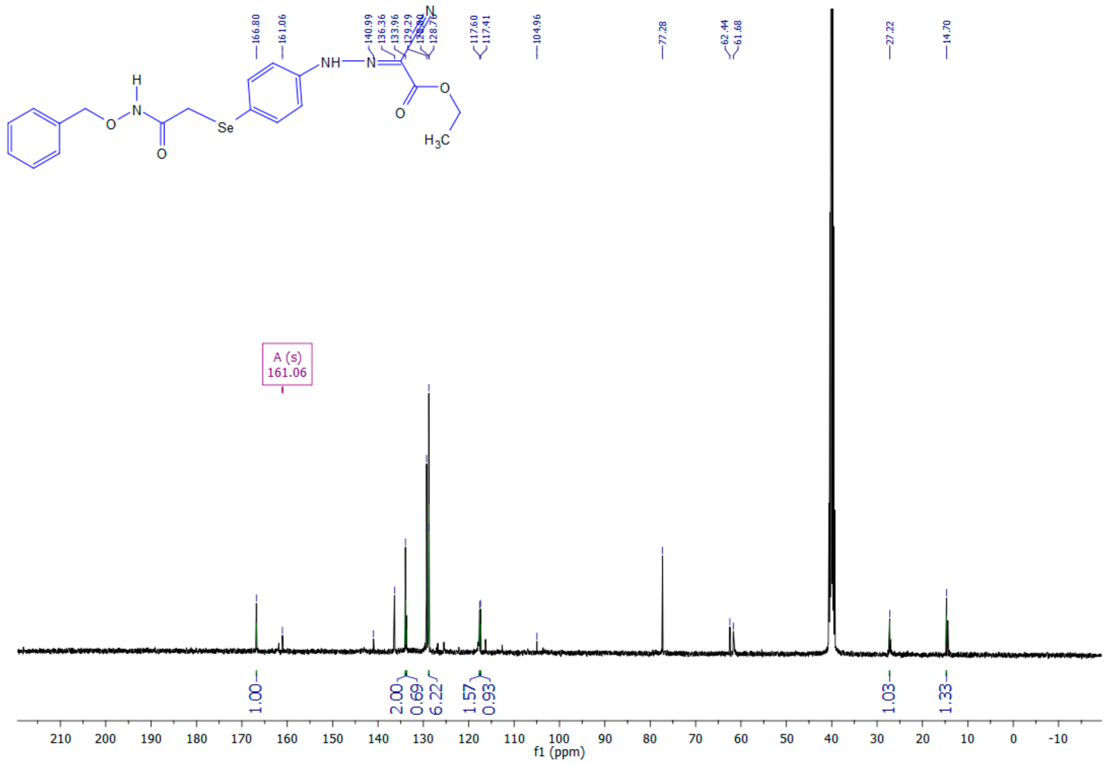

^{13}CNMR chart of compound **15**

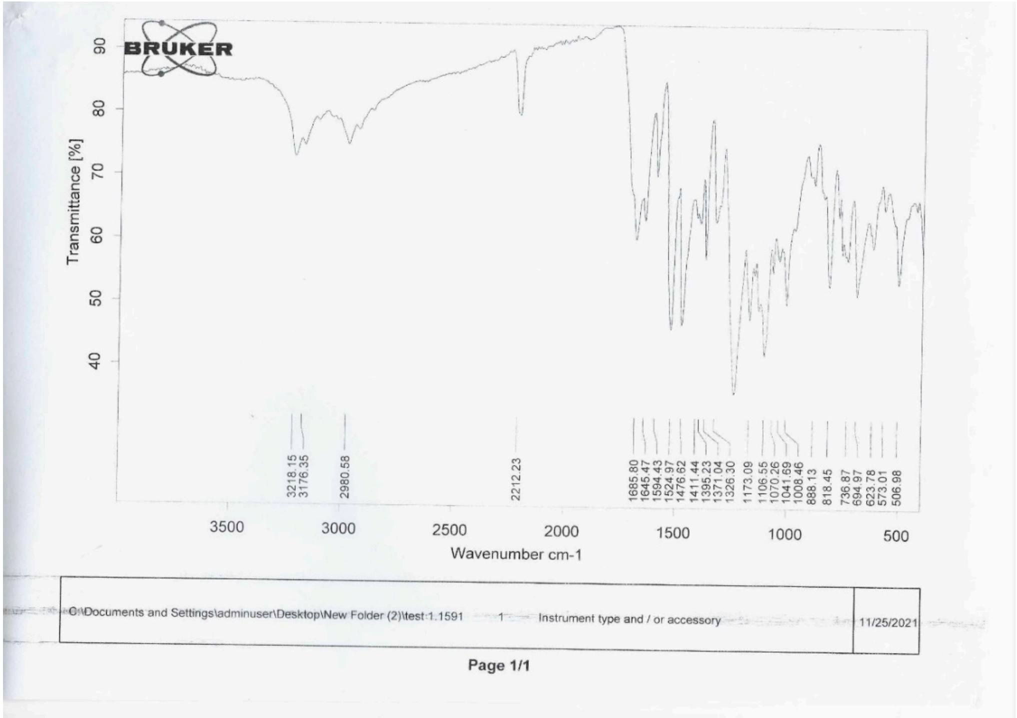

IR chart of compound **15**

Cairo University
Micro Analytical Center

DI Analysis
Shimadzu Qp-2010 Plus

Sample Information

Analyzed by	: Dr. Mai Younis
Analyzed	: 09/01/2007 06:01:28 م
Sample Name	: J13.
Sample ID	:
Customer Name	: Dr.Mohamed Soliman - Science - Cairo
Data File	: C:\GCMSsolution\Data\Project1\J13..QGD
Org Data File	: C:\GCMSsolution\Data\Project1\J13..QGD
Method File	: C:\GCMSsolution\Data\Project1\High Temperature Op
Org Method File	: C:\GCMSsolution\Data\Project1\High Temperature Op
Report File	:
Tuning File	: C:\GCMSsolution\System\Tune1_default.qgt
$EndIf$Modified by	: Dr. Mai Younis
Modified	: 09/01/2007 06:06:59 م

Method

===== Analytical Line 1 =====

IonSourceTemp	:250.00 °C
[MS Table]	
--Group 1 - Event 1--	
Start Time	:0.00min
End Time	:10.00min
ACQ Mode	:Scan
Event Time	:0.50sec
Scan Speed	:1000
Start m/z	:50.00
End m/z	:510.00
Electron Voltage	: 70 eV
Ionization Mode	: EI

C:\GCMSsolution\Data\Project1\J13..QGD

Chromatogram J13. C:\GCMSsolution\Data\Project1\J13..QGD

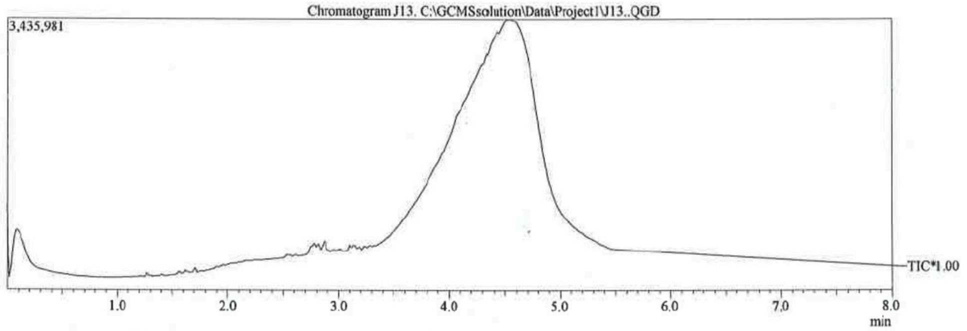

Spectrum

Line#:1 R.Time:4.5(Scan#:546)
MassPeaks:391
RawMode:Single 4.5(546) BasePeak:91(1088295)
BG Mode:None Group 1 - Event 1

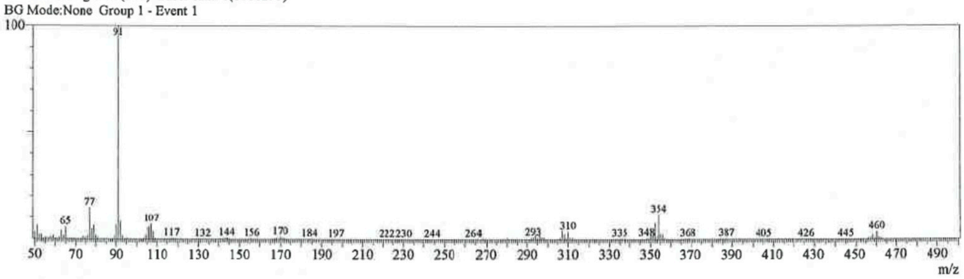

Mass Table
Line#:1 R.Time:4.5(Scan#:546)
MassPeaks:391
RawMode:Single 4.5(546) BasePeak:91(1088295)
BG Mode:None Group 1 - Event 1

#	m/z	Abs. In	Rel. Int.	#	m/z	Abs. In	Rel. Int.	#	m/z	Abs. In	Rel. Int.
1	50.00	35905	3.30	4	53.00	23239	2.14	7	56.05	9613	0.88
2	51.00	69214	6.36	5	54.00	5132	0.47	8	57.05	7929	0.73
3	52.00	25001	2.30	6	55.05	8060	0.74	9	58.05	11656	1.07

#	m/z	Abs. In	Rel. Int.	#	m/z	Abs. In	Rel. Int.	#	m/z	Abs. In	Rel. Int.
10	59.05	18779	1.73	79	128.10	1175	0.11	148	202.00	535	0.05
11	60.00	3244	0.30	80	129.15	1449	0.13	149	203.00	254	0.02
12	61.00	4092	0.38	81	130.10	1578	0.14	150	218.80	241	0.02
13	62.05	10194	0.94	82	131.10	2001	0.18	151	219.80	438	0.04
14	63.05	43298	3.98	83	132.15	2207	0.20	152	222.05	1162	0.11
15	64.05	19782	1.82	84	133.20	1822	0.17	153	222.95	299	0.03
16	65.05	61036	5.61	85	134.20	1015	0.09	154	223.95	510	0.05
17	66.05	5760	0.53	86	135.15	882	0.08	155	225.00	340	0.03
18	67.05	5302	0.49	87	136.10	662	0.06	156	226.05	688	0.06
19	68.05	3181	0.29	88	137.15	839	0.08	157	227.05	670	0.06
20	69.05	5267	0.48	89	138.15	1015	0.09	158	228.05	1247	0.11
21	70.05	2000	0.18	90	139.05	1126	0.10	159	229.20	743	0.07
22	71.10	4175	0.38	91	140.00	2246	0.21	160	230.15	1767	0.16
23	72.05	1210	0.11	92	141.05	2955	0.27	161	231.25	612	0.06
24	73.05	5219	0.48	93	142.00	4098	0.38	162	232.20	641	0.06
25	74.00	12913	1.19	94	143.05	4491	0.41	163	233.20	614	0.06
26	75.05	9770	0.90	95	144.05	7770	0.71	164	234.25	626	0.06
27	76.15	18664	1.71	96	145.10	2374	0.22	165	235.25	319	0.03
28	77.05	159933	14.70	97	146.05	1764	0.16	166	236.25	838	0.08
29	78.05	51062	4.69	98	147.05	1009	0.09	167	237.20	388	0.04
30	79.05	70177	6.45	99	148.00	631	0.06	168	238.20	255	0.02
31	80.05	16860	1.55	100	149.00	385	0.04	169	239.30	546	0.05
32	81.05	9962	0.92	101	150.10	527	0.05	170	240.20	217	0.02
33	82.10	1934	0.18	102	151.05	583	0.05	171	241.20	594	0.05
34	83.10	3868	0.36	103	152.00	1183	0.11	172	242.20	258	0.02
35	84.05	3207	0.29	104	153.05	1318	0.12	173	243.25	319	0.03
36	85.10	3174	0.29	105	154.05	2062	0.19	174	244.20	1462	0.13
37	86.10	1415	0.13	106	155.05	1568	0.14	175	245.30	444	0.04
38	87.05	2226	0.20	107	156.05	4930	0.45	176	246.25	435	0.04
39	88.15	2736	0.25	108	157.00	2012	0.18	177	247.25	962	0.09
40	89.10	19423	1.78	109	158.05	2172	0.20	178	248.25	814	0.07
41	90.15	71749	6.59	110	159.05	1157	0.11	179	249.15	411	0.04
42	91.10	108829	100.00	111	160.00	542	0.05	180	250.15	989	0.09
43	92.10	91846	8.44	112	161.00	388	0.04	181	251.20	548	0.05
44	93.00	16750	1.54	113	162.00	466	0.04	182	252.25	500	0.05
45	94.05	4139	0.38	114	163.00	391	0.04	183	253.10	470	0.04
46	95.05	4899	0.45	115	163.95	512	0.05	184	254.20	462	0.04
47	96.10	1862	0.17	116	165.05	997	0.09	185	255.20	742	0.07
48	97.15	2742	0.25	117	166.00	2961	0.27	186	256.20	620	0.06
49	98.15	2858	0.26	118	167.05	4452	0.41	187	257.20	1009	0.09
50	99.10	1095	0.10	119	168.00	7623	0.70	188	258.20	1337	0.12
51	100.15	662	0.06	120	169.00	5490	0.50	189	259.20	721	0.07
52	101.15	1018	0.09	121	170.00	12964	1.19	190	260.15	755	0.07
53	102.15	2509	0.23	122	171.00	7519	0.69	191	261.15	517	0.05
54	103.15	5193	0.48	123	172.00	6023	0.55	192	262.10	1423	0.13
55	104.15	21665	1.99	124	172.95	1687	0.16	193	263.15	579	0.05
56	105.10	59895	5.50	125	174.00	924	0.08	194	264.10	2279	0.21
57	106.10	62922	5.78	126	175.00	489	0.04	195	265.15	1872	0.17
58	107.15	78472	7.21	127	176.00	415	0.04	196	266.10	4090	0.38
59	108.15	40703	3.74	128	177.00	286	0.03	197	267.10	1380	0.13
60	109.15	4299	0.40	129	178.00	298	0.03	198	268.10	6807	0.63
61	110.10	1364	0.13	130	179.00	385	0.04	199	269.05	1910	0.18
62	111.20	1438	0.13	131	179.95	813	0.07	200	270.10	1612	0.15
63	112.05	1219	0.11	132	181.05	1052	0.10	201	271.10	578	0.05
64	113.05	1459	0.13	133	182.00	1294	0.12	202	272.10	506	0.05
65	114.05	2413	0.22	134	183.00	1018	0.09	203	273.10	322	0.03
66	115.05	4121	0.38	135	184.00	1881	0.17	204	274.10	508	0.05
67	116.05	3428	0.31	136	184.95	1072	0.10	205	275.10	380	0.03
68	117.05	5925	0.54	137	185.95	837	0.08	206	276.10	1478	0.14
69	118.05	4438	0.41	138	187.00	492	0.05	207	277.10	1508	0.14
70	119.10	3518	0.32	139	188.00	319	0.03	208	278.10	3412	0.31
71	120.10	2058	0.19	140	189.00	215	0.02	209	279.10	1281	0.12
72	121.15	1299	0.12	141	193.00	297	0.03	210	280.10	6390	0.59
73	122.15	1090	0.10	142	193.90	562	0.05	211	281.05	1544	0.14
74	123.20	1366	0.13	143	194.90	591	0.05	212	282.10	2294	0.21
75	124.20	670	0.06	144	195.85	654	0.06	213	283.00	895	0.08
76	125.15	505	0.05	145	197.00	806	0.07	214	284.05	525	0.05
77	126.15	589	0.05	146	197.80	682	0.06	215	285.10	468	0.04
78	127.15	900	0.08	147	198.90	470	0.04	216	286.10	326	0.03

#	m/z	Abs. In	Rel. Int.
217	287.10	225	0.02
218	288.10	388	0.04
219	289.10	449	0.04
220	290.10	1342	0.12
221	291.15	720	0.07
222	292.10	9666	0.89
223	293.10	9772	0.90
224	294.10	25895	2.38
225	295.10	6433	0.59
226	296.10	51970	4.78
227	297.05	10872	1.00
228	298.10	10124	0.93
229	299.05	2325	0.21
230	300.05	482	0.04
231	301.00	255	0.02
232	302.00	404	0.04
233	302.95	478	0.04
234	304.25	1031	0.09
235	305.35	1173	0.11
236	306.25	8371	0.77
237	307.25	45850	4.21
238	308.25	27017	2.48
239	309.15	7888	0.72
240	310.10	39802	3.66
241	311.05	8289	0.76
242	312.10	8414	0.77
243	313.20	2913	0.27
244	314.15	773	0.07
245	315.15	373	0.03
246	315.90	276	0.03
247	316.85	386	0.04
248	317.90	399	0.04
249	319.05	578	0.05
250	320.10	583	0.05
251	321.10	777	0.07
252	322.05	1112	0.10
253	323.10	974	0.09
254	324.10	1318	0.12
255	325.15	1202	0.11
256	326.10	1844	0.17
257	327.40	951	0.09
258	328.35	480	0.04
259	329.10	278	0.03
260	330.05	339	0.03
261	331.15	781	0.07
262	332.15	990	0.09
263	333.15	2090	0.19
264	334.10	2071	0.19
265	335.15	3842	0.35
266	336.15	3157	0.29
267	337.10	4551	0.42
268	338.10	1945	0.18
269	339.25	1566	0.14
270	340.45	782	0.07
271	341.40	818	0.08
272	342.35	561	0.05
273	343.40	295	0.03
274	345.25	462	0.04
275	346.20	660	0.06
276	347.10	270	0.02
277	348.15	6118	0.56
278	349.15	4932	0.45
279	350.15	33749	3.10
280	351.15	26545	2.44
281	352.15	81401	7.48
282	353.25	18543	1.70
283	354.15	126433	11.62
284	355.10	25895	2.38
285	356.15	25958	2.39
286	357.05	4841	0.44
287	358.20	778	0.07
288	359.40	463	0.04
289	360.40	434	0.04
290	361.25	390	0.04
291	362.15	527	0.05
292	363.40	454	0.04
293	364.40	441	0.04
294	365.35	958	0.09
295	366.35	867	0.08
296	367.40	1481	0.14
297	368.40	3076	0.28
298	369.35	1845	0.17
299	370.30	999	0.09
300	371.20	794	0.07
301	372.25	796	0.07
302	373.25	649	0.06
303	374.20	500	0.05
304	375.25	533	0.05
305	376.20	764	0.07
306	377.20	614	0.06
307	378.20	868	0.08
308	379.25	1023	0.09
309	380.15	1408	0.13
310	381.20	1009	0.09
311	382.25	1402	0.13
312	383.25	2090	0.19
313	384.20	1946	0.18
314	385.20	3260	0.30
315	386.20	2054	0.19
316	387.20	4902	0.45
317	388.20	2028	0.19
318	389.20	1498	0.14
319	390.25	876	0.08
320	391.20	641	0.06
321	392.20	452	0.04
322	393.25	822	0.08
323	394.15	865	0.08
324	395.25	680	0.06
325	396.20	770	0.07
326	397.15	473	0.04
327	398.10	385	0.04
328	399.10	409	0.04
329	400.10	380	0.03
330	401.10	404	0.04
331	402.10	418	0.04
332	403.10	460	0.04
333	404.20	678	0.06
334	405.20	1041	0.10
335	406.10	714	0.07
336	407.15	636	0.06
337	408.15	1313	0.12
338	409.15	1261	0.12
339	410.10	1718	0.16
340	411.15	1078	0.10
341	412.10	1542	0.14
342	413.20	1065	0.10
343	414.05	713	0.07
344	415.10	1226	0.11
345	416.10	516	0.05
346	417.05	555	0.05
347	418.10	388	0.04
348	419.10	386	0.04
349	420.10	298	0.03
350	421.05	338	0.03
351	422.00	438	0.04
352	423.05	696	0.06
353	424.00	836	0.08
354	425.05	594	0.05
355	426.05	1043	0.10
356	427.05	578	0.05
357	428.00	674	0.06
358	429.00	345	0.03
359	430.10	598	0.05
360	431.05	401	0.04
361	432.00	599	0.06
362	432.95	390	0.04
363	434.10	383	0.04
364	435.10	258	0.02
365	436.75	321	0.03
366	437.70	484	0.04
367	438.90	462	0.04
368	439.80	366	0.03
369	441.05	496	0.05
370	442.05	514	0.05
371	443.05	902	0.08
372	444.05	407	0.04
373	445.20	1599	0.15
374	446.15	507	0.05
375	447.70	430	0.04
376	448.70	351	0.03
377	449.70	510	0.05
378	450.50	255	0.02
379	451.50	777	0.07
380	452.70	409	0.04
381	454.20	974	0.09
382	455.25	365	0.03
383	456.25	7785	0.72
384	457.25	8651	0.79
385	458.20	20503	1.88
386	459.25	5902	0.54
387	460.20	38226	3.51
388	461.25	10119	0.93
389	462.20	7446	0.68
390	463.25	1843	0.17
391	464.20	201	0.02

Mass chart of compound **15**

Synthesis of N-(benzyloxy)-2-((4-((4-hydroxyphenyl)diazenyl)phenyl)selanyl)acetamide ***(16)***

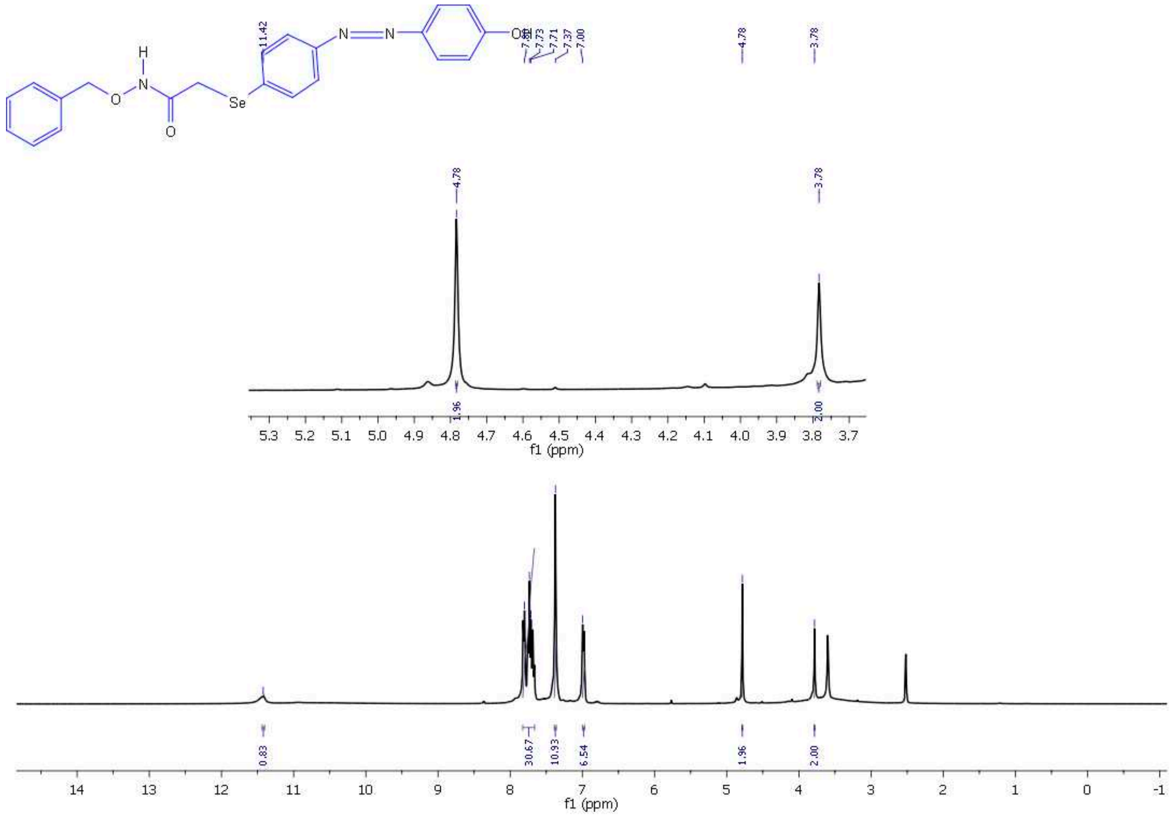

^{1}HNMR chart of compound **16**

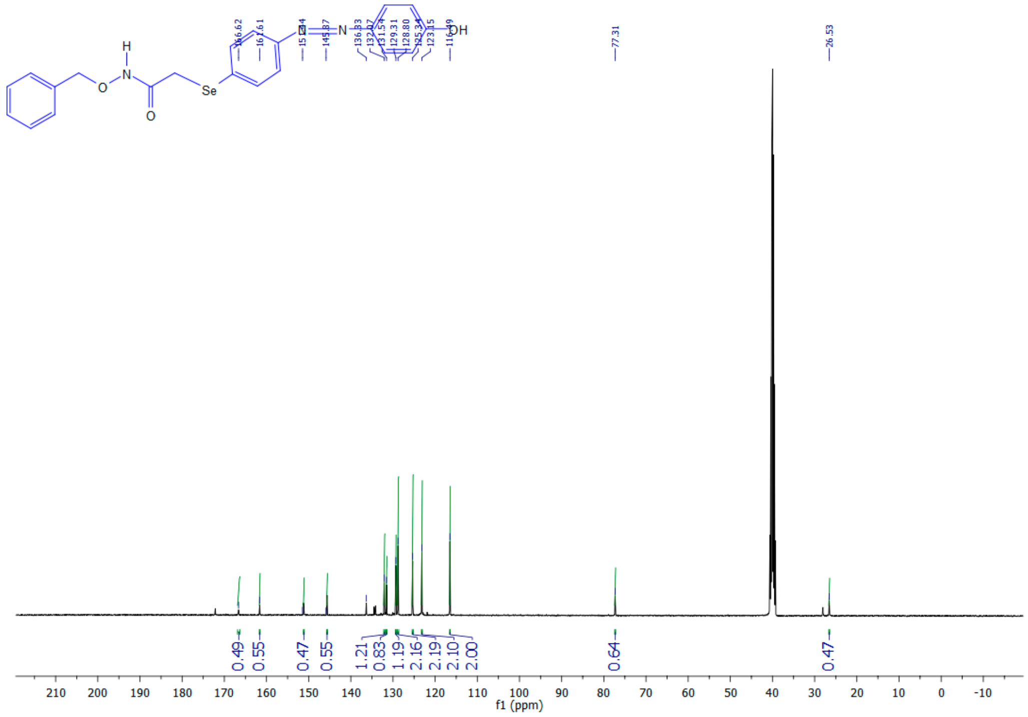

^{13}CNMR chart of compound **16**

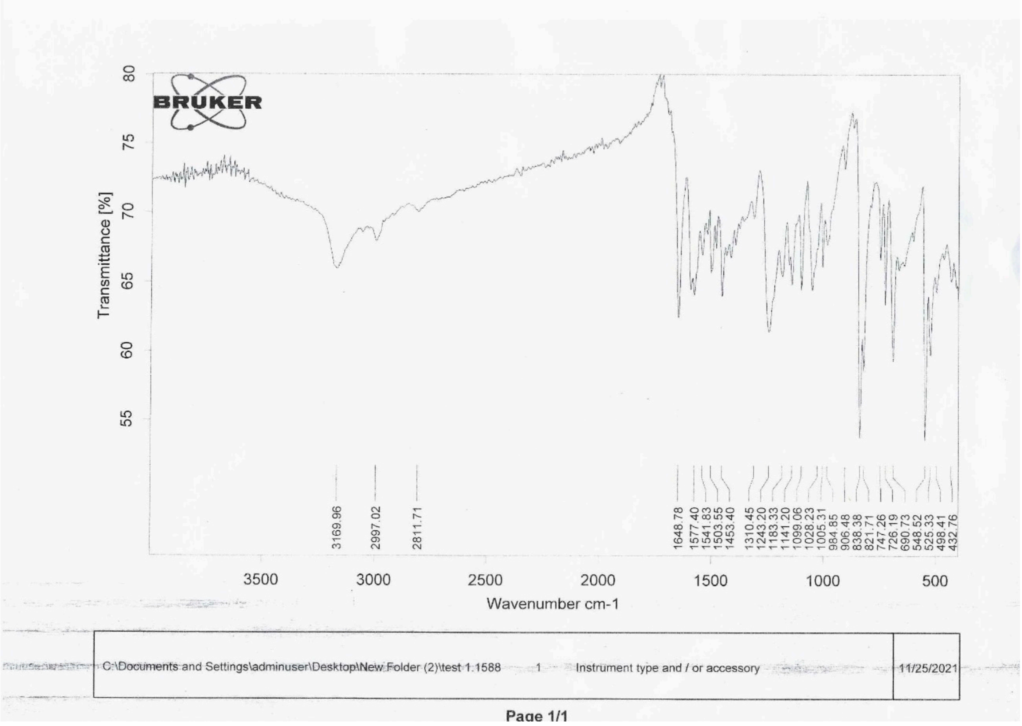

IR chart of compound **16**

Cairo University
Micro Analytical Center

DI Analysis
Shimadzu Qp-2010 Plus

Sample Information

Analyzed by	: Dr. Mai Younis
Analyzed	: 03/01/2007 06:29:34 م
Sample Name	: J14
Sample ID	:
Customer Name	: Dr.Mohamed Soliman - Science - Cairo
Data File	: C:\GCMSsolution\Data\Project1\J14.QGD
Org Data File	: C:\GCMSsolution\Data\Project1\J14.QGD
Method File	: C:\GCMSsolution\Data\Project1\High Temperature Op
Org Method File	: C:\GCMSsolution\Data\Project1\High Temperature Op
Report File	:
Tuning File	: C:\GCMSsolution\System\Tune1_default.qgt
$EndIf$Modified by	: Dr. Mai Younis
Modified	: 03/01/2007 06:37:07 م

Method

==== Analytical Line 1 ====

IonSourceTemp	:250.00 °C
[MS Table]	
--Group 1 - Event 1--	
Start Time	:0.00min
End Time	:10.00min
ACQ Mode	:Scan
Event Time	:0.50sec
Scan Speed	:1000
Start m/z	:50.00
End m/z	:510.00
Electron Voltage	:70 eV
Ionization Mode	:EI

C:\GCMSsolution\Data\Project1\J14.QGD

Chromatogram J14 C:\GCMSsolution\Data\Project1\J14.QGD

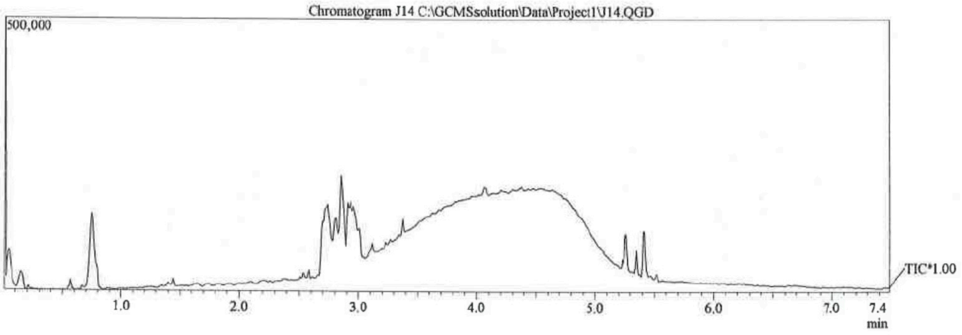

Spectrum

Line#:1 R.Time:4.7(Scan#:560)
MassPeaks:91
RawMode:Single 4.7(560) BasePeak:91(39273)
BG Mode:None Group 1 - Event 1

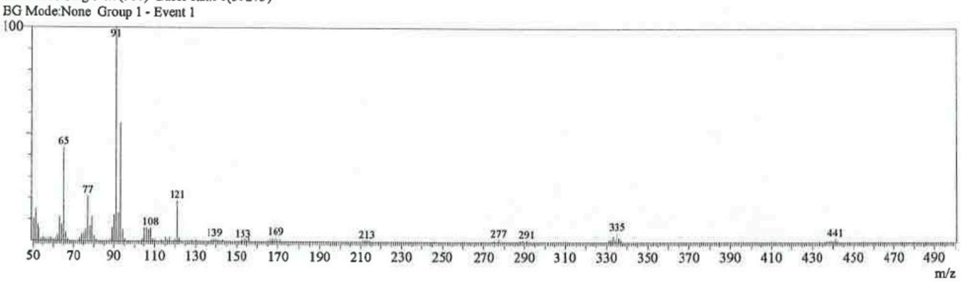

Mass Table
Line#:1 R.Time:4.7(Scan#:560)
MassPeaks:91
RawMode:Single 4.7(560) BasePeak:91(39273)
BG Mode:None Group 1 - Event 1

#	m/z	Abs. In	Rel. Int.	#	m/z	Abs. In	Rel. Int.	#	m/z	Abs. In	Rel. Int.
1	50.05	4010	10.21	4	53.05	2166	5.52	7	56.00	471	1.20
2	51.05	5861	14.92	5	54.00	393	1.00	8	57.00	305	0.78
3	52.05	2891	7.36	6	55.05	671	1.71	9	58.10	580	1.48

03-Jan-07 18:45:47

#	m/z	Abs. In	Rel. Int.	#	m/z	Abs. In	Rel. Int.	#	m/z	Abs. In	Rel. Int.
10	59.05	675	1.72	38	106.10	2369	6.03	66	168.05	462	1.18
11	60.10	278	0.71	39	107.10	2078	5.29	67	168.95	749	1.91
12	61.10	543	1.38	40	108.10	2433	6.20	68	170.00	300	0.76
13	62.10	1214	3.09	41	109.10	417	1.06	69	171.00	337	0.86
14	63.05	4420	11.25	42	110.10	362	0.92	70	211.00	234	0.60
15	64.15	2981	7.59	43	113.10	202	0.51	71	212.00	209	0.53
16	65.05	17265	43.96	44	115.05	745	1.90	72	213.00	244	0.62
17	66.05	1585	4.04	45	116.00	204	0.52	73	214.00	233	0.59
18	67.00	335	0.85	46	117.05	776	1.98	74	275.00	210	0.53
19	73.15	441	1.12	47	119.30	326	0.83	75	277.20	522	1.33
20	74.10	1262	3.21	48	120.15	461	1.17	76	288.20	215	0.55
21	75.10	1551	3.95	49	121.15	7432	18.92	77	289.20	278	0.71
22	76.15	2187	5.57	50	122.15	632	1.61	78	291.20	361	0.92
23	77.10	8223	20.94	51	128.10	284	0.72	79	331.15	461	1.17
24	78.15	2585	6.58	52	130.10	399	1.02	80	332.15	458	1.17
25	79.10	4329	11.02	53	138.10	308	0.78	81	333.15	1072	2.73
26	80.10	905	2.30	54	139.25	474	1.21	82	334.10	462	1.18
27	81.10	314	0.80	55	140.20	262	0.67	83	335.20	1775	4.52
28	88.15	284	0.72	56	141.20	305	0.78	84	336.15	756	1.92
29	89.15	2445	6.23	57	143.20	202	0.51	85	337.10	422	1.07
30	90.15	4810	12.25	58	152.20	311	0.79	86	437.10	218	0.56
31	91.10	39273	100.00	59	153.05	326	0.83	87	438.10	258	0.66
32	92.15	5294	13.48	60	154.10	561	1.43	88	439.10	297	0.76
33	93.10	21678	55.20	61	155.00	262	0.67	89	440.10	214	0.54
34	94.10	2052	5.22	62	156.05	994	2.53	90	441.20	713	1.82
35	95.10	414	1.05	63	165.00	249	0.63	91	442.20	238	0.61
36	104.15	434	1.11	64	166.00	322	0.82				
37	105.10	2386	6.08	65	167.05	568	1.45				

Mass chart of compound **16**

References

1. El-Senduny, F.F.; Shabana, S.M.; Rösel, D.; Brabek, J.; Althagafi, I.; Angeloni, G.; Manolikakes, G.; Shaaban, S. Urea-functionalized organoselenium compounds as promising anti-HepG2 and apoptosis-inducing agents. *Future Medicinal Chemistry* **2021**, *13*, 1655-1677.
2. Shaaban, S.; Shabana, S.M.; Al-Faiyz, Y.S.; Manolikakes, G.; El-Senduny, F.F. Enhancing the chemosensitivity of HepG2 cells towards cisplatin by organoselenium pseudopeptides. *Bioorg Chem* **2021**, *109*, 104713, doi:10.1016/j.bioorg.2021.104713.
3. Shaaban, S.; Zarrouk, A.; Vervandier-Fasseur, D.; S.Al-Faiyz, Y.; El-Sawy, H.; Althagafi, I.; Andreoletti, P.; Cherkaoui-Malki, M. Cytoprotective organoselenium compounds for oligodendrocytes. *Arabian Journal of Chemistry* **2021**, *14*, doi:10.1016/j.arabjc.2021.103051.
4. Shaaban, S.; Negm, A.; Sobh, M.A.; Wessjohann, L.A. Expeditious Entry to Functionalized Pseudo-peptidic Organoselenide Redox Modulators via Sequential Ugi/SN Methodology. *Anticancer Agents Med Chem* **2016**, *16*, 621-632, doi:10.2174/1871520615666150916092035.
5. Shaaban, S.; Negm, A.; Ashmawy, A.M.; Ahmed, D.M.; Wessjohann, L.A. Combinatorial synthesis, in silico, molecular and biochemical studies of tetrazole-derived organic selenides with increased selectivity against hepatocellular carcinoma. **2016**, *122*, 55-71, doi:https://doi.org/10.1016/j.ejmech.2016.06.005.
6. Shaaban, S.; Ashmawy, A.M.; Negm, A.; Wessjohann, L.A. Synthesis and biochemical studies of novel organic selenides with increased selectivity for hepatocellular carcinoma and breast adenocarcinoma. *Eur J Med Chem* **2019**, *179*, 515-526, doi:10.1016/j.ejmech.2019.06.075.
